# Development of a peer-supported, self-management intervention for people following mental health crisis

**DOI:** 10.1186/s13104-017-2900-6

**Published:** 2017-11-09

**Authors:** Alyssa Milton, Brynmor Lloyd-Evans, Kate Fullarton, Nicola Morant, Bethan Paterson, David Hindle, Kathleen Kelly, Oliver Mason, Marissa Lambert, Sonia Johnson

**Affiliations:** 1 Division of Psychiatry, University College London, 6th Floor, Maple House, 149 Tottenham Court Road, London, W1T 7NF UK; 2 Brain and Mind Centre, The University of Sydney, Sydney, NSW 2006 Australia; 3 Oxford Health NHS Foundation Trust, Barnes Unit, John Radcliffe Hospital, Oxford, OX3 9DU UK; 4 Research Department of Clinical, Educational and Health Psychology, University College London, London, WC1N 6BT UK; 5 School of Psychology, University of Surrey, Guildford, Surrey GU2 7XH UK; 6 The Institute of Mental Health, University of Nottingham Innovation Park, Triumph Road, Nottingham, NG7 2TU UK

**Keywords:** Programme development, Peer support, Self-management, Complex interventions, Mental health, Recovery, Crisis resolution teams

## Abstract

**Background:**

A documented gap in support exists for service users following discharge from acute mental health services, and structured interventions to reduce relapse are rarely provided. Peer-facilitated self-management interventions have potential to meet this need, but evidence for their effectiveness is limited. This paper describes the development of a peer-provided self-management intervention for mental health service users following discharge from crisis resolution teams (CRTs).

**Methods:**

A five-stage iterative mixed-methods approach of sequential data collection and intervention development was adopted, following the development and piloting stages of the MRC framework for developing and evaluating complex interventions. Evidence review (stage 1) included systematic reviews of both peer support and self-management literature. Interviews with CRT service users (n = 41) regarding needs and priorities for support following CRT discharge were conducted (stage 2). Focus group consultations (n = 12) were held with CRT service-users, staff and carers to assess the acceptability and feasibility of a proposed intervention, and to refine intervention organisation and content (stage 3). Qualitative evaluation of a refined, peer-provided, self-management intervention involved qualitative interviews with CRT service user participants (n = 9; n = 18) in feasibility testing (stage 4) and a pilot trial (stage 5), and a focus group at each stage with the peer worker providers (n = 4).

**Results:**

Existing evidence suggests self-management interventions can reduce relapse and improve recovery. Initial interviews and focus groups indicated support for the overall purpose and planned content of a recovery-focused self-management intervention for people leaving CRT care adapted from an existing resource: The personal recovery plan (developed by Repper and Perkins), and for peer support workers (PSWs) as providers. Participant feedback after feasibility testing was positive regarding facilitation of the intervention by PSWs; however, the structured self-management booklet was underutilised. Modifications to the self-management intervention manual and PSWs’ training were made before piloting, which confirmed the acceptability and feasibility of the intervention for testing in a future, definitive trial.

**Conclusions:**

A manualised intervention and operating procedures, focusing on the needs and priorities of the target client group, have been developed through iterative stages of intervention development and feedback for testing in a trial context.

*Trial Registration* ISRCTN01027104 date of registration: 11/10/2012

**Electronic supplementary material:**

The online version of this article (10.1186/s13104-017-2900-6) contains supplementary material, which is available to authorized users.

## Background

Crisis resolution teams (CRTs), also referred to as home treatment teams, provide rapid assessment for service users experiencing mental health crises and, where possible, offer brief, intensive home treatment as an alternative to acute admission [1]. Since their adoption into the national health service (NHS) plan [2], CRTs are now available in every NHS trust in England [3] and have also been implemented nationally in Norway and Flemish Belgium [4]. The history of the development of CRT services [4], and detailed specification of a CRT service model [5] have been previously reported. Trial evidence suggests CRTs can be an effective service model, which reduce inpatient admissions and increase service users’ satisfaction with acute care [6, 7]. However, when scaled up to national level in England, CRTs’ implementation has been variable [3, 8] and their impact on admission rates equivocal [9]. Service users report experiencing CRT support as ending very abruptly, and there is a documented gap in support for mental health service-users post discharge [10]. CRT support rarely includes helping service users to develop strategies to support recovery beyond the immediate crisis and to avert future crises [5]. There is a lack of information about rates of readmission to acute care nationally following a period of CRT support, but high rates have been reported—just over 50% within 1 year—in a recent study in two inner London NHS trusts [11].

Mental health self-management programmes have been proposed as a means to help service users to learn skills to manage their psychological wellbeing and avoid future crises with lower levels of service input [12]. Self-management is a problem-solving approach that is skill-based and can be taught [13]. It is defined in health literature as a collaborative learning process which supports the individual’s ability to manage the symptoms, treatment, physical and psychosocial consequences and lifestyle changes inherent in living with a chronic condition [14]. Programmes typically encourage service users to become an expert in their own recovery [15] and frequently include relapse prevention planning within programmes. This usually involves identifying signs of a crisis and developing coping strategies to respond to them [12]. There is evidence across different mental health conditions for the effectiveness of various forms of self-management programmes [16–19]. Evidence and expert consensus suggests that supported self-management programmes (with guidance from a health professional or other helper) are preferable to independent self-management for people with serious mental health conditions [20]. Self-management programmes where support is provided by a peer support worker (PSW), who has themselves experienced mental ill health, have shown promising evidence of effectiveness [21–23].

In this context, there is reason to hope that support, immediately post-CRT discharge, with managing recovery and relapse planning may be a helpful addition to standard CRT care. In the UK, the employment of PSWs to deliver self-management support to service users is becoming increasingly common within NHS services [24]. To our knowledge, however, there have been no evaluations of peer-provided, self-management interventions for people leaving CRT care. In this paper, following medical research council (MRC) guidance for the development and evaluation of complex interventions [25], we report the iterative development and feasibility testing of a peer supported, self-management intervention for people leaving CRT services for use in a definitive randomised controlled trial. The protocol for this trial (ISRCTN01027104) is reported separately [26].

## Methods

**Fig. 1:**
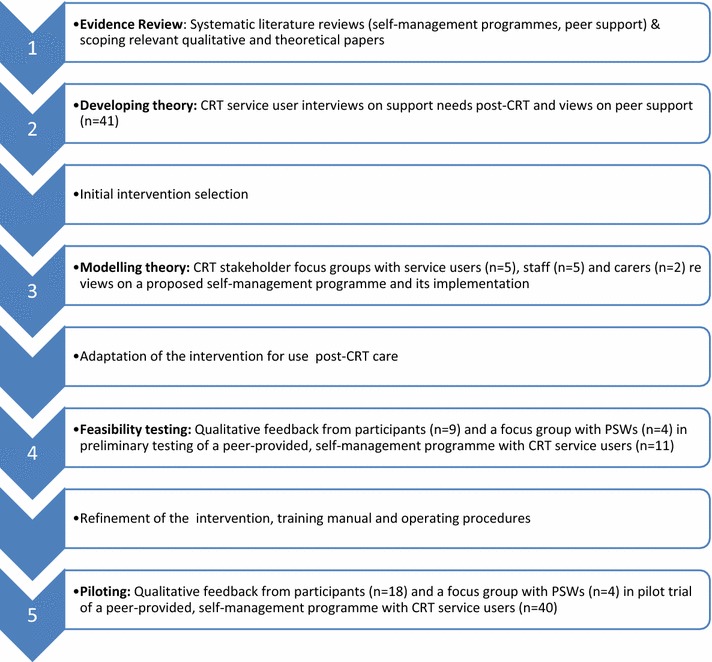
Process of developing a peer-supported, self-management programme for people following CRT care

This paper describes five stages of developing a peer-provided, self-management intervention for people following a period of CRT support. Stages 1–3 correspond to the “development” phase of the MRC framework for developing and evaluating complex interventions [25]. Stages 4 and 5 correspond to the “feasibility/piloting” stage of the framework. In stages 1 and 2, evidence reviews and interviews with CRT service users regarding views on peer support and priorities for support following CRT care were conducted. Findings informed our initial selection of a self-management resource for use in the CORE Study trial which was made in collaboration with expert reference groups of CRT staff, service users and carers. Stage 3 then involved stakeholder consultations via focus groups, regarding acceptability and implementation of the proposed intervention, and subsequent adaptations of the selected self-management resource for use in a peer-supported programme for people leaving CRT services. Stage 4 involved qualitative evaluation of preliminary feasibility testing of the intervention, leading to further refinement of the intervention content and procedures. Finally, stage 5 consisted of further qualitative evaluation of the programme during its testing in a pilot randomised controlled trial. Figure 1 describes the iterative process of intervention development and evaluation undertaken.

### Stage 1: evidence review

Two systematic reviews and meta-analysis of interventions were completed by the research group in collaboration with the national collaborating centre for mental health: (a) self-management interventions for serious mental illness [20] and (b) peer support for serious mental illness [20, 27]. Both reviews informed the updated NICE Schizophrenia guidelines [20], where review methods are reported.

Methods for the qualitative components of the intervention development process (stages 2–5) are described below. The setting, participants and measures for each stage are described separately; then procedures and an analysis strategy which were common to stages 2–5 are reported.

### Stage 2: developing theory—CRT service user interviews

Service users were recruited in 2011/12 from CRTs in 10 NHS trusts in England, representing a range of inner city, suburban and more rural areas. We sought four participants from each Trust, who had been discharged from participating CRT services within the last 3 months. Purposive sampling was used to ensure a range of demographic characteristics among the sample, and to include both first-time and repeat users of the CRT. Semi-structured interviews first explored participants’ experience of CRT services and views on CRT care. These findings have been reported elsewhere [28]. Interviews also asked specifically about participants’ views on the support needed following discharge from a CRT service, their views on this support being peer-provided, what content they would see as useful to incorporate into the programme, and how this might be structured and delivered. Responses to these questions were analysed and are reported in this paper. Most (79%) of interviews were conducted by service user-researchers from the study team who had received specialist training in qualitative interviewing and were offered support and supervision by study researchers (other interviews were conducted by non-peer study researchers).

### Initial intervention selection

Findings from the evidence review and CRT service user interviews (stages 1 and 2) informed the initial choice of intervention. CORE study expert reference groups of CRT clinicians (n = 8) and CRT service users and carers (n = 20) advised on potentially suitable resources for use or adaptation in the CORE study trial, guided by members’ clinical or personal experience of self-management resources used in NHS mental health settings.

### Stage 3: modelling—stakeholder focus groups

Participants were recruited in 2011/12 from 5 NHS trusts in London, South East and South West England. Twelve focus groups were conducted with CRT staff and managers (n = 5), CRT service users (n = 5) and family members/carers of CRT service users (n = 2). Between 6 and 10 participants were sought for each group, purposively sampled to reflect a range of demographic characteristics; extent of previous CRT use (service users and carers); or professional background (CRT staff). A 15-min PowerPoint presentation on the aims of the CORE trial and the proposed intervention was presented to participants. Focus groups were then co-facilitated by two researchers using a topic guide; service user and carer focus groups were led by a peer researcher. Discussion focussed on the acceptability of a peer supported self-management programme, views about how the proposed intervention would work best in a CRT context, how, when and by whom the intervention should be delivered, and the structure and integration of the intervention in existing care pathways.

### Intervention adaptation

Adaptations to the initially selected intervention were then informed by the findings from stage 3 focus groups and further discussion with the study team and expert reference groups.

### Stage 4: feasibility—qualitative evaluation

Preliminary testing of the intervention was conducted with service-users (n = 11) recruited from one North London CRT service in 2012. Following completion of the intervention, feedback was obtained through a focus group with the peer support workers (n = 4) and individual interviews with the service user participants (n = 9). Interviews explored experiences of the intervention and how it might be improved. PSW focus group topics included views on: training and supervision for the role; content, structure and delivery of 1:1 sessions; the self-management workbook; and the perceived impact of the intervention on providers and participants.

### Intervention refinement

The stage 4 evaluation informed further refinements to the study intervention and operating procedures, following further discussions with expert reference groups and the study team.

### Stage 5: piloting—qualitative evaluation

A Pilot randomised controlled trial of the study intervention was completed in 2013. CRT service users randomised to the treatment group were contacted at the end of trial intervention 4-month follow-up point and invited to complete a qualitative interview with a study researcher. Interviews explored participants’ engagement with and experience of the intervention, which elements they found most or least helpful, and any suggestions for how the intervention might be improved.

### Common features of qualitative phases of the study

For all four qualitative components (stages 2–5), interview and focus group topic guides were developed with involvement from the study service user and carer expert reference groups and CRT clinicians within the study team. At all stages, participants were recruited via involved CRT services, with clinical staff from within CRT teams making the first approach to service user and carer participants. All participants were provided with a written information sheet about the study and met a researcher to discuss participation and provide written, informed consent. Ethical approval was obtained for each phase of the study from the London Camden and Islington Research Ethics Committee (Ref Numbers: 10/H0722/84, 11/L0/2010, 12/LO/0988). In line with the study’s ethical approvals, data about the demographic characteristics of participants was collected, but no information was collected about the characteristics of those who declined to participate.

Qualitative data from all stages of the intervention evaluation process were recorded and transcribed verbatim. Data from each stage was analysed separately using thematic analysis [29] within NVivo software. The analytic focus was on features of the data which addressed views and experiences of the intervention. Our strategy combined inductive and deductive approaches throughout, allowing exploration of initial research questions relevant to each stage of the intervention development, as well as emergent themes and issues that characterised respondents’ own experiences of receiving or delivering the intervention. In order to enhance validity, a collaborative approach was adopted: The principal analyst (AM) was supported by a small team of researchers, including those with service user, carer and clinician perspectives. These researchers all contributed to reading transcripts and developing of an initial coding frame, through discussion. Each transcript was then coded in NVivo by one researcher, with reference to the initial coding frame. The addition of new themes, or the merging of themes or sub-themes with considerable overlap, was then agreed through discussion with the lead researcher. Each individual transcript was then read by a second researcher, and any further suggested revisions to the coding frame or disagreements about the coding of specific text were resolved through regular discussions among all coders. This collaborative process encouraged collective reflexivity about interpretations of the data in relation to the perspectives and preconceptions of the researchers. As such, it enhanced the validity or trustworthiness of findings [30]. Member checking was not conducted.

Due to limitations of space, and the aims of this study, we focus in this paper on features of the qualitative data that are most relevant to informing intervention design and delivery.

## Results

Table 1 provides an overview of the intervention development process undertaken in this study, summarising for each stage: the methods used, the nature of key findings obtained, and how these informed the development of the intervention. The results from each stage of the study follow.

**Table 1:** Overview of the development process for a peer-facilitated self-management intervention

	Intervention development			Feasibility testing and piloting	
	Stage 1	Stage 2	Stage 3	Stage 4	Stage 5
Method	Evidence reviews: peer support and self-management literature	Developing theory: interviews with CRT service users (n = 41)	Modelling: stakeholder focus groups (n = 12)	Feasibility testing evaluation: interviews with participants (n = 9) and PSW focus group	Piloting evaluation: interviews with participant (n = 18) and PSW focus group
Key findings	Promising evidence for self-management interventions for SMI—which can be peer-delivered	A need for additional support following CRT discharge was identified Peer-provided support was acceptable to most CRT service users A focus on help with wellness planning and relapse prevention was valued	Peer-provided, post-CRT support was endorsed as different but complementary to routine clinical care Time-limited, 1:1 support with a focus on self-management and personal recovery was endorsed Considerations for PSW training and support and linking with clinical teams were raised	The intervention was well-received Advice from all was to use the structured workbook flexibly, in an individualised way, to meet participants’ needs and preferences	No further changes to intervention content or structure were recommended Challenges were identified for PSWs of integration with CRTs, maintaining role clarity, and managing boundaries and endings
Intervention development decisions	Decision in principle to provide a peer-provided, self-management programme Provisional selection of a structured self-management resource		Endorsement of an adapted recovery plan self-management resource Specifications for programme delivery were developed	Adaptations to the recovery plan workbook to increase opportunities for personalised use Systems to link PSWs to the CRT team were refined	Arrangements for PSWs’ training and supervision, and communication with CRT teams were refined

### Stage 1: evidence review

The systematic review of self-management interventions confirmed the potential value of self-management programmes to support recovery from mental health crisis: it found evidence of short and long term positive impacts of self-management programmes on symptoms and recovery-focused outcomes [20]. Interventions in reviewed trials typically offered guided self-management, with support offered by a clinician or a peer worker. Although there was no clear typology for self-management programmes, effective interventions usually covered at least three of the following: recovery principles, addressing stigma or exploring personal meaning; psycho-education about illness or treatment; relapse prevention (identifying and monitoring early warning signs and triggers; developing stress management and coping strategies); crisis planning or advanced directives; signposting and accessing resources; medication (e.g. understanding medication and side effects, planning, management); establishing personal goals/plans and/or mental wellness maintenance strategies.

The systematic review of peer support interventions for people with severe mental illness found some indications of positive impact on recovery-focused outcomes such as hope and empowerment, although little evidence of the effectiveness of peer-provided support for clinical outcomes [27]. The content of interventions in included studies varied substantially: there was no evidence about which models of peer-provided support were most effective. No studies were conducted in CRT settings or following CRT care. A scoping review of qualitative literature found consistent positive appraisals of peer support programmes, as being highly valued by service users and able to provide something different from clinician-provided care and supportive of personal recovery [31].

From these reviews we concluded that: (1) a programme including structured self-management support and a recovery-focus has potential to help people following a mental health crisis. (2) a peer-supported, self-management programme following a mental health crisis/CRT discharge was not contra-indicated by existing evidence, and that a trial of such a programme in the UK would add to current knowledge;

### Stage 2: CRT service user interviews

Interviews were completed with 42 CRT service users, but one interview was not successfully audio-recorded, so was excluded from analysis. Participants’ characteristics are reported in the data supplement (Additional file 1: Table DS1): participants reflected a range of demographic and service use characteristics.

Data were coded in five main categories from analysis: (1) acceptability; (2) what the intervention should contain; (3) when the intervention should take place; (4) who should be involved in delivery; and (5) where and how the intervention should be delivered. Results for each theme are provided in more detail in Additional file 2: DS4. The main findings from stage 2, with implications for development of the intervention are summarised below.

A large majority of participants felt the idea of additional support with recovery following CRT care was positive. Participants’ expressed needs were in line with the self-management approach and that peer support was a generally acceptable form of programme provision. An exemplar quote from one participant was:

Whenever I’ve been an inpatient before, I’ve always found it very beneficial to be able to relate to other people that have been in similar situations; that they are experiencing the same things as yourself. (SU13)

Overall, there were positive comments from 31 of the participants, seven participants expressed neutral comments and there were negative comments from six of the participants. The small number of negative views mostly related to the participants wanting to move on with their lives away from a mental health setting as quickly as possible.

No, I don’t really think I need it; at the moment I don’t feel I need it… I think if I had more people still seeing me I think it’d make me feel as if I’m still not as well as I think I am. (SU01)

While a minority of participants preferred a befriending type arrangement with no prescribed or specific self-management focus, most advocated a programme offering a combination of mutual, practical, social and mental health support. Some favoured a structured form of delivery, while others felt a degree of flexibility was required.

I don’t suppose anyone’s going to feel exactly the same every week. Sometimes you might really like to say, I don’t want to talk about how I feel, can we just look at the plan? Or, I just want to talk about how I feel, I don’t want to look at the plan, that kind of thing. To be flexible I think is probably the most important, but a bit of both. (SU28)

Preferences for delivery of the intervention were relatively equally distributed across one on one support, group based support and internet support, but additional barriers for some to group based and internet interventions were raised: feeling uncomfortable in a group setting, and finding a time convenient to all, or not having computer skills or equipment. The programme was recommended as complementary and additional to professional services. A majority of participants expressed the view that peer support workers’ lived experience could offer a unique and empathic perspective to support. A small number of participants were in favour of health professionals’ support rather than PSWs. One participant highlighted that it was important for the PSW to be well trained and be able to handle complex situations.

I think if it’s just somebody that’s had mental health problems with a little bit of extra training but has not had a lot of training or experience and is well suited, then I think maybe not because of the level of risk involved. (SU25)

Table 2 summarises stage 2 CRT service users’ and stage 3 focus group stakeholder participants’ suggestions for the content of a post-CRT supportive intervention. In the stage 2 CRT service user interviews, all components of self-management programmes identified in the stage 1 evidence review were supported to some degree by participants, except support with medication: practical linking/signposting support and wellness planning were proposed most frequently by CRT service users.

**Table 2:** Development of the content of the peer-facilitated self-management intervention (stages 1–3)

Concepts	Definition	Stage 1: evidence review findings	Stage 2: CRT service user interviews	Stage 3: focus groups	Illustrative quotes
Peer support	Support from a worker with a lived experience of mental illness	Interventions using peer support have currently inconclusive evidence of efficacy; however, they are widely used	Mutual support from a peer (27 respondents) The PSW actively get to know the peer (5 respondents)	Befriending (7 sources) Role-Modelling (8 sources) Lived experience support beneficial (5 sources)	*I think it’s great in a very different, unique kind of way. However experienced and qualified the staff are I don’t think anything is as supportive as somebody who’s been through the same services as a service user that you have. I think that’s a really unique opportunity and potentially really helpful. (stage 2 interview SU28)*
Recovery	Recovery principles, e.g. addressing stigma or exploring personal meaning	A concept in 12 of 19 reviewed mental health self-management programmes	Recovery (9 respondents)	Recovery (6 source) De-stigmatising (3 sources)	*…generally things around recovery where you can gain knowledge and understanding about how to self*-*manage a condition to prevent the risk of relapse (stage 2 interview SU26)* *And I think as much as we’re trying to pull away from the medical model and embrace the recovery model (stage 3 Staff Focus Group 4)*
Psycho-education	Such as illness specific education, treatment information	A concept in all 19 reviewed mental health self-management programmes	Psycho-education (12 respondents)	–	*I had so much information running through my head and I couldn’t understand that information at all, and I think if somebody came to tell me and explain it to me that this is what happened to me, and it would be maybe completely different from me but it would be… they would be telling me that I had psychosis and these are the signs of psychosis, I would find it really helpful (stage 2 interview SU21)*
Relapse prevention	Identifying signs of relapse and planning coping strategies	A concept in all 19 reviewed mental health self-management programmes	Relapse prevention (11 respondents)	Relapse prevention (7 sources) Strategies to keeping well (3 sources)	*If a peer support worker was to identify together with whoever was using the service what their warning signs were and to make sure they’re aware of their own because there’s a tendency to be, like, these are people’s general warning signs and in fact they can be really different for different people and to personalise it in that way because that’s, I think, what really makes a difference—when you feel like it’s relevant to you. (stage 3 Service User Focus Group 1)*
Crisis planning	Planning service response in the event of future crisis	A concept in 11 of 19 reviewed mental health self-management programmes	Crisis planning (5 respondents)	Crisis planning (7 sources)	*If it’s following the WRAP (TM) programme then it’s definitely good (stage 3 Staff Focus Group 4)*
Signposting	Aiding access to various services by linking, referring or signposting	A concept in 17 of 19 reviewed mental health self-management programmes	Identifying supporters (4 respondents) Signposting to resources (4 respondents) Accessing community and social engagement (15 respondents) Practical support to link to resources (15 respondents)	Signposting (7 sources) Practical support (4 sources) Social engagement (10 sources)	*And maybe the peer support should have a little more information about other services that are there, just do the sign posting, because with some clients inevitably the first thing they’re going to talk about always is those issues (stage 3 Staff Focus Group 2)*
Medication education or management	Aiding the participant to understand their medication and medication side effects. This may include planning psycho-education or specific medication management	A concept in 17 of 19 reviewed mental health self-management programmes	–	Concerns around including medication management for this programme (3 sources)	*I have seen people discourage people from taking medication when it’s been prescribed for them, oh, you don’t want that, and it’s an issue to have in mind because, as I say, there’re all kinds of opinions about medication* *(Staff Focus Group 4)*
Goals and wellness planning	Establishing personal goals/plans and/or mental wellness maintenance strategies	A concept in 13 of 19 reviewed mental health self-management programmes	Goal setting (7 respondents) Wellness planning (14 respondents) Focusing on the future (6 respondents)	Future plans and goals (4 sources)	*Somebody who would come in after the home treatment team have seen you, just to help you focus on the future, and look forward. Yes, don’t focus on the negative, rather the positive. Yes, and just… let them, you know, let you know what’s out there for you, as a person, just things maybe you can do or not do whatever, you know? Setting goals really and very small steps.(stage 3 Service User Focus Group 3)*

### Initial intervention choice

The CORE service user, carer and clinician expert reference groups were presented with findings from the stage 1 evidence review and stage 2 interviews and consulted about possible interventions. The personal recovery plan [32] was identified through the clinicians’ reference group and approved by the study team and all advisory groups as a suitable resource template for peer-provided, self-management support for people following a mental health crisis. Developed by Rachel Perkins and Julie Repper in co-production with mental health service users, the personal recovery plan [32] was designed to be used as a self-management resource to support mental health recovery, which could also feed into discussions with mental health staff where relevant. Four reasons for its suitability for our study were: (1) it covers self-management themes identified as helpful in our evidence review and service user consultation (relapse prevention planning, goal-setting and wellness planning); (2) it specifically covers recovery from a mental health crisis, so is directly relevant for a CRT client group; (3) it was originally co-produced with people with lived experience expertise, and has a strong recovery orientation, so is suitable for delivery by peer support workers; and (4) it has been used with mental health client groups within the NHS, so there was promise of its feasibility and acceptability in our study.

Adaptations to this self-management resource and specification of the study intervention were then informed by three further phases of data collection (stages 3–5).

### Stage 3: stakeholder focus groups

Twelve stakeholder focus groups with service users (n = 20), clinicians (n = 41) and carers (n = 12) from five NHS Trusts. Participants’ characteristics are reported in Additional file 1: DS2. Seven main themes were identified from these focus groups, relating to the content of the intervention and requirements for different stages of its delivery, the qualities required from PSWs and needs for supervision and training. Results for each theme are described in Additional file 3: DS5. The main findings with implications for the development of the intervention are summarised below. Most participants welcomed the proposed peer support self-management programme, for example:

A brilliant idea provided it doesn’t take the place of what’s already in place (Carers Focus Group 1).

I think we do see some people that could benefit from that little bit more after we’ve sort of, after our treatment episodes have finished, but they don’t fit the CMHT criteria or anything else. And then at some point they usually do end up coming back to us because there isn’t anything else for them and then maybe something like this could be good for them. (Clinicians Focus Group 2).

The peer led nature of the support and the proposed timing of the intervention (i.e. the support being provided directly after a period of crisis care) were endorsed. Participants suggested the programme could promote shared knowledge and understanding and that peer support workers could act as role models.

A lot of people really are inspired and feel empowered by someone who has recovered, or has gone through the same processes as them, and it’s like a model for them to look up to and say, well if you’ve done it, I can do it too. (Carers Focus Group 2)

… mental health professionals, most of them are just reading it out of books and stuff they’ve learnt. They’ve not actually experienced it so they don’t know it for themselves, so it would be good to talk to someone that’s been through it, as well, and see how they’ve found ways of getting help and stuff like that (Service User Focus Group 3)

And, maybe that person who’s looking after, not looking after you, but supporting you, can say, yes, I know what you’re talking about, you know? It’s just been helpful for them, maybe someone to identify what you’re saying, and have some more greater understanding than maybe a lay person will. (Service Users Focus Group 5)

The programme was seen as de-stigmatising, bridging a gap in services and promoting continuity of care.

… at some point they [CRT service users] usually do end up coming back to us because there isn’t anything else for them and then maybe something like this [the program] could be good for them. (CRT Staff Focus Group 1)

More negative views of the intervention were less common and mostly raised by carers and clinicians. These concerns related to the peer led intervention potentially taking the place of professional services, that there may not be sufficient training and support systems in place and that the PSWs may become stressed or unwell. This highlighted the need for adequate training standards, protocols, supervision and recruitment procedures.

I think the professionals are the ones that should be doing that job, not the peers. They should all be in a group, because they’re not qualified. They know how they feel, but… (Stage 3 Carers Focus Group 2)

… making sure that person has got boundaries with you, and things like that? Which hopefully would have been part of their training. (Stage 3 Clinician Focus Group 3)

A combination of structured support, and retaining a flexible and individualised approach was recommended for intervention delivery. As in the initial service user consultation, while there was some support for a group intervention, 1:1 support was advocated by more participants. It was seen as an easier way to tailor support to each individual, and helpfully similar in style to the support participants received from the CRT. The potential for some people to feel uncomfortable in group settings was raised.

One to one would be much better than a group, because you’ll get more feedback from him or her individually, rather than you would do in a group. (Service User Focus Group 2)

I find myself being less open or less trusting [in a group] than if it’s just one on one (Service User Focus Group 1)

Self-management concepts (relapse prevention and crisis planning, signposting to services, goal setting, confidence building, practical support) and the relational aspects of peer support (social engagement, providing company, befriending) were both endorsed.

Some reservations about unqualified peer workers supporting service users with medication management were expressed by staff focus group participants. Illustrative quotes of recommendations for the content of the programme from focus group participants are provided in Table 1. Fuller results from the stage 3 stakeholder focus groups are provided in the data supplement (Additional file 3: DS5).

### Intervention adaptation

Following the stage 3 focus groups, adaptations to the personal recovery plan selected self-management resource [32] were made in consultation with the service user and carer study expert reference groups, newly recruited peer support workers, and members of the research team including senior CRT clinicians.

#### Programme/intervention content and presentation

The recovery plan was abbreviated to fit within a ten-session programme of support. A section on specifying future service response in a crisis was omitted, acknowledging that some participants in the CORE trial would, following CRT discharge, not have ongoing support from mental health services with whom to confirm a crisis plan. A section on “moving on after a crisis” was brought to the front of the plan, as participants in the CORE trial would all have recently experienced mental health crisis and CRT support. The resulting adapted resource was titled ‘My personal recovery plan’, and was designed with images of green budding plants to represent personal growth. The plan had four main sections for completion by the person using the workbook: ‘moving on again after a crisis’; ‘keeping well’; ‘managing ups and downs’ (relapse prevention); and ‘goals and dreams’. At the back of the workbook was accompanying guidance titled ‘making a personal recovery plan’. The main focus of these sections was to help people to identify strategies to monitor their own warning signs, develop their own coping strategies and identify sources of help.

#### Programme delivery specifications

Plans for a ten-session intervention of 1:1 support, beginning at the point of discharge from a CRT, were confirmed. It was agreed that the peer support worker would complete their support within a 3 month period, to keep the intervention’s focus as short-term bridging support following a crisis, rather than longer term continuing care. A 4-day training programme for the peer support workers providing the intervention was developed in collaboration with an experienced peer training team from the Institute of Mental Health, Nottingham. This adapted and abbreviated the accredited course ‘‘an introduction to peer support’’ [33]. It was supplemented with relevant generic NHS training (including safeguarding and personal safety) and a 1-day induction to relevant local policies and procedures. Arrangements for regular group supervision for peer support workers were specified. Table 3 summarises the adaptations and specifications to the intervention made following stage 3 focus groups and stage 4 feasibility testing. In the feasibility testing, peer support workers’ training was provided by the Nottingham institute for mental health training team. Peers were recruited from the CORE study service user expert reference group and supervised by a research psychologist from the study team (AM).

**Table 3:** Summary of programme specifications and recovery plan adaptations resulting from modelling and feasibility testing (Stages 3 and 4)

Area	Decision	Rationale
Self-management resource	Personal Recovery Plan (Repper and Perkins 2008) selected	(i) Covers elements of effective self-management programmes (literature reviews) (ii) Specific focus on recovery mental health crisis (iii) Co-produced with service users—suitable for delivery by a PSW (iv) Has been tested in NHS settings
	Programme abbreviated	To fit within a 10-week programme of support
	Section on developing a crisis plan omitted	Acknowledging that not all participants in the CORE trial will have ongoing support from mental health services in place, or meetings with the PSW and mental health services may be difficult, so agreeing service responses in a crisis may not be possible
	Section on “moving on after a crisis” brought to the front of the plan	All participants in the CORE trial will have experienced a recent mental health crisis, so this is likely to be an immediate concern
	“Recovery means” page left for individual to complete	Feedback from PSWs and service user reference group: more individualised to encourage people to provide their own definition of personal recovery
	Redesign with green leaf motif	Feedback from service user reference group: calming and symbolic of growth and renewal
	More white space incorporated into the plan	To allow free text writing, drawing or adding photos etc. for those with literacy difficulties or who prefer a less structured approach
Structure of the programme	10 session programme	To maintain the focus of the programme on brief, bridging support with recovery following a mental health crisis (rather than longer term support)
	Programme to begin immediately following CRT discharge, and be completed within 3 months	
	1:1 support	Likely to be acceptable and feasible for more participants than a group programme
Values of the programme	Recovery focused: promoting hope and valuing participants’ strengths	Consistent feedback from service users and other stakeholders about what help is wanted and needed following CRT support and advocated by peer support literature and training programmes
	Peer-delivered: appropriate self-disclosure and story-sharing is encouraged; modelling recovery and coping	
	Person-centred: the recovery plan to be used flexibly, in an individualised way with each participant	
	Complementary to mental health services care (integrated within CRT services, but offering additional, distinct support)	
Peer Support Worker (PSW) recruitment	Essential requirements for PSW roles defined as: having lived experience of mental health problems, previous experience in a support role, good interpersonal and support skills, has developed a personal recovery plan/relapse prevention plan, has a recovery-oriented approach, ability to problem solve and work collaboratively with mental health staff and service users	Lived experience and having own recovery plan to ensure positive story-sharing and modelling of recovery strategies is possible; no requirement for clinical qualifications and broad range of previous experience in a support role accepted, recognising the non-clinical recovery focus of this role
	Open market NHS employment, competitive recruitment	To recognise the demands of the role and support integration with CRT teams
PSW training and supervision	Adapted Nottingham IMH training covering: the meaning of peer support, self-management and recovery; core skills: listening, valuing diversity, strengths-based; peer support skills including story sharing; boundaries and disclosure; referring and linking in; working with distress and addressing safety concerns	Drawing on an established, accredited training course used to support peer workers in using the personal recovery plan
	Additional NHS Trust training and induction (including safeguarding and personal safety training; orientation to NHS policies and procedures)	To support safe working and integration with participating NHS mental health services
	Regular group supervision delivered by participating NHS Trusts	As above; Group supervision was chosen to maximise the PSWs’ opportunities to learn from and support each other
	Access to 1:1 supervision too	Reinforced as important by PSWs following preliminary testing. Access to additional support from an experienced PSW to ensure PSWs’ distinct role is retained and supported
	PSW access to immediate support and advice from CRT staff following meetings	
	Access to support from experienced peer support worker	

### Stage 4: feasibility testing

For the preliminary testing of the intervention in 2012, 11 participants recently discharged from CRTs were recruited. Nine agreed to take part in a qualitative interview at the end of the intervention. The demographic characteristics of service user participants from stages 4 and 5 are presented in Additional file 1: DS3. Feedback from participants in these stage 4 interviews was categorised in nine main themes, covering barriers to engagement, experience of the programme and the PSW, and their helpful and unhelpful aspects. Results for each theme are provided in Additional file 4: DS6. Findings with most relevance to the development of the intervention are summarised below. The four peer support workers who delivered the intervention participated in a focus group. Four main themes were derived from this focus group, relating to perceived benefits of and challenges with the programme, the experience of supporting participants, and issues around information sharing with involved clinical teams. Results for each theme are provided in Additional file 5: DS7. Key findings from this focus group with relevance to the development of the intervention are included in the summary below.

Eight participants commented that they had an overall positive experience of the manner of delivery and content of support. One had a more negative experience and felt he did not gain anything from the intervention and support.

And I think these are very, very helpful, because it makes you realise what’s achievable. For me, it makes you realise, you know, that to me recovery means being in the driver’s seat. I think it’s fantastic. (PP03)

… it weren’t unhelpful, it just weren’t helpful, if you know what I mean. It weren’t like it was bad; there was just no point to it. (PP07)

Both participants and PSWs commented that although some participants found the self-management workbook very helpful, others did not like its structured nature. Poor literacy was a barrier to using the workbook for two participants. Support from the peer support worker, discussion and unstructured written work or drawings, was valued as a means to address these challenges. PSWs and participants advocated providing more free space for drawings and unstructured work. From both the participant and the PSW perspective, flexibility in delivering the intervention was identified as crucial. Where experienced as helpful, the workbook was viewed as a tool to refer to in times of stress or deteriorating health in future.

I wrote it down in words. I’m better off writing down, better than saying it in words, you know. I mean, if you write it down you got… you can study it more, you know, you can read it and digest it. But if you say it, it tends to go out the memory, doesn’t it, quick. (PP08)

The workbook could also act as a means to focus on future ambitions, or, for a few participants, to share these with family or friends.

And I showed my dad this [the workbook] and he was delighted. He was really sweet. And he said goals and dreams, that’s what you’ve got to focus on. (PP03)

Some participants reported the relationship between peer worker and service user was more useful than the structured self-management workbook, and relational aspects of the intervention impacted greatly on experience. PSWs were valued by participants both for the warm relationship per se, and for the practical information and support they provided.

And while everyone sort of was telling me that they would help, but no one really did anything, while she actually did things. And I really, really appreciate this (PP01)

The participant who held a more negative view on the experience suggested that matching the peer workers to participants based on common interests may be useful. Commonly from both the participant and the PSW perspective, where positive relationships developed, a sense of loss occurred when the intervention ended.

It’s quite painful I think to go into someone’s life and, like, fulfil that role; to be the first person to have that, kind of, caring, supportive relationship with someone and not being able to replicate that anywhere and then leave that person. That’s quite painful. (PSW1)

Strategies proposed to mitigate this loss were: (1) a clear message of the intervention timeframes from the outset, (2) a “tapered” approach to ending the support (i.e. planned decrease in frequency of sessions before the last session to meet service users’ needs), (3) a focus on linking with services to establish additional supports, (4) planning and goal setting for after the intervention ended, and (5) marking the end of the intervention with positive activities (such as a small informal celebration and revisiting the individual’s goals and achievements).

PSWs reported finding the extent and scope of difficulties faced by participants in their daily lives a challenge to supporting people effectively. Navigating the boundaries of the PSW role and managing relationships with participants which could become quite intense were also recognised as challenges by PSWs. Supervision and access to immediate debrief from supervisors or others following difficult sessions were advocated. Peer support among the PSW team was valued: PSW-only supervision sessions and informal meetings were identified as facilitating this.

I think they [supervision sessions] were just immensely important. I don’t think we could have done the work we’ve done without them, to be honest, without getting together like that and sharing our experiences and knowing that somebody else who’s doing, who’s a peer supporter, has experienced the same problems and the same emotional things that you’ve experienced. I think that was essential. (PSW 3)

### Intervention refinement

Minor changes to the language in the plan were informed by the findings from stage 4 feasibility testing and further discussion with the study service user and carer expert reference groups. Replacing a definition of recovery in the original plan, space was created in the study version for participants to record their personal meaning of recovery, following discussion with their peer support worker. More blank space was included throughout the plan, to allow participants space to include information, potentially using a range of media, relevant to their personal recovery plans. In the stage 4 feasibility testing, PSWs identified strengthening the PSW link with CRT team as important. Processes to embed the PSW teams within the CRT service were planned for the next piloting stage (stage 5), such as PSWs meeting the whole CRT team at the start of the intervention, and having their photos and an explanation of their role prominently in the service base, and clarifying a streamlined referral pathway back into CRT for the participant if they became unwell. The resulting adaptations and specifications of the study intervention following stage 3 and 4 development are summarised in Table 2.

### Stage 5: piloting

Forty participants were recruited to a pilot randomised controlled trial of the study intervention in 2013. Of 21 randomised to receive the peer-supported self-management intervention, 18 participated in a qualitative interview following the intervention. Their characteristics are reported in Additional file 1: DS3. Feedback from participants was categorised into eight main themes, relating to helpful and unhelpful aspects of the programme overall and its structure, the workbook, and the relationship with the PSW. Results for each theme are described in detail in Additional file 6: DS8. Four of five PSWs who delivered the intervention in the pilot trial participated in a focus group. Their feedback was categorised into: comments on the workbook; and overarching themes (including views on supervision and support; the PSW role boundaries; and ending of sessions). Findings from this focus group are reported in detail for each theme in Additional file 7: DS9. Findings from these interviews and from the focus group with most relevance to intervention development are summarised below.

Overall, feedback from PSWs and participants in the pilot trial was similar to that from preliminary testing. Although mixed views were expressed by participants on how much the self-management resource was used or valued, no significant new changes were recommended. The recovery plan workbook was described by the PSWs as a helpful framework for sessions, providing that there was flexibility in its delivery for each service users as they moved through the intervention. Participants identified a range of barriers to utilising the support on offer. Lack of stable accommodation or the recurrence of illness made arranging sessions difficult for two participants. Another felt he had too much else going on to prioritise meetings with the PSW. One participant was disappointed by his PSW’s inability to help with a housing problem, while two identified a difficult relationship per se with their PSW as a problem:

I don’t like people telling me what… I’ll do it this week, I’ll do it that week. And you know, I’ve always been very independent and stuff. And I just find it a bit patronizing. So I’d rather, you know, do it my own, kind of, time, and frame… (MP17)

Twelve out of the eighteen interviewed participants expressed a clearly positive view of the programme however: both the empowering relationship with the PSW and the self-management guidance offered by the workbook were identified as helpful.

As a patient, sometimes you feel, like with anything, that, you know, the doctor is the expert and you are the person receiving it. And you don’t necessarily have a voice. And I suppose [seeing a PSW] did let me think, well, hang on, I can voice my worries … (MP15)

Like, it has opened my eyes to a few things, you know, just from doing the book. I write down a lot more stuff now, just from doing this, you know. It’s little things but personally I think it’s the little things that matter in life. (MP22)

PSWs ‘feedback focused on the organisation and support structures of their role, with the need for role clarity, boundaries and managing endings again emphasised. PSWs reported that the location of support and supervision within the participating CRT team in the pilot trial brought advantages and disadvantages. Integration with the CRT team and access to immediate support or advice following a difficult meeting were valued by PSWs, who disliked the occasions when calls to the CRT were met by an answerphone. While integration with the CRT was perceived as improved from the previous, feasibility testing stage, one PSW still reported substantial need for further integration.

No one [at the CRT] says hello. I don’t know any of their names, like, and I think just some things like that could have just been done a bit better (PSW1)

Some concerns were raised that supervision by CRT clinical staff risked eroding the unique, non-clinical role of the PSWs: contact with and access to additional support from an experienced peer support worker were advocated.

The workbook was valued by PSWs’ as a means of structuring sessions and to guide the content of conversations with participants, although it was a common view that not all participants might wish to complete the whole written plan.

It’s a framework, which some people I think do find it useful, you know, undoubtedly to fill something in, you know. But, you know, it’s almost like, it’s kind of like a framework to hang discussion on, to always kind of have in the back of your mind that it’s kind of about those kind of issues. (PSW4)

While implications for PSWs’ training and supervision from pilot trial qualitative evaluation were fed back to services and informed ongoing training and supervision, no additional changes to the content of the recovery plan resource or the structure of the intervention were made following piloting: the intervention was delivered in the main randomised controlled trial as in the pilot. In the pilot trial, and the main randomised controlled trial, peer support workers were recruited through a competitive NHS recruitment process; training was provided by clinicians and peer support workers from the study team (who had attended the training programme in the preliminary phase), and supervision was provided by experienced clinicians within participating NHS trusts.

## Discussion

The study comprised five stages of an intervention development process which included: (1) review of existing relevant evidence; (2) Interviews with service user stakeholders exploring the need for, acceptability of, and optimum type of support following CRT discharge; (3) focus groups with CRT stakeholders regarding views in principle about a proposed peer-provided, self-management intervention; (4 and 5) feedback from recipients and providers of the study intervention about its acceptability and usefulness in feasibility testing and piloting. At each stage, an iterative process of using feedback to evaluate and improve design and delivery of the intervention for a fully defined large scale RCT [26] was used in accordance with expert guidance on developing and evaluating complex interventions [25].

### Strengths and limitations

Initial evidence review for this study was thorough, involving two systematic reviews of relevant interventions. Interviews with CRT service users about priorities for support after CRT discharge, and stakeholder focus groups about a proposed intervention, involved substantial numbers of participants across a range of CRT services and different geographical settings, which increases confidence in the generalisability of findings. Multiple interviewers were used to conduct the forty-one initial interviews with CRT service users and the twelve CRT stakeholder focus groups: this may have reduced the consistency with which interviews were administered, although the use of peer-interviewers for service user and carer interviews may have helped participants to feel comfortable and speak frankly [34].

The scope of this paper is to describe the development of a peer-supported, self-management programme for people following a mental health crisis. While qualitative feedback from participants provides some evidence regarding the acceptability of the intervention and its possible impact on participants’ experience, this paper provides no evaluation of the effectiveness of the intervention, which will be evaluated across a range of outcomes in a randomised controlled trial, to be reported separately. Preliminary testing and piloting of the study intervention was limited to a single NHS Trust, and to participants willing to take part in a research trial: the generalisability of these qualitative findings to clinical populations overall in CRTs cannot be assured. Further testing of potential mechanisms of effect is required to develop a convincing change model for this complex intervention (see research implications below). Finally, due to limitations of space, and the aims of this study, we focus in this paper on features of the qualitative data that are most relevant to informing intervention design and delivery. The results of the qualitative evaluations in stages of intervention development which are provided in this paper are summaries only: fuller presentation of results is provided in the data supplement (Additional files 2–7: DS4–DS9).

### Implications for research

Three implications for research may be derived from our study. First, the evidence reviews conducted for this study confirmed the need for more evidence regarding supported, structured self-management interventions in a UK crisis context, and for clearly-defined PSW-provided interventions in all contexts [20, 27]. The randomised controlled trial of the CORE Study peer-provided, self-management intervention [26], for which this paper reports the development work, will help address this gap in knowledge.

Second, this paper illustrates how the phases of the MRC complex interventions framework [25] can be applied in practice to develop an intervention ready for trialling, by systematically incorporating the evidence base with current viewpoints, whilst iteratively testing and refining the intervention design as it develops [35]. The evidence review stage established the underlying theory of the self-management intervention informed by empirical and theoretical evidence and also allowed us to identify existing models for adaptation and implementation in our study. The second stage of interviews with CRT service users established the a priori acceptability of the proposed intervention for the target group and proposed mechanisms through which it might help recovery for service users after a period of crisis care. The third modelling stage gauged the feasibility of the intervention and informed necessary adaptations and planning for organisational support, through focus groups with wider stakeholder groups including service users, carers and CRT staff. Delivery of the intervention in a fourth, feasibility testing stage allowed further modelling and the incorporation of feedback from intervention recipients and providers into the intervention’s final adaptation and implementation planning, which was tested and qualitatively evaluated in a fifth piloting stage for a definitive randomised controlled trial [26]. The full-scale trial can thus evaluate a clearly-specified, coherent and road-tested intervention of support to meet a defined clinical need: this will maximise the scientific value of the trial.

Third, the intervention described in this study is complex and multi-faceted. The self-management [12] and peer support [36] literatures both propose change models, generating a number of potential mechanisms of effect for our intervention. There is thus a need to add to the work undertaken in this paper, of modelling the intervention and exploring how it was received, with a process evaluation which measures how the intervention is delivered and explores the relationship between process and outcomes on a larger scale. The forthcoming randomised controlled trial of the peer-provided, self-management intervention described in this paper will provide this [26]: a process evaluation planned as part of the trial analysis plan will explore how the following variables relate to any positive outcomes from the trial: participant-rated therapeutic alliance with the PSW, and the PSW’s recovery orientation; the degree of match between PSW and participant (on demographic characteristics, diagnosis and service use); and participant-reported discussion and written completion of the four elements of the recovery plan. This will allow further exploration of the critical ingredients of the study intervention and whether provisional findings from our development work (e.g. regarding the importance of the relationship between participant and PSW) are confirmed.

### Implications for policy and practice

This study generates two findings relevant to clinical practice. First, the positive feedback to additional peer-provided, self-management support following CRT discharge, both in principle from stakeholder interviews and in practice following preliminary testing, confirms the potential value of this type of support. The high perceived need from CRT service users and other stakeholders, for relapse prevention work to anticipate and plan responses to potential future crises, supports the recommendations for crisis planning in current expert guidance for CRT services [5, 37]. In the absence of a convincing evidence-base regarding the clinical effectiveness of peer support programmes in mental health services [27], our study provides some corroboration for existing national guidance [20] that peer support should be considered for people with severe mental illness as a means to improve service user experience and quality of life.

Second, our study highlights the need for careful planning to support the integration of new teams of peer support workers into established mental health services. Key lessons learned from our study confirm those from previous literature [33]. These include: the need to prepare crisis teams for having PSWs in their service; and fully preparing PSWs for the role of working within a health service, to enable integration of a recovery oriented intervention into predominantly medical-model services. Local team inductions for PSWs were arranged and these issues were included in PSWs’ training and supervision, in order to minimise administrative and cultural challenges to implementing the study intervention.

## Conclusion

The MRC framework [25] provides a useful guide to developing and evaluating a peer-provided, self-management intervention for people after a mental health crisis and a period of CRT care. Following a development and feasibility/piloting process, involving iterative stakeholder feedback, has helped to focus an intervention on the needs and priorities of the target client group, and to refine the intervention, its operating procedures and training programme, in order to support consistent implementation and replicability in a trial context.

## Additional files

**Additional file 1: DS1–DS3.** CRT Service User Interviews (stage 2)—participant characteristics. Stakeholder Focus groups (stage 3)—participant characteristics. Feasibility testing (stage 4) and piloting (stage 5)—participant characteristics

**Additional file 2: DS4.** CRT service user interviews (stage 2)—main themes.

**Additional file 3: DS5.** DS5 Stakeholder focus groups (stage 3)—main themes.

**Additional file 4: DS6.** Feasibility testing (stage 4)—main themes from participant interviews.

**Additional file 5: DS7.** Feasibility testing (stage 4)—main themes from PSW focus group.

**Additional file 6: DS8.** Pilot trial (stage 5)—main themes from participant interviews.

**Additional file 7: DS9.** Pilot trial (stage 5)—main themes from PSW focus group.

## Abbreviations

CORE: crisis resolution team optimisation and relapse prevention (research study)
CRT: crisis resolution team
MRC: medical research council
NHS: national health service
NICE: national institute for health and care excellence
PSW: peer support worker
RCT: randomised controlled trail
