# Supplementary material for: Development of a peer-supported, self-management intervention for people following mental health crisis
Source: BMC Res Notes. 2017 Nov 9;10:588. doi: 10.1186/s13104-017-2900-6 (PMC5680762; doi:10.1186/s13104-017-2900-6)
Supplement: Supplementary file 1 — Additional file 1: DS1–DS3. CRT Service User Interviews (stage 2)—participant characteristics. Stakeholder Focus groups (stage 3)—participant characteristics. Feasibility testing (stage 4) and piloting (stage 5)—participant characteristics [file 13104_2017_2900_MOESM1_ESM.docx]

**Table DS1. Initial CRT service user consultation (stage 2): participant characteristics**

| Gender | Male | 14 (33.3%) |
| --- | --- | --- |
|  | Female | 28 (66.6%) |
| Age group | 16-24 | 5 (11.9%) |
|  | 25-34 | 11 (26.2%) |
|  | 35-44 | 13 (30.9%) |
|  | 45-54 | 8 (19%) |
|  | 55-64 | 5 (11.9%) |
|  | 65+ | 0 (0%) |
| Ethnicity | White British | 30 (71.4%) |
|  | White Irish | 1 (2.4%) |
|  | White Other | 2 (4.8%) |
|  | Black Caribbean | 3 (7.1%) |
|  | Black African | 1 (2.4%) |
|  | Indian | 1 (2.4%) |
|  | Asian Other | 2 (4.8%) |
|  | Mixed White/Asian | 0 (0%) |
|  | Other mixed ethnicity | 0 (0%) |
|  | Other ethnic group | 2 (4.8%) |
| Number of times service user used a CRT | Once | 17 (40.5%) |
|  | Two to five times | 18 (42.9%) |
|  | Six to ten times | 7 (16.7%) |
|  | More than ten times | 0 (0%) |
| Most recent service user contact with CRT ended | Still receiving CRT support | 7 (16.7%) |
|  | Less than a month ago | 21 (50%) |
|  | 1 to 3 months ago | 10 (23.8%) |
|  | More than 3 months ago | 4 (9.5%) |
| Previous service user in-patient admission | Yes | 32 (76.2%) |
|  | No | 10 (23.8%) |
| Most recent hospital discharge | Less than 3 months ago | 15 (35.7%) |
|  | Between 3 months and a year | 9 (21.4%) |
|  | Between 1 and 5 years | 5 (11.9%) |
|  | More than 5 years ago | 3 (7.1%) |
|  | N/A | 10 (23.8%) |
| Service user has care co-ordinator | Yes | 22 (52.4%) |
|  | No | 14 (33.3%) |
| Diagnosis (reported by service user / carer) | Not sure | 6 (14.3%) |
|  | Affective Disorder | 21 (50%) |
|  | Psychotic Disorder | 11 (26.2%) |
|  | Personality Disorder | 3 (7.1%) |
|  | Unknown | 7 (16.7%) |

**Table DS2. Stakeholder focus Group Consultations (stage 3): participant characteristics**

| Category | Sub-categories | Service Users N (%) | Carers  N (%) | CRT Staff  N (%) |
| --- | --- | --- | --- | --- |
|  |  | n=20 | n=12 | n=41 |
| Gender | Female | 13 (65%) | 10 (83%) | 20 (49%) |
|  | Male | 7 (35%) | 2 (17%) | 21 (51%) |
| Age | 18-24 | 1 (5%) | 0 (0%) | 0 (0%) |
|  | 25-34 | 6 (30%) | 1 (8%) | 11 (27%) |
|  | 35-44 | 4 (20%) | 1 (8%) | 15 (37%) |
|  | 45-54 | 4 (20%) | 2 (17%) | 9 (22%) |
|  | 55-64 | 1 (5%) | 4 (33%) | 4 (10%) |
|  | 65+ | 4 (20%) | 4 (33%) | 0 (0%) |
|  | Not stated | 0 (0%) | 0 (0%) | 2 (5%) |
| Ethnicity | White British | 16 (80%) | 9 (75%) | 16 (39%) |
|  | White Other | 0 (0%) | 2 (16%) | 3 (7%) |
|  | Black Caribbean | 1 (5%) | 0 (0%) | 3 (7%) |
|  | Black African | 1 (5%) | 1 (8%) | 6 (15%) |
|  | Indian | 1 (5%) | 0 (0%) | 3 (7%) |
|  | Other ethnicity | 1 (5%) | 0 (0%) | 7 (17%) |
|  | Not stated | 0 (0%) | 0 (0%) | 3 (7%) |
| Self-reported diagnoses of self (service users) or family member (carers) | Psychosis (including bipolar) | 7 (35%) | 3 (25%) | - |
|  | Personality Disorder | 3 (15%) | 3 (25%) | - |
|  | Affective disorder | 4 (20%) | 2 (17%) | - |
|  | Multiple diagnoses | 3 (15%) | 2 (17%) | - |
|  | Not stated | 3 (15%) | 2 (17%) | - |
| Care Co-ordinator for self (service users) or family member (carers) | Yes | 12 (60%) | 7 (58%) | - |
|  | No | 7 (35%) | 5 (42%) | - |
|  | Don’t know | 1 (5%) | 0 (0%) | - |
| CRT use | Once | 4 (20%) | 3 (25%) | - |
|  | Twice | 5 (25%) | 1 (8%) | - |
|  | 3 to 5 times | 6 (30%) | 3 (25%) | - |
|  | 6 to 10 times | 1 (5%) | 3 (25%) | - |
|  | 10+ times | 4 (20%) | 2 (17%) | - |
| Professional background | Nursing | - | - | 29 (71%) |
|  | Social work | - | - | 4 (10%) |
|  | Medical | - | - | 3 (7%) |
|  | Clinical psychology | - | - | 1 (2%) |
|  | Occupational therapy | - | - | 1 (2%) |
|  | Pharmacy | - | - | 1 (2%) |
|  | STR worker | - | - | 1 (2%) |
|  | Not stated | - | - | 2 (5%) |

**Table DS3: Participant characteristics: feasibility testing and pilot trial participant interviews (stages 4 and 5)**

|  | Feasibility testing (n=9) | Pilot trial (n=18) |
| --- | --- | --- |
| Gender | Female (n=4)  Male (n=5) | Female (n=12)  Male (n=6) |
| Age | 25-34 (n=3)  35-44 (n=1)  45-54 (n=2)  55-64 (n=2)  Missing (n=1) | 18-25 (n=4)  25-34 (n=2)  35-44 (n=6)  45-54 (n=3)  55-64 (n=3) |
| Ethnicity | White British (n=3)  White other (n=1)  Black Caribbean (n=3)  Other ethnic group (n=1)  Missing (n=1) | White British (n=12)  White Other (n=3)  Black Caribbean (n=1)  Mixed race: White/Black Caribbean (n=2) |
| Number of CRT admissions | 1 (n=3)  2 (n=2)  3-5 (n=1)  10+ (n=2)  Missing (n=1) | not available |
| Diagnosis | Affective disorders (n=4)  Personality disorder (n=1)  Bipolar disorder (n=2)  Schizophrenia/psychosis (n=1)  Missing (n=1) | Affective disorders (n=8)  Personality disorder (n=6)  Bipolar disorder (n=1)  Schizophrenia/psychosis (n=3) |
