# Supplementary material for: Development of a peer-supported, self-management intervention for people following mental health crisis
Source: BMC Res Notes. 2017 Nov 9;10:588. doi: 10.1186/s13104-017-2900-6 (PMC5680762; doi:10.1186/s13104-017-2900-6)
Supplement: Supplementary file 2 — Additional file 2: DS4. CRT service user interviews (stage 2)—main themes. [file 13104_2017_2900_MOESM2_ESM.docx]

**DS4: Initial CRT service user consultation (Stage 2): main themes from participant interviews**

Results are presented in five primary themes of 1) acceptability; 2) what the intervention should contain; 3) When the intervention should take place; who should be involved in delivering the intervention; 4) where and how the intervention should take place. Figure 1 is a representation of these primary themes and sub themes.


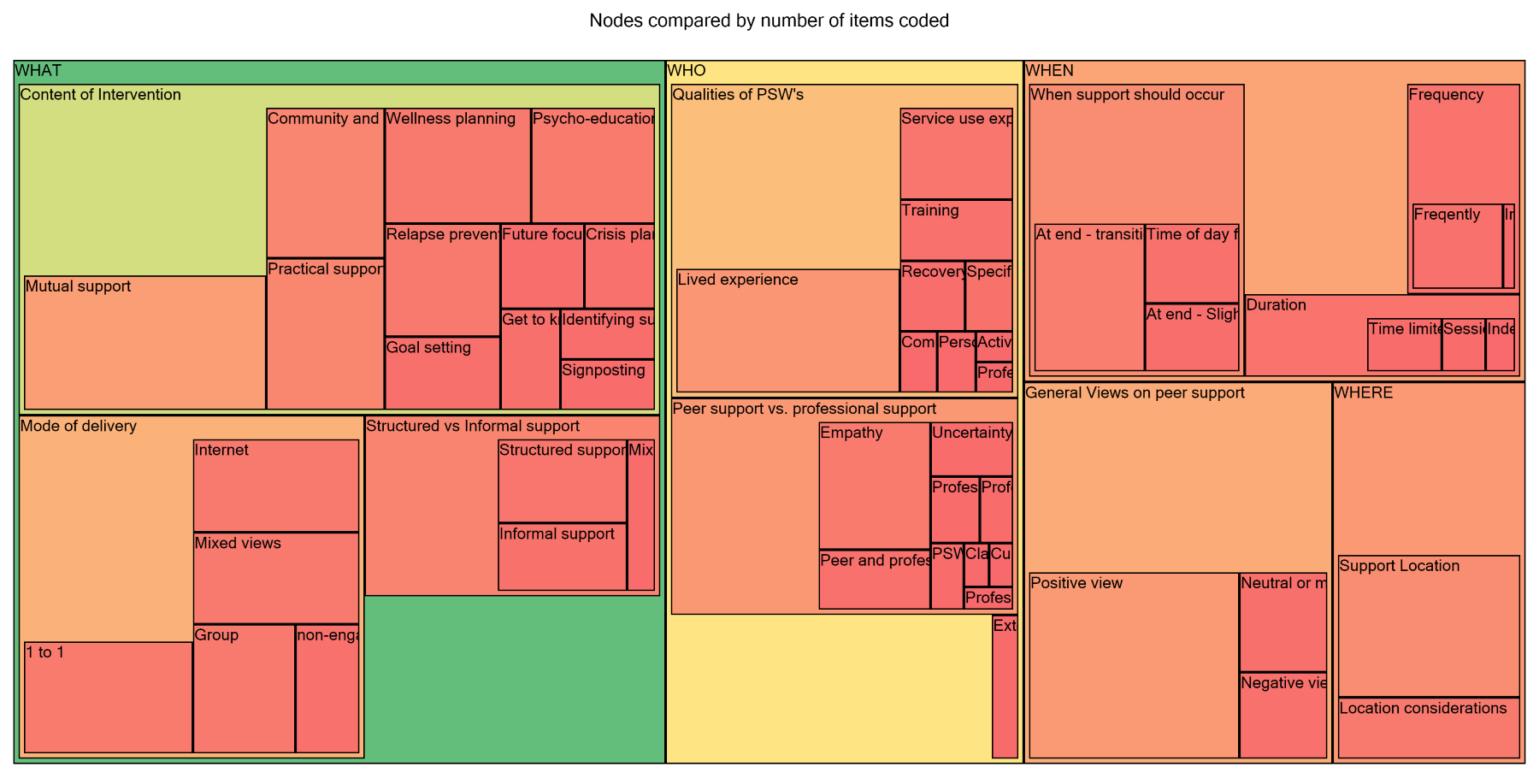


Figure 1. Primary themes, themes and subthemes by number of items discussed

**1. Acceptability**

As presented in Table 1, the majority of opinions of the peer support programme were positive, only six individuals held a negative view.

**Table 1. Participants’ acceptability of a peer facilitated programme**

|  | Sources | References |
| --- | --- | --- |
| **Total Views on peer support** | **39** | **57** |
| Negative view | 6 | 8 |
| Neutral or mixed view | 7 | 7 |
| Positive view | 31 | 43 |

Thirty-one participants (78%) expressed positive views about the idea of a peer support programme. Generally, participants felt that the peer support worker would offer something valuable:

*IE I’d be very much for it. It sounds like a very good idea. Whenever I’ve been an inpatient before, I’ve always found it very beneficial to be able to relate to other people that have been in similar situations; that they are experiencing the same things as yourself. And, likewise, I think it’s good for me to pass on my knowledge to other people, to have that general feeling of being able to help other people is very important to me. (*SU13)

*AL It's I suppose just as... I think it's great in a very different, unique kind of way. However experienced and qualified the staff are I don't think anything is as supportive as somebody who's been through the same services as a service user that you have. I think that's a really unique opportunity and potentially really helpful. For me anyway, there's something about the nature of when I get into these very sad phases, and it's to do with being in the house, isolated too much. So I think some kind of follow-on service where you have the responsibility to meet somebody somewhere else, not necessarily in NHS buildings or anything, it could be just having a coffee in the park, I think that that's a really good next stage out of... just making that small bit of progress and having social contact again that isn't necessarily just about meeting doctors and people and still being in the house and that kind of thing.* (SU28)

*IE I think that would be really helpful, obviously, knowing that someone who’s gone through possibly something similar or something else, then they kind of know… it could be like an inspiration, as well, if they’ve got themselves better, then you can think you can, as well. I think that would be really helpful. (*SU29)

Negative views towards the peer support programme were expressed by six participants (15%). These participants felt that they wanted to move on with their lives, away from a mental health setting.

*No, I don’t really think I need it; at the moment I don’t feel I need it. I feel that I want to... Now I feel well, I feel that I want to put it behind me and say, I am well and I’m going to continue to be well; I don’t want to go backwards and that’s the way I am. I think if I had more people still seeing me I think it’d make me feel as if I’m still not as well as I think I am* (SU01)

One participant raised the notion that they would need the person to be qualified as well

*If I… if it was somebody I knew and I knew that they’d got the qualifications in terms of assessment and everything else that they needed to have, then I think, yes. I think if it’s just somebody that’s had mental health problems with a little bit of extra training but has not had a lot of training or experience and is well suited, then I think maybe not because of the level of risk involved.* (SU25)

Mixed views about the programme were also presented by seven participants (18%). In general, people expressed it was a good idea, but at this current time they were unsure how it would be useful to them, that they already had sufficient support, or that they saw that there could be difficulties with implementation.

*I mean, they may well be helpful in certain aspects of it, but I don't know how they would be able to help me, other than, you know, certain specific things* (SU36)

*IE I don't know to be honest, because I have such a close family, I know that I’ve got… if I didn’t have any family, I didn’t have any support, then yes, that would be amazing,*(SU40)

*RD I think, sometimes it’s a good thing, but I think it’s also difficult.* (SU17)

**2. What**

The WHAT super theme was discussed by thirty-six participants. Their comments were divided into three themes of 1) intervention content; 2) the participants’ preferences for the mode of the intervention delivery; and 3) the participants’ preferences for the method of delivery. These three themes were broken down into sub-themes to add a depth of understanding to what individuals wanted the peer facilitation programme to include. All items are presented in Table 2.

**Table 2. What individuals want from a peer facilitated programme**

|  | Sources | References |
| --- | --- | --- |
| **WHAT** | **36** | **220** |
| **Content of Intervention** | **34** | **141** |
| Mutual support | 27 | 46 |
| Practical support | 15 | 27 |
| Community and social engagement | 15 | 26 |
| Wellness planning | 14 | 16 |
| Psycho-education | 12 | 17 |
| Relapse prevention | 11 | 16 |
| Goal setting | 7 | 7 |
| Future focused | 6 | 6 |
| Crisis planning | 5 | 8 |
| Get to know person | 5 | 7 |
| Identifying supporters | 4 | 6 |
| Signposting | 4 | 4 |
| **Mode of delivery** | **29** | **63** |
| 1 to 1 | 16 | 17 |
| Group | 11 | 16 |
| Internet | 13 | 16 |
| Mixed views | 13 | 16 |
| non-engagement with certain modes of delivery | 7 | 8 |
| **Method of delivery** | **18** | **25** |
| Structured support | 10 | 12 |
| Informal support | 8 | 9 |
| Mix | 4 | 5 |

**2.1. Content of Intervention**

As presented in Table 2 some major theme that generated by participants that related to the content of the intervention included mutual support, practical support, and community engagement. Twenty seven participants (68%) generated the idea that mutual support was particularly important. Reasons for this included having someone to share their experiences, challenges and knowledge with in a bidirectional fashion.

*IE I’d like to talk for an hour with them, see what experiences we could exchange. Exchange experiences, see if that person knows about it, what experiences they are. If the person is like me who’s had mental health problems, I think they’d understand me more and then they’d understand themselves and understand my problems and they’d understand the sort of problems I’ve been having, if they had mental health problems as well. They’d be experienced and that’s a good thing, really, like that.* (SU14)

Practical support was seen as potentially useful part of the peer support for fifteen participants (38%). This might be to help to plan and problem solve around day to day issues such as shopping, arranging appointments, or searching for employment.

*Sometimes practical things like shopping. Not necessarily to do it for you, but helping you to find ways to deal with it.* (SU12)

*We’re going to maybe do a plan of how to move forward from here, and basically how to move on, maybe eventually, not now, but eventually, how I can get a job and to build up my confidence,* (SU15)

*I think being there at the meeting, the appointments, being able to come in with me ... somebody that could relay back to me the appointment and things that I might have missed.* (SU08)

Community engagement was important for fifteen participants (38%) as it was an opportunity to get out of the house or mental health setting.

*I think that perhaps the best thing is to go out for coffee. It’s good for people who have been in hospital to learn to go out again, to get out. It’s important, because sometimes, you can become stuck in your home, feeling like a prisoner.* (SU05)

*I think would work for me because it would get me out, get to meet people and talk to them and get help at the same time, so I think that is a time. I think that a lot of people would like that.* (SU32)

Mental health related features including wellness planning (35% of participants), psycho-education (30% of participants), relapse prevention (24% of participants), and crisis planning (13% of participants) were all also seen as important features of the peer support.

*IE Experience with the mental health, ways that they’ve learnt to cope with it, and how they have avoided problems - I guess, stuff like that. Like avoidance, dealing with it, how to communicate when you need help, stuff like that.* (SU32)

*IE I’d just like, like they said to me, that they’d draw up a care plan and a step by step… especially about relapsing. I’d like support on recovering and not relapsing again. That’s what I got last time.* (SU07)

*IE I think I would have found it helpful last time, which probably might have prevented me from getting as bad this time if I was… because I think if you… I do know a lot of people with mental health problems, but I do think that someone who’s been through it themselves can help someone better than someone who hasn’t, so I think to bring someone in like that while you’re still in the crisis would be a good thing because they could actually being through it themselves to help ways to stop feeling like it maybe.* (SU19)

**2.2. Mode of delivery**

Although all types of delivery including one on one support, group based support and internet support were relatively equally distributed, there preference was ultimately for one on one. There are a number of reasons for this; firstly, one on one was seen as the most acceptable by 40% of participants.

*IV Would it make a difference if you saw that person within a group, one to one, or a mixture of both; what would work best?*

*IE Initially, for me, I’d opt for one to one* (SU08)

Secondly, although groups were acceptable for 28% of participants, they were also not acceptable for some.

*IE Yes, I think one-to-one, because I’m not always comfortable in group situations, so I’d have to have that understanding on that aspect. (SU13)*

*But there is something to say for one to ones because there are some things that are specific to you that you might not want to share with other people.* (SU31)

Thirdly, although internet was seen as useful, some may not be able to engage in this type of modality for various reasons such as access or skills, or saw it more as a booster to face to face support.

*IV What do you think about things like chat lines, and web based relapse prevention plans?*

*IE That’s all right if your computer is working. My computer has been broken since November, and I’m very upset about it, and I’ve got to get a new one, I’ve got to get it replaced.* (SU05)

*IV And do you think any online resources or any other sort of resources might help you in order to really just look at how you can avoid future crisis and continue recovery?*

*AL I'm of the generation that does use online life quite a lot and it would definitely be something that I would use if it existed. But that's not to say that it should all be online and exclude people who don't interact with that.* (SU28)

*IV You’re obviously a young person. I’m interested in the type of structure which it should have. Would it need to be face to face with you or could the internet, as an example, or text messaging or something like that work for you?*

*IE I think it would need to be a mixture. I think you’d feel more comfortable if you saw her face to face first and were able to build up the relationship. I think using the other mediums would back it up.* (SU29)

**2.3. Method of delivery**

Structured and informal support was relatively equally acceptable to participants. The benefits of a structured support was that it gave direction to the support.

*IE I think it should be structured. I think structured would make more sense, or you could get carried away and start giving somebody else a whole heap of your problems, which isn’t appropriate.* (SU05)

The benefit to informal support that it removed formality and stress.

*IE Yes, I think more like a friendly chat, to be honest. Yes, I think anything that feels as natural as possible, to be honest, I think… otherwise it’ll just feel like homework* (SU40)

Some felt that being able to offer both was key, as it allowed the flexibility to adapt to the needs of the individual.

*That would depend on the person's mental health needs.* (SU12)

*I don't suppose anyone's going to feel exactly the same every week. Sometimes you might really like to say, I don't want to talk about how I feel, can we just look at the plan? Or, I just want to talk about how I feel, I don't want to look at the plan, that kind of thing. To be flexible I think is probably the most important, but a bit of both.* (SU28)

**3. When**

The WHEN super theme was discussed by twenty seven participants. Their comments were divided into three themes of 1) duration of the intervention; 2) the frequency of the programme; and 3) and the timing of the delivery of the programme/when the support should commence. These three themes were broken down into sub-themes to add a depth of understanding to what individuals wanted the peer facilitation programme to include. All items are presented in Table 3.

**Table 3. When themes**

|  | **Sources** | **References** |
| --- | --- | --- |
| **WHEN** | **27** | **51** |
| **Duration** | **8** | **11** |
| Indefinite support | 2 | 2 |
| Session length | 3 | 4 |
| Time limited | 5 | 5 |
| **Frequency** | **10** | **11** |
| Frequently | 8 | 8 |
| Infrequently | 1 | 1 |
| **When support should commence** | 8 | 8 |
| At end - transition from CRT to PSP | 15 | 19 |
| At end - Slight break between CRT and PSP | 6 | 6 |
| Time of day flexibility | 7 | 8 |

**3.1. Duration of sessions and programme**

Whenever participants (8%) commented on session length it was always presented as 1-2 hours being the ideal. For the eight participants (20%) who commented on the duration of support, the majority felt comfortable with time limited support with only 2 participants’ indicating that it should carry on until it is not needed.

*That would be nice obviously but I understand that there are other people that sometimes, when you get better don’t you, you get better and then you don’t need them, although you start depending on them don’t you, forever? That’s not a good thing. So I think it’s best to move on really and let someone else get their care from them who need it more once you’re better.* (SU26)

**3.2 Frequency of sessions**

Participants who commented on the frequency of the sessions generally suggested that regular sessions were important for routine and to see progress. Weekly sessions were the most frequently suggested norm.

*IE Well, it could be, have a plan for maybe a weekly thing, for you to meet with the other people that have been through it, and then just to…I think what I am going through is there is lack of motivation in everyday life; I don’t know; I think I was combined with depression as well as psychosis, so when you’re in depression, even walking out of the house is a struggle. And so, yes, a weekly basis, where you could see yourself getting better every week – that would have been good.* (SU15)

**3.3 When the support should commence and sessions should occur**

All participants (n=21; 53%) who commented on when the Peer Support Programme should commence suggested that near the end of the CRT support would be most beneficial. The majority of these (33%) liked the idea of a smooth transition between the CRT and the PSP which would be incorporated to their plans and assist with continuity of care.

*I think it would probably be a good idea to bring it in towards the end, so there’s a slight overlap, so you’re still getting continual stream of so you know what’s going on, whereas if you stop one, there might be a gap till that one starts, and it’s going to be less effective. As far as I can see anyway, so yes, just integrate it into the last little bit with your crisis team, because then they know you, and they can tell them things about you that you might not be able to tell them, so they know where they’re going from.* (SU11)

*I think it should at least start before you’re being discharged so that you’re not lost in the system, basically.* (SU07)

A few suggested that a small gap would be useful, particularly so the peer support work was seen as separate to the CRT and the crisis itself , so that people had time to get back to their routine, or so as to reduce over-servicing and confusion.

*I’d say probably a few weeks just to let things breathe a little. I think if you saw them straight away, it might get a bit too intense, and I think it would be easier to differentiate if you had that gap between, because I think if you started seeing them straight away, they’d be too closely linked to the crisis team, and you’d think you’d have a similar experience, maybe.* (SU29)

*I think a few weeks could be good, because that way it gives you time to kind of get back to normal, get back into your routine, and it also gives you a point where you’re going to have another contact with services and some more help to sort out stuff. (SU34)*

*Not straightaway, because like I say too much information at one time is just... with different people coming I got confused.* (SU22)

Additionally, participants who commented on when they would like to have a session, they suggested that it was important to have flexibility as they had varying needs.

*IE Yes, I think, again, something you can click into when you want; being too... I’m trying to look for the words now, being restricted to a regular thing that you have to do, would probably be a bit too difficult for me. I’d like something that I could just jump in and out of and use the service if I need to, rather than be restricted to times or a plan as such like that.* (SU08)

**4. Where**

**Table 4. Where themes**

|  | **Sources** | **References** |
| --- | --- | --- |
| **WHERE** | **22** | **42** |
| **Location of support** | **21** | **32** |
| Home | 15 | 17 |
| Avoid certain locations | 6 | 6 |
| Personal choice | 6 | 7 |
| Neutral place | 4 | 4 |
| Comfortable location | 3 | 3 |
| **Location considerations** | **9** | **15** |
| Use as social outlet | 7 | 9 |
| Use location as goal | 3 | 4 |
| Consider Privacy | 2 | 2 |
| Consider transport costs | 1 | 1 |

Twenty two participants (55%) commented on where they would like to receive peer support. The major theme across ass sub-nodes was that although home was a preferred location, it should be flexible, comfortable, up to the individual to decide, to then and that certain locations they felt were inappropriate.

*IE Again really down to where the patient feels comfortable; whether it’s in their home; whether it’s in the hospital; whether it’s in just a coffee shop, somewhere, anywhere really the patient feels comfortable.* (SU13)

Furthermore, some considerations for location were also put forward suggesting that it could be used as a social outlet for isolated individuals, it could be used as a goal to more forward, and that practical issues such as privacy and transport should be considered.

**5 Who**

**Table 5. Who themes**

|  | Sources | References |
| --- | --- | --- |
| **WHO** | **35** | **105** |
| **Peer support and professional support** | **24** | **41** |
| Peers bring empathy to support | 13 | 17 |
| Peer and professional roles can complement each other | 6 | 8 |
| Unsure about preferences | 4 | 4 |
| Professional role viewed as more valuable | 3 | 4 |
| Professionals having mental health issues | 2 | 5 |
| PSW role valued by team | 2 | 4 |
| Clarity about peer role | 1 | 2 |
| **Qualities of PSW's** | **31** | **73** |
| Lived experience | 24 | 39 |
| Service use experience | 9 | 12 |
| Training and competencies | 6 | 8 |
| Recovery stage of PSW | 4 | 6 |
| Specific illness experience | 3 | 9 |
| Personal qualities | 2 | 2 |
| Community knowledge | 2 | 2 |

**5.1 Peer Support compared to professional support**

A proportion of participants (33%) suggested that a peer support worker can bring an added level of understanding and empathy to a role, which may not be fully provided by a health professional.

*But, yes I think that someone who’s had mental health before you’ve got the experience, if you’ve recovered you’ve got the experience and you can understand, you can empathise, you can understand exactly what the person’s going through. As a professional you’ve got all the knowledge and everything, but you haven’t been in the person’s shoes. It’s hard, it’s like you’re reading a book, but when you have mental health, you’re in the book and you can’t get out of the book.* (SU27)

*It's I suppose just as... I think it's great in a very different, unique kind of way. However experienced and qualified the staff are I don't think anything is as supportive as somebody who's been through the same services as a service user that you have. I think that's a really unique opportunity and potentially really helpful.* (SU28)

Two individuals noted that they had found it particularly useful when a health professional had experienced mental health issues themselves. Overall, the role of the peer and the health professional could be viewed as complimentary, with both being important and useful and should ultimately work together.

*IV And if you had the choice between a bit more support from the same crisis workers as you’d already been having, and support from someone who’d used mental health services themselves, which would you go for, do you think?*

*IE Depending on the situation really, I guess if I felt strongly that I just wanted to talk to somebody about… and if I knew I could contact someone who had previously using the service, then that’s the step I would take initially. But then judging whether that could actually develop into a crisis situation, then it’ll be a case of contacting the crisis team. But then having said that, having seen someone that’s been a service user previously, it would help for them to know that they haven’t got the professional experience, but it would be helpful for them to maybe decide in taking that step to contacting the crisis team.* (SU13)

A small number of participants (10%) were unsure about who they would want supporting them, and some (8%) held a preference for receiving support from mental health professionals. For the PSW to work effectively, two participants raised that they would need to be valued by the CRT team and one of these participants also suggested that clarity about the boundaries of the PSW role was important.

*And that’s a very difficult question because… but it’s also… it’s not necessarily to do also with the person but it’s about, because I’ve got a couple of friends that are peer support workers, but it’s also about the place in which a peer support worker sits because, in some ways, they’re neither completely somehow a member of staff, or a service user. They’re kind of stuck in the middle, and there’s sometimes a conflict there and I think it’s quite… it’s quite difficult boundary-wise. It can a bit confusing* (SU25)

*Somehow, as a peer support worker, you’re not on equal path with, inverted commas, the professionals in the team. It’s still early stages in being equal… people’s views, seeing people as equal but I think the, I don’t know whether the labels particularly help; some people it might, some people it might not. But, yes, I can’t sort of… all I know it works for me and I don’t think there’s a definitive answer.* (SU25)

**5.2. Qualities and Skills of a Peer Support Worker**

Lived experience was highly linked to empathy. Many expressed that they would like a peer to have a lived experience because it increased understanding and could assist with formulating effective coping by learning from others.

*And that’s the gold about it, is that people who have mental health issues are the only ones who can understand. So, that’s why they’re special, because nobody else can really understand and they’re the only ones that can really take away the utter isolation that you feel.* (SU23)

*But if I was talking to someone else, I don’t think I’d need to say half the things I’ve said because we’ve got shared experiences and can share coping strategies and can actually talk about what helped them and see how I could adapt it to help me.* (SU31)

Previous service use was also seen as useful in helping understand and negate the system.

*somebody who knows a bit more, understands the jargon, or understands what they’re saying and things like that.* (SU08)

*I think another service user would make a difference because they're more understanding because they've been through the system, so they'd know how the system works.* (SU12)

Knowledge, training and competency was seen as very important to service users (15%), so that they felt they were in safe hands. A variety of different training modules were viewed as important outside of just working with mental health conditions, such as promoting empowerment, equality, diversity.

*So it’s not actually to do with the person’s mental health identity as such. It’s more about their competence of dealing or knowing how to deal with me and having the level of authority to get me the help and the level of skill as well* (SU25)

*And I think they should actually promote empowerment, assertiveness, give them training around things like leading focus groups, around things like being mental health champions, around things like being able to sit on the different groups, say for example equality and diversity. There’s no point in the Trust saying we want people to sit on the equality and diversity group. Have you educated or given information on what the act says or indeed not only just on the act, have they been involved where they can sit and talk to other people, be it staff or other service users? What does diversity mean to them? What does equality mean?* (SU31)

Some suggested that the recovery stage of the PSW was important, as someone further down their recovery journey may be able to more effectively support people.

*if you’ve recovered you’ve got the experience and you can understand, you can empathise, you can understand exactly what the person’s going through.* (SU27)

Furthermore a specific experience of an illness would be useful (8%).

*I would love to have somebody who experienced psychosis and who would come forward and he would tell me that I have experienced this, this and this, and it’s completely normal and I’m fine.* (SU21)

Finally a peer support worker having local knowledge and having personal qualities such as being nice were seen as important to a small number (5%) of participants.

**Summary of findings**

The data from this sample suggests that a peer facilitated self-management programme is acceptable to service users and most would be willing to take part in such a programme after leaving crisis services. What this would entail would be a programme that offers a combination of mutual, practical, social and mental health support. A one on one programme delivery was seen as more preferable, with flexibility incorporated into all facets of the delivery to suits the needs and goals of the participant. This type of programme was seen as complimentary to professional services as the lived experience of the Peer Support Worker may offer a unique and empathetic perspective to an individuals’ support experience.
