# Supplementary material for: Development of a peer-supported, self-management intervention for people following mental health crisis
Source: BMC Res Notes. 2017 Nov 9;10:588. doi: 10.1186/s13104-017-2900-6 (PMC5680762; doi:10.1186/s13104-017-2900-6)
Supplement: Supplementary file 3 — Additional file 3: DS5. DS5 Stakeholder focus groups (stage 3)—main themes. [file 13104_2017_2900_MOESM3_ESM.docx]

**DS5: CRT stakeholder focus group (Stage 3)_Main Themes**

**Results:**

Results are presented in seven primary themes of: 1) Overall impressions- View of PSW intervention; 2) Pre-commencement considerations; 3) Commencement; 4) Content and delivery; 5) PSW qualities and skills; 6) PSW supervision and support; 7) Ending the intervention. Overarching themes and subthemes are presented in Figure 1.


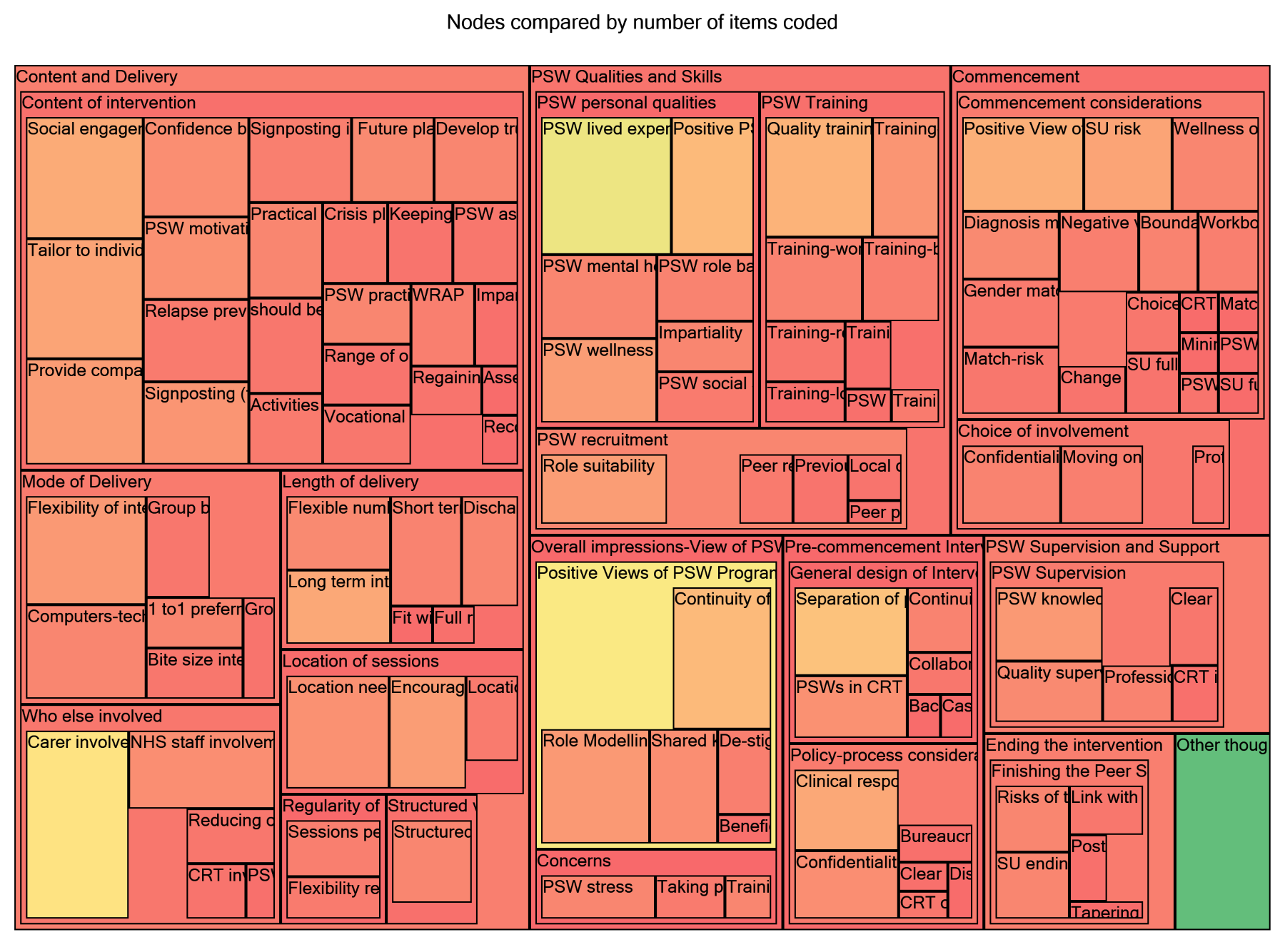


**Figure 1. Stage 3 overarching themes and sub themes**

**1 Overall impressions**

**1.1 Positive views of PSW programme**

When participants were asked for their initial impressions of the proposed Peer support self-management programme, the majority were very positive, describing the project as a ‘brilliant idea’. The aspects of the proposed intervention that seemed to promote positive views were the peer led nature of the support and also the proposed timing of the intervention.

**1.1.1 Shared knowledge and understanding (11 sources, 33 references)**

A number of people (1 carer, 5 Service users and 5 members of staff) expressed how beneficial they thought it would be to have someone who has had experience of mental ill-health to support others in a similar position. The reason for this centred on the shared knowledge, empathy and understanding gained by experience.

*PA when people with mental health issues do get well, they've got the empathy, and they've got the understanding, and they learn a lot about mental health, they learn a lot…I know people with mental health problems that are well, and they do a lot of stuff, they're involved in a lot of things, and they know nearly as much as the professionals, because that has been their role. (Carer – site 1)*

*PA For instance, someone who hears voices, and in their head and things like that. And, maybe that person who’s looking after, not looking after you, but supporting you, can say, yes, I know what you’re talking about, you know? It’s just been helpful for them, maybe someone to identify what you’re saying, and have some more greater understanding than maybe a lay person will.* *(service user – site 2)*

*PA Plus with, like, mental health professionals, most of them are just reading it out of books and stuff they’ve learnt. They’ve not actually experienced it so they don’t know it for themselves, so it would be good to talk to someone that’s been through it, as well, and see how they’ve found ways of getting help and stuff like that.*(service user – site 3)

One service user and one carer also expressed the view that this shared knowledge and understanding could be ‘beneficial to both parties’ and the opportunity given to the peer to help someone else may help, ‘keep themselves well’.

**1.1.2 Role-Modelling (8 sources, 30 references)**

A number of people spoke of a peer potentially being viewed as a role model by service users, someone who has successfully recovered from their mental health problems and is now well.

*PA A lot of people really are inspired and feel empowered by someone who has recovered, or has gone through the same processes as them, and it's like a model for them to look up to and say, well if you've done it, I can do it too. So there is that very positive aspect. (Carer - site 1)*

One service user spoke of the impact that having a peer as a role model can have on their self-efficacy.

*PA They make you feel, you know, as if, that you’re not stupid…They teach me and I feel really enlightened and feel a bit better, to say I can go out there, whether I’ve been through mental health or whatever I’ve been through. I can, I’m somebody, you know? Just like them, I’m somebody. (service user – site 2)*

A small suggested that caution must be taken with seeing peers as role models because of issues such as stigma, and that experience did not always equip a person with the necessary skills to be a good role model.

**1.1.3 De-stigmatising (3 sources, 6 references)**

Three people (one carer and two service users) expressed the view that a recovered peer providing support could have a destigmatising effect for service users.

*PA I think it's a really good idea, because somebody with mental health issues will understand what someone else has gone through when they're in crisis, because there's a lot of stigma around mental health, and that's the reality unfortunately. Some people don't want to know, fear of it may be contagious. So somebody who's gone through it, it's also something that my son would relate better to somebody who's been through a similar experience and how they're managing.(Carer – site 1)*

**1.1.4 Continuity of care and bridging gap in services (9 sources, 22 references)**

A number of people (five service users, two carers, and two members of staff) highlighted issues related to continuity of care and bridging a gap in the mental health services received by the service user. Both carers commented on themes of a need for continuity which in their experience wasn’t fulfilled by the CRT alone:

*PA The crisis team in our case was really admit, not admit, monitor. That's really all they did. There was not continuity, there was a lot of hanging around waiting for people to turn up* (Carer – site 1)

Both staff members agreed that there is a gap in continuity post-CRT involvement which is not filled by existing services, and that this gap is a disadvantage for the service user’s recovery:

*PA […] when we discharge them they are in limbo, they feel, like, deserted, rejected, because we’re leaving them. And then we try and decide, as I think Sam said, the various services, we don’t know which one will suit them well depending on their degree of their illness, their recovery [?] [unclear], like, they see CRT, the intake team, the EIP team and then they are left on their own, and then, you know, they don’t have that regular support*.(Staff – site 4)

*PA I think we do see some people that could benefit from that little bit more after we’ve sort of, after our treatment episodes have finished, but they don’t fit the CMHT criteria or anything else. And then at some point they usually do end up coming back to us because there isn’t anything else for them and then maybe something like this could be good for them.* (Staff – site 1)

A number of service users expressed concerns related to a lack of continuity of care under current services:

*PA See, in my personal situation, I’m not involved with anything except for my drug and alcohol, so anything to do with mental health I’m not involved with [unclear]. They sent this email, access these particular people [unclear], but they’ve not got back to me at all, so I’m left high and dry and that’s a major concern for me. But I’ll just have to get on best I can.* (Service user – site 2)

*PA Like I’ve been seeing my psychologist for a good… for six years now, and it’s coming to an end now, and Home Treatment know that and they know how important that was to sort of look after me, but they haven’t put anything in place for afterwards, so I’m sort of losing two things at once.* (Service user – site 4)

*PA Now, if you are coming out of a Crisis or Home Treatment Team and then you somehow don’t make it to that day service which would be something that could help you, you know, help stabilise you and get routine in your life and lead to less isolation for you, if you can’t get into that in the community, then it just makes things a lot difficult because a lot of the support and places where people could go have actually been closed down.* (Service user – site 4)

One service user suggested that continuity of care could be implemented through support from the PSW in continuing with the service user’s established care programme:

*PA I think for me the most important thing is the feeling that from the beginning there’s some kind of continuation. When I was using the services here as an inpatient I was given a care programme and maybe it was just my experience, but that was written and agreed on and then not carried on by the Crisis team and I think, if they’re going to start with something at the beginning, to continue with it is really important. So to use that as a focus for... because it comes with really nice aims and this is what we’re going to do to achieve the aim. I think if people could use that and carry on and see some sort of, well, we’ve worked on this and actually we can say this is pretty much done now and then move onto the next and so some kind of working towards completing something, rather than having that and then never looking into it again.* (Service user –site 5)

**1.2 Concerns about the PSW programme**

Concerns about the PSW programme related to themes of whether the programme would replace existing professional services and whether training and support systems would be adequate to equip the PSW with the necessary skills to carry out the role.

**1.2.1 Taking place of professional services (3 sources, 5 references)**

Two carers and one member of staff commented on the possibility of PSWs taking the place of professionals. The two carers raised concerns that PSWs might be used to replace existing services. A specific concern was raised that replacement of professional services could be a result of budget cuts. There was a suggestion that the PSW programme is used as an add-on to, rather than replacement for, professional services:

*PA […] I think the professionals are the ones that should be doing that job, not the peers. They should all be in a group, because they're not qualified. They know how they feel, but…* [*(Carer*](file:///C:\Users\rejukfu\AppData\Local\Microsoft\Windows\Temporary%20Internet%20Files\Content.Outlook\55ZWPWJ7\1f6fea73-fba7-449e-a8d0-237f5e7a2f2d) *– site 1)*

*PA This is another thing that's just come to my mind because of all the cuts in mental health, is this a way of people… like a deselection instead of staff, using people as volunteers? That's just the cynic in me, but that's the reality. Because I used to work for a charity, and if the volunteers weren't there, because them volunteers, they were fed up of being taken for granted, volunteering has got a lifespan.* (Carer – site 1)

*PA to me, it’s a brilliant idea provided it doesn’t take the place of what’s already in place, i.e. you become a bit suspicious of the cuts and things, obviously. We don’t want it to be taking the place of anything. That would be nice to be in addition to, and it sounds like a brilliant idea to me.* (Carer – site 3)

One member of staff suggested that funding should be directed towards existing services rather than the PSW programme:

*PA I think it’s a shame that the extra money can’t be pumped into the crisis teams and the CMHTs, and that the CMHTs can’t… sometimes we end up with people for a lot longer than we should because other services don’t have the resources and it just feels really sad that you’re putting all this money into this when other services are so needing at the moment.* (Staff – site 1)

**1.2.2 Training and support systems (2 sources, 8 references)**

Two members of staff highlighted concerns relating to training and support systems for PSWs. The importance of training for PSWs was raised, rather than viewing PSWs as qualified on account of their having experienced a mental illness:

*PA […] I suppose, I don't think being ill in itself means that you're good at doing anything in particular, so it’s important not fall into that trap, right, just because you've been unwell you'd be good at supporting people. […]* (Staff – site 2)

One staff member commented that working within their own care system might present difficulties if the PSW becomes ill:

*PA If I became unwell, Occupational Health would make a recommendation for me, and the Trust itself would make sure I did some form of phased return and be gently reintroduced into work. For a service user, there is none of those monitoring things that go on currently within the Trust, so they can become very ill, become well, take on too much responsibility within their role that they do, and then they’re ill again. But within that, our relationship with them breaks down because they see us as having called the Mental Health Act on them, been involved in all sorts of things with them, so we have… it’s very difficult…*  (Staff – site 3)

The nature of the training was questioned and need for evaluation highlighted, with a suggestion that one week of training will not be enough to equip the PSWs:

*PA But you wouldn't take that and say she can just, or he, could meet up with someone and that someone is a trained health person, meet the people for a week and then go out and support people. So in a sense it’s slightly perverse in that sense in that the training for professionals is very highly specified, whereas things like training for a vulnerable group is the exact opposite, so you do it as quickly as you can. And I suppose it’s easier when you train people, you then evaluate whether the training’s been successful because usually there's an examination or an observation or something to see if it’s actually penetrated. But I'm not sure that's built into this training.* (Staff – site 2)

*PA So they're have to assess what they present, so they're not told anything other than... and assessing the needs of that client based on a week’s quite loose training really. So it’s not good.* Staff – site 2)

**1.2.3 PSW stress (5 sources, 8 references)**

Four members of staff and one carer commented on the possibility of the PSW experiencing stress through the programme. The carer highlighted the need for support for the PSW and questioned how well the PSW could cope with situations of stress:

*PA Yes, I'd like to say in regards to having support peer, they themselves have had a mental problem. And obviously to deal with somebody else's, not actually deal with it, but to support somebody else who's got a mental issue, it would be quite difficult. I mean like Karen, sorry, I won't mention names, like the lady over there said, that it makes them feel that they're doing something good, that's one way of looking at it. But the other way is that it's putting them under a great deal of pressure to do something that is quite difficult to deal with another mental health patient.*

*I think it's a good idea in them supporting each other, and obviously applying the right sort of person to someone who's got the same sort of illness, but sometimes it can work the other way, that person can feel not so good about relating to someone else.* (Carer – site 1)

The staff members gave examples of situations that could cause the PSW to feel under pressure:

*PA I think that if they’re going to do a peer group I think there should be a member of staff present, because my concern is that some people will say, I’m really suicidal, and I think that puts a lot of pressure on people with mental health problems who are going to have to mop up the pieces and that they might not actually have the skills or ability to deal with that.* (Staff – site 1)

*PA As I said, I think the selection of the peer group that are going to be trained needs to be quite robust. I think it's a very, very good idea but it's, I think that there can be, there could be times when it's, it could be detrimental.* (Staff – site 5)

**2 Pre-commencement considerations**

**2.1 Policy-process considerations**

Participants discussed considerations of risk policies and safety processes, as well as the general bureaucracy of the PSW programme, that would need to be resolved before the start of the programme.

**2.1.1 Discharge delay reduction (1 source, 1 reference)**

One member off staff suggested that PSW programme could help those who have been discharged:

*PA {…] we’re trying to get people off our books as quickly as possible, and this is one way of supporting those people who have been shift off our list a lot quicker than they would have been.* (Staff – site 4)

**2.1.2 CRT capacity (1 source, 4 references)**

One member of staff made several comments related to ‘pressure’ on CRT services in the PSW programme. These regarded expectations of what the CRT would provide as well as time pressures from additional meetings with PSWs and possible interference from the CRT:

*PA Well, I think there's a couple of things. We get involved in so many things, I think that's one of the things, that it would just be some days with the team we might have ten meetings with different people on our board, and then to have three more of peer support workers, I don't know if that's actually logistically feasible. And then the other thing is that I suppose maybe this is meant to be something that if the idea is it’s between someone who’s got mental health problems and the service, so maybe that should just be between the two of them rather than us bulldozing in and saying I think these are her problems and what you want to talk about.* (Staff – site 2)

**2.1.3 Bureaucracy (2 sources, 2 references)**

Two service users and one member of staff made comments relating to the bureaucracy of the PSW programme. The service users questioned whether PSW contracts would be permanent or temporary; and asked about the NHS recruitment system. One of the service users questioned whether PSWs from a previous stage of the project could continue to work in the current programme:

*PA Then you want different people for the… can’t you just keep the same people?*

*IV Unfortunately, because it’s NHS jobs it’s open competition so it gets… I mean the same people can apply, but also other people can apply as well.*

*PA That doesn’t make sense to me.(Service user – site 4)*

The staff member questioned who would have responsibility for problems that arise during the PSW programme:

*PA And the other issue is really if there was any problems and someone had made a decision, who’s responsible? Is it the team or is the peer support worker, psychologist? And on what basis, and people say, well, I will be responsible for that case, if it’s illegal, yes, that would be interesting.* (Staff – site 2)

**2.1.4 Confidentiality (5 sources, 10 references)**

A number of people expressed views on issues of confidentiality that could arise during the PSW programme. Two service users acknowledged the limitations of confidentiality but stated that they have no personal worries about it, although one suggested that other service users could be worried:

*PA I think it depends. I wouldn’t be worried, personally, but I know some people are a bit more cagey about their problems; they’re ever so uptight. I wouldn’t be worried myself personally, but I suppose it would depend how severe the nature of people’s problems are, whether they’re worried or not.* (Service user – site 3)

*IV But if it was somebody else with experience, would you worry about them breaching your confidentiality, breaching your confidence?*

*PA No, I don’t think I would because if I told someone something and obviously they were worried and told someone else, then I know that… no. Well, at the time I might be angry but in the long run I’d know they were doing the right thing because they’ve probably been in the same situation as as I’m in then. So I think it would be good for them to tell someone that, you know… I mean, obviously if I told them that I’m feeling really suicidal or something but don’t tell anyone, and then I went and done something then they’re going to feel really bad, so something like that I feel they should then tell someone, yes.* [*(Service*](file:///\\ad.ucl.ac.uk\slms\group\MHS\Johnson\CORE%20programme\Peer%20support%20Qualitative%20Paper\NVIVO\Stage%203%20-%20focus%20groups%20-%20carers%20-%20staff%20-SUs\584c2657-e933-4a27-80d0-237f61eaa230) *user – site 3)*

One carer established with the interviewer that the PSW would be ‘bound by the same confidentiality as any other health professional’ (Carer – site 3) Three members of staff raised concerns about confidentiality. Issues included what should be reported by PSWs to health services and how PSWs will know what to report:

*PA And it’s not even just about risk though in that sense. It’s about the fact that often our patients struggle to express themselves or they might not get the story quite right. And actually what the value of someone going through a sort of risk in the crisis plan or relapse or certain things without any knowledge of their past history, I don't know. But, as you say, it’s difficult because they're not professionals that you can give them the information. I don't know. […] Unless there was a referral form or whatever that you did it together with the client or something, so that at least you could put down there, you actually agree that, their word. In that way you could say to them, well maybe do you think we should mention this.* (Staff – site 2)

Another issue raised by a staff member was whether PSWs would be able to access confidential service user notes:

*PA What I'm saying, I remember there was, it was actually a staff who became an administrator who was a patient, as well, and then there were issues regarding should we allow him to see other patient's files or not and how much we should disclose. So I think that, because it could cause a team to start… to having split some… So you need to discuss that early; whether you allow the peer to have access to certain information and you have to, just because that could cause a problem.* (Staff – site 5)

**2.1.5 Clear feedback procedures (1 source, 2 references)**

One carer highlighted the importance of collecting feedback from PSWs about each visit.

**2.1.6 Clinical responsibility (6 sources, 17 references)**

A number of participants commented on the nature of the clinical responsibility of the PSW. Carers made suggestions for training for PSWs in areas of life support (Carer – site 3) and risk (Carer – site 1).Service users questioned which safety mechanisms would be in place for PSWs and established with an interviewer that risk assessments would be carried out by the CRT(Service user – site 4). Staff members raised concerns that PSWs might not have the clinical skills to deal with service users who express suicidal thoughts, and suggested that staff might need to be present:

*PA I think that if they’re going to do a peer group I think there should be a member of staff present, because my concern is that some people will say, I’m really suicidal, and I think that puts a lot of pressure on people with mental health problems who are going to have to mop up the pieces and that they might not actually have the skills or ability to deal with that.* (Staff – site 1)

Issus of clinical responsibility were also raised by staff, and a question was raised regarding the CRT’s duty of care with service users’ historical risk factors:

*PA Your duty of care is that you need to let them know that if there is a significant warning sign, a significant risk, then that's, even if it’s quite an old historical one, you want them to know that.* (Staff – site 2)

*PA It would probably have to be quite a full handover of the risk because if the worst happens with… as an organisation the organisation's going to be on pretty shaky ground, aren't they, if something happens? And say the, I don't know, something terrible like the peer gets assaulted by the patient and we haven't made them aware that there's a, what the risk was and they've, that's happened, that's going to be… isn't it?*

*[General agreement]* (Staff –site 5)

**2.2 General design of intervention considerations**

These considerations were related to how the programme is designed and how it fits with CRT services.

**2.2.1 Collaborative input (2 sources, 4 references)**

One carer and one member of staff referred to collaborative input between the service user, PSW and the CRT. Themes from carers related to empowerment of service users, increased service user motivation for the programme if they have been involved before the programme starts, and overcoming distrust and marginalisation by involving service user input:

*PA Yes. Also say, for example, this is going to go ahead, then if service users like we said before that they weren't too happy about it, if it is going to go ahead then to see actually how they would like it to be. Because they're going to be the ones who are going to be using it, and it's always good to go along with service users I think.*

*PA And I think that's really important that they feel part of it, because it could be seen as something implemented, and they've not no power, they feel disempowered. And with people with mental health issues often feel disempowered. So I very much agree with what you said earlier.*

*PA Empowers, yes, it's about empowering people.*

*(Carer – site 1)*

*PA They would have to be… I'm just saying that if service users had a big input, well then they're more likely to want to be involved, that's how I see it.* (Carer – site 1)

*PA And I agree with what you said about it's a trust issue, and the service users are crucial, they need to be involved at every stage, because as it they feel marginalised.* (Carer –site 1)

A member of staff commented that service users should have input into CRT work itself:

*PA But that’s the complaint that the service users can say, that when they ring up the crisis team, in crisis, we don’t give them what they want […] the time or the right advice, and I would very much like to advocate that that part of the crisis team work is managed by service users.*

(Staff – site 3)

**2.2.2 PSWs in CRT (5 sources, 11 references)**

Three members of staff and two service users highlighted issues regarding whether the PSWs should be embedded in CRTs. One member of staff suggested the benefit of embedding a PSW in a CRT in facilitating continuity across service provision; two service users also suggested that continuity would be beneficial and would help with information sharing:

*PA Because I think the worst part would be, I think for the person who’s referred to that service is they get to know, work with the crisis team and then they have to get to know and get to work with the peer support person, who might not be as informed as the crisis team are because they’re coming from a different perspective perhaps. Whereas if the peer support is already embedded in the crisis team and it’s just a slipping across kind of process, which will address a lot of those issues around risk and location…*

*(Staff – site 1)*

*But when you have a new people all the time, it makes you feel very confused and very distracted. The more you have the old nurses or crisis team, the better is for the future. Like how I have Jenny Morris, I met her on central [unclear], so I'm happy and glad being with her.*

*IV So it sounds a bit that you would like the continuity to be from the crisis team, perhaps, rather than having yet another worker come in. Is that right?*

*PA Yes*

*(Service user – site 1)*

*PA PSW could be involved in care by sharing info with MH staff (Service user – site 4)*

The other two members of staff pointed out that supervising PSWs can be time-consuming and there might be confusion surrounding whether the service user is discharged to the PSW. These could be disadvantages of embedding PSWs in the CRTs:

*PA Yes, but these were volunteers, but they were ex service users. I’m using that model because they’re ex-service users and what they can, you know, cope with. I mean, I had… I had four at a time I was working with, and it’s… they… you need to have someone with them all the time. That’s what the experience I had.*

*(Staff – site 4)*

*PA Because the client is discharged to… who would we discharge the client to? To the peer? Who is the client discharged to?* (Staff – site 4)

**2.2.3 Separation of PSWs from CRT (7 sources, 24 references)**

Two service users, one carer and four members of staff made suggestions about whether PSWs should be separate from CRTs. The service users agreed that the PSW should be employed by the trust (Service user – site 1) and asked whether PSWs will be able to signpost to other services (Service user – site 4).

One carer recommended that it is made clear to participants that the PSW is not a trained mental health worker, and this could require some separation from the CRT:

*PA And I presuming that it would be made clear to the person who’s going to receive them that they’re not trained as a mental health worker because they’re not going to look any different, are they?*

(Carer – site 3)

Four members of staff agreed that the PSW should be kept separate from psychiatric work; separation from the CRT would be helpful for bureaucracy of the programme; separation would help to avoid over-involvement from the CRT:

*PA Maybe it needs an element of independence in order for it to be a peer support group so it doesn’t feel like it’s another kind of piece of psychiatric work. It’s something a little bit different. Give it a little… some autonomy.*

*(Staff – site 1)*

*PA My worry is… so, my only thing is that if you attach peer people with home treatment team, it’s going to fail in many respects, in terms of protocols, in terms of access issues, in terms of this peer person getting involved in the different business of the home treatment team. So, they have to be separated from home treatment team. I don’t know which is the best place for them to sit, but anything out of NELFT, either in primary care, either in Mind, either in some sort of voluntary organisation will be much better. And then NELFT giving that, sort of, budget to this organisation saying this is the budget, this is what is the expected outcome from you. You go and do it.*

(staff – site 4)

*PA Yes, that's a different responsibility and expectations; we're then saying teams will be involved in maybe explaining what peer support is and if there's a problem in the relationship then we might say no, we're actually going to the meeting, keep an eye. We might come into another one which we should have... the patient says it would be good if you were there, it would help our relationship. So you don't get sucked into all of that, no. I think it’s best to have no involvement.* (Staff – site 2)

Staff also commented that separation from the CRT could help to clarify issues of discharge and responsibility for the service user (Staff – site 4)

**2.2.4 Continuity of PSW (3 sources, 7 references)**

One member of staff highlighted a benefit of having one continuous PSW per client that the client wouldn’t be required to ‘repeat the story over and over again’ (Staff – site 1) Two service users commented that having the same PSW throughout the programme would be beneficial and asked whether PSWs could be employed for longer:

*IV It will be one person who will stay with them, it is not the same as the Crisis Team who might send you a different person every day. There is the continuity in that piece of work.*

*PA Well, but that’s a good thing.*

(Service user – site 4)

*PA Because everybody’s situation is different. What I was thinking was you might discover some people are really, really good at this job and you should have a way, if they’re going to stay longer, to keep them because experience make people even better, better people even better. You should keep that somewhere in your mind because you don’t want to let go people who want to stay.* (Service user – site 4)

**2.2.5 Case load size (1 source, 1 reference)**

One member of staff raised concerns that the case load size of service users per peer is ‘not too onerous’ (Staff – site 2)

**2.2.6 Back-up workbooks (1 source, 1 reference)**

One service user suggested that the PSW could keep a back-up copy of the participant’s completed workbook:

*PA I think that would be a good idea because the thing is, I think that if there’s two booklets and then you’ve got the service user filling it out for themselves, sometimes, you know, they have learning needs and dyslexia and stuff, they can’t fill it out themselves, and also sometimes the service user will lose it and then, you know, it will be hard… it would be good if they can… the support worker can make, take a copy of it or have a… fill out a copy of it for themselves and file it away…*

*IV That’s a good idea, yes.*

*PA With their notes, so in a crisis situation they will be able to get that again.*

(Service user – site 4)

**3 Commencement**

Participants in the focus groups commented on potential barriers to the commencement of the PSW programme, and issues that could potentially arise following commencement.

**3.1 Potential barriers to commencement**

**3.1.1 Desire to move on from services (5 sources, 9 references)**

Carers, service users and staff highlighted the potential for service users to want to move on from mental health services, in which case having a PSW might not be beneficial. Two service users pointed out ‘some people, they’re very independent, they don’t want any help from anybody’ (Service user – site 4) and ; And when I'm not in crisis, I don't really need anybody because, once I'm well, I can think to myself, oh, yes, you can do that’ (Service user – site 1)

A carer suggested that some service users might recognise a stigma of mental illness and might not want to associate with other mental health service users:

*PA They won't mix. So we can go down the line of having peer, and they can then be a role model, that is good, but a lot of them won't mix. They won't mix because they don't want to be seen with other mental health patients socialising out in public, because that is a stigma, a huge big stigma in our society of having a mental illness. You've got half a dozen mental ill patients walking down the street, having a night out, can you imagine what it'll be like? It would be terrible, the reaction that they would get. So a lot of them know this, they're not stupid, they just don't want to be seen with other mental health patients.*

*I've known a case in hospital which a user actually in a group I was in said that she was unwell, she was in hospital, in the mental hospital, and when she was unwell it wasn't apparent to her, but as she started recovering, she noticed all around her people being very disturbing and that made her even worse. So they don't want to be around other mental health patients that can be disturbing, even if there are recovered, their opinions and their way of dealing with things may not be right for that person…*(Carer – site 1)

Two members of staff similarly cited a recovery process of ‘normalisation’ and ‘getting people away from anything to do with mental health services’ (staff – site 4); and service users wanting to ‘just get on with their lives’ (staff – site 2).

**3.1 2 Professional preferred (2 sources, 3 references)**

One carer questioned whether the PSW programme would be suitable for service users at a post-crisis stage, suggesting that further professional input could be more appropriate:

*PA I think a lot of the people we're dealing with are young men, maybe in the 20 to 30, 35, age group. There's a huge amount of peer pressure. They come out of crisis, as I say, the difference between I'm in crisis, the team's looking after me, I'm now out of crisis, is very small, you'd be very lucky, I think they feel they can make a judgement that suicide is not prevalent, they're not going to harm somebody else, they don't need to be committed. Continuity is really important. I think an eight to 10 week intervention is probably too short. I don't think post-crisis is necessarily the place to put this. I think post-crisis you need a very good professional input to move someone down that next track, if they're going to go there, that is currently missing, that does not exist.*(Carer – site 1)

A service user commented that they would not need a PSW as well as the support they already have:

*IV […] if you had a worker who had those kinds of problems themselves, just wondering whether you think they would be helpful to you or less helpful than a purely professional worker? I'm asking because it's possible that you might think they were more helpful because they’ve been through it themselves, or you might have different views. So do you think... just be useful to know whether you think they would be helpful to have as well, or...?*

*PA I don't because my carer, Jenny, psychiatric nurse, either my sister or I phone her when I'm in crisis, and often she comes round.*

(Service user – site 1)

**3.1.3 Confidentiality concerns (6 sources, 7 references)**

Service users and staff raised concerns regarding the PSW’s access to and response to confidential information. Four service users made comments regarding how closely-knit the mental health community is and how that could add to confidentiality issues; concerns at what a PSW might tell others about the participant; and limits to confidentiality such as the PSW needing to break confidentiality if a participant discloses suicide plans:

*PA […] I was involved for three years teaching deaf children at Frank Barnes Deaf School, and the deaf people will tell you that if they have a problem, they prefer not to bring it to the deaf community. They prefer to take it to the hearing community because it's such a close knit community. So I would have reservations about having someone that's a service user that's not employed by the Trust, who is co-signatory to the confidentiality and all the other stuff.* (Service user – site 1)

*PA What about if… because obviously in the mental health network we know a lot of people, a lot of people know other people. What if the peer was actually someone that you knew, like a friend or something, would you be able to say I don’t want to see this person, can I have someone else?*(Service user – site 4)

*PA No, well in a way, still it depends. It depends, confidentiality is a big out there, but it depends. It depends on what you’re going to say, and what you’re going to do, but you have to...it has to be, most of it has to be confidential, you know? Because, I don’t want no one going out there and saying things about me that is not right. Yes, that is not true, so confidentiality comes into it as well. If I’m honest.*(Service user – site 2)

*IV But if it was somebody else with experience, would you worry about them breaching your confidentiality, breaching your confidence?*

*PA No, I don’t think I would because if I told someone something and obviously they were worried and told someone else, then I know that… no. Well, at the time I might be angry but in the long run I’d know they were doing the right thing because they’ve probably been in the same situation as as I’m in then. So I think it would be good for them to tell someone that, you know… I mean, obviously if I told them that I’m feeling really suicidal or something but don’t tell anyone, and then I went and done something then they’re going to feel really bad, so something like that I feel they should then tell someone, yes.* (Service user –site 3)

Three staff members’ concerns were related to whether the PSW would have access to service user files and how that would be managed (Staff – sites 3 and 5)

**3.2 Commencement considerations**

**3.2.1 Wellness of SU (5 sources, 8 references)**

Service users, carers and staff members raised concerns about the wellness of participants. These particularly related to the recovery stage that the participant would be in immediately following their discharge from a CRT. A service user suggested that participants might not be in the right stage of recovery to take part:

*PA1 Have they taken… they haven’t even taken into consideration the fact that as soon as you come out of hospital or a crisis group, the fact that it’s not a call, some people… you’re not well enough to even study, right…*

*PA2 Exactly.*

*PA1 So how are you going to do ten sessions? Sometimes maybe you won’t even feel like doing anything for weeks, days, months, you know, yes, that’s the thing.* (Service user – site 4)

A carer pointed out the vulnerability of a potential participant:

*PA […] after crisis team, the person is still on a very vulnerable stage when they won't recognise any role model around them, maybe peer support could be organised for different stages. For example, one stage could be after the crisis team, or not instead but on a level with the early intervention team*(Carer – site 1)

Two members of staff suggested that the participants’ symptoms could be too much for the PSW; and some service users are discharged because of non-compliance rather than being ready for recovery:

*PA there are a lot of issues around what sort of clients those peer workers can work with, because, you know, we work in crisis teams, the clients we discharge go to CRTs. It’s like we said, the clients who can be discharged from CRTs, so, they are the other ones who are going back to primary care, or, simply to GPs which are essentially symptom-free as well for a space of time. The ones we are working with remain symptom… they may have symptoms of psychosis, mental illness, [unclear], so, I think that’s key, but, you know and what are the expectations of the… of the clients themselves? What do they want to achieve in those eight to ten sessions? […] I think it might be too much for a peer person, to work with patient who is crisis with [unclear].*

(Staff – site 4)

*PA Would all our clients be offered the option of peer support or would it be to choose? Because sometimes our clients who are discharged, we're not discharging them because they're well, we're discharging them because they're refusing to work with us, they're not taking medication. And the understanding is that they're possibly going to become more unwell and there will be an intervention that can maybe help, that session. So in that case they probably wouldn't suitable at all for peer support going forward, so would it become that it’s everyone who gets offered it?*

(Staff – site 2)

**3.2.2 SU risk (5 sources, 18 references)**

Carers, service users and staff commented on the risk that participants could pose to the PSW. A service user agreed that it would be a ’good idea’ to have an option for PSW meetings with the participant in NHS premises (Service user – site 4) A carer suggested that the PSW should not meet with a participant if it is not safe for them to do so:

*PA It has to be safe, but I think also one of the things that I would get to that if the person with the mental health issues is not currently considered safe, then they're not suitable for peer support. I think that ought to be very clear, there is no way that somebody should be put in danger on either side, just because they can't cope.*(Carer – site 1)

Staff members highlighted the need for the PSW to be trained in handling violent situations; the higher risk of PSWs meeting participants at home; the need for updated risk assessments; a suggestion that PSWs meet with low risk participants only; and the need for PSWs to contact someone if there is a safety issue:

*PA There's a potential risk associated, such as what the training would be [inaudible] and how does the client choose, who's responsible for choosing them, and who’s responsible for, they've chosen them and then there's an incident either with a client or their support worker and how does the person manage the relationships, if there's an actual violent incident or something of that sort, how do you manage the relationship that might form between them if they're not experienced in doing that.(Staff – site 2)*

*IV What about in terms of where they decide to meet, so people meet in homes or people meet on NHS grounds? What do people feel about that, from your experiences of working with these people?*

*PA1 I think there’d be quite a few risks involved in the client in their homes. It would be better conducted in some...*

*PA2 Neutral...*

*PA1 ... hospital, yeah, a neutral setting.*

*PA2 ... space, yeah.*

(Staff – site 2)

*PA They said the most dangerous thing about doing risk assessment is by just referring to when you do it on the Friday 11th December? Because that's only relevant to Friday 11th December, and so unless you review it, it’s worthless. So you have to be constantly reviewing it and it’s a long period of time. It’s not just a few days.*

(Staff – site 2)

*PA I just wanted to ask the team, we wouldn't be best placed for the background risk assessment because the risk assessment we do, each clinician is constantly reviewing it and every visit it’s reviewed and handovers and having to deal with that. So it’s quite a live thing, but I've done some risk assessment replacements a couple of years ago and found it was a very, very different thing and you do feel a lot more responsibility because you know that whether this person gets this flat or gets this accommodation could weigh quite heavily on this risk assessment. And I've found there's a different style of thinking to the kind of risk assessment that we do in the team, because we're very much about what it’s about now. With that one you would also be a lot about the historical as well.*

(Staff – site 2)

*PA I presume not everybody is suitable for this service and maybe someone where they can identify, maybe allocate case notes. You don't just allocate someone, the referral, to anybody; you screen them, you identify them and these factors whether they're suitable or not. And I presume for the peers, anybody to be allocated to a peer I presume they would be really low risk patient. There is no point allocating somebody who is high risk. Even if you tell me a whole array of their risk history; if they are still risky if I am a peer my safety would be compromised, as well. So maybe it would be suitable for lower risk, very low risk patients.*

(Staff – site 5)

*PA Because you've got to keep the peers safe, as well. [Overtalking]. And the Trust responsibilities, so part of the peer contract would be sign that, on the dotted line that if there are issues then they should contact someone immediately. (Staff – site 5)*

**3.2.3 SU full understanding of programme (3 sources, 4 references)**

One carer and three members of staff commented on whether the service user would have a full understanding of the PSW programme. The carer stressed the need for clarity about the time limits of each PSW session:

*PA1 And to know that the end is coming up in five, 10 minutes, and to wind down.*

*IV So strict time limits.*

*PA2 Well, you could almost say, we’ve got an hour today and this is your time, what do you want to go through, and then, yes, so you know at the beginning that…*

*PA3 Your time limit, yes.*

*PA1 Because people who are really desperate to have company will try to hang on to you. You have to have the boundary of now.(Carer – site 3)*

The staff members referred to the importance of explaining the programme to the participant; signposting to other services; and a disadvantage of the programme in the participants might not have a full understanding of it:

*IV Before it starts.*

*PA1 That should be when the service is being explained to them. I think it should be really clear what...*

*PA2 And how long it’s going to be provided for as well.*

*PA1 And maybe the peer support should have a little more information about other services that are there, just do the sign postings, because with some clients inevitably the first thing they're going to talk about always is those issues. And you have to get past that to move on. So it’s about knowing that, well, as you've mentioned before, we need to [inaudible].*

(Staff – site 2)

*PA [Overtalking] clinical information to non clinicians. At the start of the relationship a client might not have a very good understanding of what peer support is and I don't feel comfortable with that actually.*

(Staff – site 2)

**3.2.4 Workbook usage challenges (3 sources, 6 references)**

Three service users and a carer highlighted possible challenges to completing the workbook. The carer mentioned that the participants might not be well enough to read the workbook:

*PA Because I think also with the book, it’s all well and good if you can read, though what I found with my husband thing is that’s how we knew he was getting ill was when he stopped being able to read, and we were given all this information from the crisis team and [unclear] couldn’t have read it. He’s [unclear]. He just can’t. So I don’t know how helpful that would be, if that makes sense.*

(Carer – site 3)

Two service users raised concerns about the possibility that the participant could lose the workbook (Service user – site 4). The third service user commented that the use of a workbook is ‘impersonal’ and they would prefer to talk about ‘life in general’ with a PSW(Service user – site 3)

**3.2.5 Choice of peer (4 sources, 7 references)**

One service user, one carer and two members of staff made comments related to how the PSW would be chosen for each participant. The service user commented that they would want to pick their own PSW, to make sure that they have a similar experience:

*IV […] Do you think that having somebody who’s had experience in mental health services, coming to see you once the crisis team [overtalking].*

*PA Only if you pick your own person. […] I want someone I know in this hospital. If you could… If I’m allowed to write the name down I’d even pay for him to come and see me once a week.*

*IV Okay. So why would you want to choose somebody?*

*PA Because he’s been through the system. He’s been locked up for six months in one of these.*

*IV Yes, okay.*

*PA [Unclear].*

*IV Okay, so it is about…*

*PA Personal choice.*

*IV You want somebody who’s had similar experiences to you and you want to be able to choose, say yes or no whether that’s somebody that you feel comfortable with.*

*PA Yes.*

(Service user – site 3)

One carer recommended flexibility in allowing participants to change PSWs if they ‘didn’t gel’ with the PSW they were assigned (Carer – site 3)

Two members of staff asked for clarification on how PSWs would be matched to participants (Staff – site 4) and who would be responsible for the risks of choosing the PSW (Staff – site 2)

**3.2.6 Matching – positive (5 sources, 9 references)**

Three carers, two service users and one staff member expressed positive views on matching PSWs to participants. One carer asked whether PSWs and participants would be matched by ‘same age group or the same likes and interests?’ (Carer – site 3) The other two carers recommended that PSWs could be matched to participants who have the same diagnosis (Carer – site 1) or the same mental illness:

*PA I've got another question, sorry. Is person who, or people, who did experience mental health issues before, are they going to count as recovered people, or people still? Another question is I think depends from what type of mental problem people experience, they may be able to help or understand others, they may not. Because this could be paranoid, or schizophrenic, but it could be other issues which people experience. So understanding of someone experienced the problem to degrees, but understanding deeper levels of mental issue is not necessary. So you want to place it, not you personally, but someone like to place the person who is unqualified recognise the problem, understand, going to come and try to help, is that…*(Carer – site 1)

Service users similarly recommended that PSWs be matched with participants who have the ‘same problems’, but also pointed out the difficulty of matching for the same symptoms:

*PA I was diagnosed quite late in life, unfortunately, so I missed out on a lot of schooling so I’m thick as anything, but, like, it would have been… It would be good to, like, talk to other people who have got not just ADHD but borderline personality disorder as well, because the only other person I know that’s got it is my sister Katherine [?] and a guy I met in prison, so I’ve never really spoke to anyone about it and stuff like that, and what are the side effects and stuff like that.*

*IV Okay, that’s… So talking to somebody that has the same problems that you’ve experienced is an important thing?*

(Service user – site 3)

*PA Well, I was thinking what about the actual twinning of the people? I mean, will consideration be given to what the person was suffering from, because it’s true that… it’s very true that, you know, why service user involvement is recognised in many areas is that mental illness is a subjective condition, and so that’s why service user involvement is seen as being necessary for planning services as well as helping care for people. But having said all that, it’s a very… you know, mental health is a very umbrella area, so you’ll have people with depression, you’ll have people with personality disorders who are very tricky, and then you’ll have people with positive and negative symptoms of schizophrenia, so if there’s going to be any sort of expertise at all I think careful thought should be given to twinning people. […] Yes, maybe not, you know, completely down the line one for one, but I think it’s an important consideration because really different conditions are completely different.*(Service user – site 4)

A member of staff likewise agreed with the importance of matching PSWs to participants:

*IV So there’s a sense that, actually, you think it’s a good idea; it needs to be done carefully; the matching of people by diagnosis, you’re saying, is… there’s a sense that that’s important?*

*PA You’d have to give it great consideration. Like you said, you couldn’t have a borderline personality disorder talk to somebody who suffers from schizophrenia or…*

(Staff – site 3)

**3.2.7 Matching – negative (5 sources, 5 references)**

Two service users, two carers and one member of staff gave reasons for not matching PSWs and participants. The service users suggested that people with a similar diagnosis such as schizophrenia might not be helpful for others with that diagnosis; and it might be preferable to have a PSW who has different experiences:

*PA If he’s schizophrenic, he’ll say, well, send a schizophrenic round, what’s the good of that? That’d be no good whatsoever. There’d be so many voices there you wouldn’t put the radio on, would you?*

(Service user – site 3)

*PA Yes, I can see the advantage of that for some people who feel it’s like another level of connection where someone could share experience of a diagnosis, but in some ways just to be able to be with somebody who’s there for them can be more important than whether it’s somebody with the exact match. And I think when I was an inpatient [unclear] we have different diagnoses it’s almost as if at some level the stress was really the same. It was about not being able to function in an environment that wasn’t safe really, so it was almost that connection that became much more important than the slight differences between people’s different diagnoses. So for me I would... It’s also you learn different things from different people’s experiences, so I would almost prefer somebody who didn’t have something that was exactly the same as what I have because it’s quite nice to know that you can have had completely different experiences and yet still be able to support each other – as long as it’s somebody who’d been through that process of feeling vulnerable and know what to need support is like and to be suffering from mental health rather than physical health and the difference between that. I think that’s really the key bit for me anyway rather than needing to be matched. (Service user – site 5)*

The carers similarly suggested that generic similarities between people who have experienced mental health problems could be useful; and a holistic view of people without focussing on matching exact diagnoses could be beneficial:

*PA I actually think now, I came in thinking there should be matched diagnosis, but I think many of the things that are there are generic, there are very, very big issue for people with mental health. And it's like buying the trainers, hundreds of us could have that experience and they would know how wonderful it was to go out, have your own money, actually go into the shop, not have the ones your mum chose, which were naff, but have the really flashy ones that you have fancied for weeks, and they're yours. I think may mental health people could share those issues without being the problems that are maybe this big, in comparison to being a recovering person from mental health.*(Carer – site 1)

*PA Sorry, the other thing I will say is to look at the person holistically, not to look at the condition, and identify them by their condition, because they want… they're human beings first and foremost, they have an illness. So look at the person as a whole, and also not just focusing on their mental health, focus on their physical health, their whole self.*(Carer – site 1)

A member of staff added that there might be difficulties in the employment practicalities if matching PSWs with participants with the same diagnosis:

*PA But if they are employed by the trust, you can’t really [unclear] try to make the nest needs [unclear], but they can’t choose, it’s, like, if when a team and the people were coming to visit the service user cannot choose and say I want this staff, and I want that one. You can try to meet their needs, but if they’re employed by the trust, really, it’s tough really to try to meet individual needs. We can try to look at really what are the general needs really, but it’s too great [?] if you’ve got to try to match this service user and a peer to say this is the best person. You know, we try to do that, but if they’re employed, they’re employed, we have to work with what we’ve got. And we’re all employed, we might be the best person for the service user, but we’re not. We still have to deliver [unclear], so…*(Staff – site 4)

**3.2.8 Boundaries (3 sources, 7 references)**

Two staff members and a service user made comments about the boundaries that should be adhered to by PSWs and/or participants. The service user recommended that PSWs receive training and suggested boundary issues could revolve around the amount of contact between the PSW and participant:

*PA Practical things, really. Like, making sure that person has got boundaries with you, and things like that? Which hopefully would have been part of their training, to understand about boundaries and things like that, and...*

*[…]*

*IV What might be a problem around boundaries, if boundaries weren’t kept?*

*PA God, you ringing your mentor up all the time, or them ringing you all the time, and them seeing you more than they should be doing during the week, and things like that, you know? It’s just sticking to everything really, I suppose, you know?*

(Service user – site 2)

The staff members referred to ‘proper boundaries’ (Staff – site 4) and cited some potential boundary issues regarding relationships between PSWs and participants, and confusion with care coordinator roles:

*PA Because they’re doing a lot of one-to-one, and trying to spend more time with all the service users, because there are issues around their boundaries, and really how they get their supported [?]. There may be issues, maybe like, sometimes, when people want… maybe, like, maybe why we don’t have maybe, like, a [unclear] to protect where there are actually boundaries [unclear] really. We end up having, maybe, God forbid, maybe [unclear], end up having a relationship happening [unclear], but sometimes is really because they are also vulnerable themselves. But really it’s about how much, like, Michael said, I think… I think there has to be a lot around how the peer themselves, the peer support team they are to be protected and supported. And there has to be really a lot around really how we would support that. (Staff – site 4)*

*PA1 It’s whether they, as well as risk, if people start seeing them as a pseudo care coordinator when they're not getting one, and it’s to get the patient, what their abilities are, sort out benefits and...*

*IV So it’s important you think for the peer not to get drawn into that?*

*PA1 Yes. I mean, we can't sort those things out as professionals; it’s hard to sort lots of things out.*

*PA2 Have a very clear boundary.*

*PA3 Boundary, yeah.*

*PA1 It is non-negotiable.(Staff – site 2)*

**3.2.9 PSW – paid employment (1 source, 1 reference)**

One member of staff recommended that PSWs should be employed and paid by the Trusts (Staff – site 4)

**3.2.10 Minimising CRT staff time commitment (1 source, 1 reference)**

One member of staff raised concerns about CRT staff time needed to oversee the PSWs, and mentioned the potential for the CRT to have to meet ‘the immediate crisis needs of the team’ (Staff – site 4)

**3.2.11 Discharge timing – Positive (5 sources, 9 references)**

Three service users, one carer and one staff member stated that starting a PSW programme shortly after or overlapping with discharge would be positive in terms of timing. Two service users stressed the importance of continuity between services (Service users – sites 1 and 2), and two service users recommended an overlap between CRT care and the PSW programme(Service users – sites 3 and 5). The carer similarly cited continuity as a priority in the timing of the PSW programme soon after discharge (Carer – site 3). The member of staff referred to the disadvantageous effects of a gap between discharge from the CRT and the start of other service care, and suggested that the PSW programme or community support could help to fill this gap (Staff – site 4)

**3.2.12 Discharge timing – Negative (4 sources, 6 references)**

Two staff member, one service user and one carer made comments relating to the negative aspects of staring the PSW after CRT discharge. These four participants all commented that the PSW participants might not be well enough after discharge to participate; and one member of staff additionally suggested that the PSW programme should be run concurrently with CRT care:

*PA1 Have they taken… they haven’t even taken into consideration the fact that as soon as you come out of hospital or a crisis group, the fact that it’s not a call, some people… you’re not well enough to even study, right…*

*PA2 Exactly.*

*PA1 So how are you going to do ten sessions? Sometimes maybe you won’t even feel like doing anything for weeks, days, months, you know, yes, that’s the thing.*

(Service user – site 4)

*PA I don't think post-crisis is necessarily the place to put this. I think post-crisis you need a very good professional input to move someone down that next track, if they're going to go there, that is currently missing, that does not exist.* [*<CI_Carer_04.04.2012>*](file:///C:\Users\rejukfu\AppData\Local\Microsoft\Windows\Temporary%20Internet%20Files\Content.Outlook\55ZWPWJ7\1f6fea73-fba7-449e-a8d0-237f5e7a2f2d)

*PA1 I mean, there are clients that are [unclear], and when we discharge them they might not be ready [?] for this, kind of, support.*

*PA2 […]They have to start to want discharge, or, can the peer support group say, the peer support person actually work together with HTT from day one the person is taken on, they are able to engage with that, whereas we are doing both at the same time, because I think, what I struggle with [unclear] eight sessions, ten sessions whatever number of sessions I think is really where you have got this HTT done and then towards somewhere you start another intervention to [unclear]. If it was actually there was that possibility of actually doing everything together, where if you’ve taken on someone and you can really… what they… how far they are able to [unclear]…*

(Staff – site 4)

*PA I think the timing of the [overtalking] after discharge from the Crisis Resolution Team is perhaps too early, because people are not 100% well. I mean they can't... we get maybe 5% who are reasonably well but they're not going to stay well, it would be 90%. So I’m just wondering about the timing of it, to work it after the Community Mental Health Team knows [?] and when they're discharged. Because I think there might be just that bit too [inaudible].*(Staff – site 2)

**4 Content and delivery**

**3.4.1 Mode of delivery**

**4.1.1 Flexibility of intervention (7 sources, 10 references)**

Carers, service users and staff members commented on the flexibility of the PSW programme. Of the two service users, one suggested that the mode of intervention (such as a set booklet) would not make a difference as long as they are told about the ‘plan’ and arrangements are made in advance (Service user – site 2). The other service user recommended a combination of group and one-to-one sessions as part of the PSW programme (Service user – site 5).

Three carers discussed the advantages and disadvantages of alternative modes of technology to present parts of the PSW programme. Suggestions were that some people enjoy using computers but it’s not practical for others; texts or Facebook can help to get people interested; and smartphone apps can help to show change in recovery over a period of time (Carer – site 3)

Staff commented on a disadvantage of the format of ‘two people having a cup of tea and talking’ being the participant ‘left with nothing and nothing’s really been achieved’ by the end of the programme; and recommended flexibility of peer support but with ‘a bit of structure’ (Staff – site 1) Other staff commented that reminders by computer can be useful for some, but not all, participants (Staff – site 2 and 5)

**4.1.2 One to one preferred (3 sources, 8 references)**

One carer and three service users stated a preference for 1 to 1 PSW input. The carer mentioned advantages of 1 to 1 support being easier tailoring for the individual, and better continuity from crisis support (Carer – site 3). Service users’ preference for 1 to 1 support was based on discomfort in groups; advantages of intensity of feedback and building a relationship;

*PA1 On a one to one basis, because sometimes I’m not one for a lot of people around me, so I would say one to one basis.*

*[…]*

*PA2 I agree, on a one to one would be much better than a group, because you’ll get more feedback from him or her individually, rather than you would do in a group, I think. Obviously, you get more feedback from other people, but you know, if you want to have that relationship with your peer support worker, then I think a one to one probably would be better for me, and if...because if I want to go to a group, I can just go and find a group, and go and join a group. Which would be a little bit extra maybe support, but yes, on a one to one basis, I think would be much more suitable for me.*

(Service user – site 2)

*PA I find that sometimes, if I am quite distressed, having... it sounds crude, but having two against one can be... I find myself being less open or less trusting than if it’s just one on one – that’s a personal thing. That’s more in terms of I can imagine, if it was somebody who was very new to taking on the role of a peer support worker, to go out and to see people on their own one-on-one might be a challenge, whereas if it was somebody who was quite comfortable and quite used to it, I think one-on-one is probably better. (Service user – site 5)*

**4.1.3 Bite size intervention (3 sources, 4 references)**

A service user and a carer recommended that the PSW intervention be delivered in bite sized pieces. The carer suggested that this is necessary because of participants’ potential concentration problems (Carer – site 3). The service user gave an example of ‘little goals’ that the PSW could help with:

*PA To move forward, and they’ll know what’s manageable and not manageable, because they’ll have mental health problems themselves. So, they’re not going to say to you, go and get a job, do you know what I mean, just like other people. Go and do voluntary work or something, just once a week or something, just to get out in the community and do something.(Service user – site 2)*

**4.1.4 Computers – technology (7 sources, 8 references)**

A service user described the use of technology as ‘impersonal’(Service user – site 3). In contrast, another service user suggested that smartphones could be comfortably used by younger participants:

*PA I think there are some days or some periods where the more that you can be doing and interacting the better. So for me something web-based... I myself don’t use a smart phone, but I think if I did, having something that was in my pocket, that wherever I was I could have a log in and just note on the bus, feeling very stressed out, then let it go. That might be really helpful. I think for younger people it’s fairly comfortable to use those kinds of things. It’s probably not... I know my grandmother wouldn't, but it’s fairly... Using internet and using smart phones is second nature, so it would be really good to have that option. Yes, I think it’s a really good idea. (Service user – site 5)*

Carers, service users and staff members commented on the flexibility of the PSW programme. Of the two service users, one suggested that the mode of intervention (such as a set booklet) would not make a difference as long as they are told about the ‘plan’ and arrangements are made in advance *(Service user – site 2)*The other service user recommended a combination of group and one-to-one sessions as part of the PSW programme *(Service user – site 5).*

Three carers discussed the advantages and disadvantages of alternative modes of technology to present parts of the PSW programme. Suggestions were that some people enjoy using computers but it’s not practical for others; texts or Facebook can help to get people interested; and smartphone apps can help to show change in recovery over a period of time (Carer – site 3).

Staff commented on a disadvantage of the format of ‘two people having a cup of tea and talking’ being the participant ‘left with nothing and nothing’s really been achieved’ by the end of the programme; and recommended flexibility of peer support but with ‘a bit of structure’ (Staff – site 1). Other staff commented that reminders by computer can be useful for some, but not all, participants (Staff – site 2 and site 5)

**4.1.5 Structured manual (5 sources, 8 references)**

Carers, service users and staff commented on a structured manual for the PSW programme. Carers showed mixed views towards how useful a structured manual would be:

*PA Plan and structure, and outlines, have to be written somewhere as a question and answer site, or online would be very helpful for both end user and carers.(Carer – site 1)*

*PA […] we got a paper-based packet at home, which is great. I find it helpful having it, but my children keep finding it. It’s got drawings all over it.*

*[…]*

*PA We’ve got a paper thing that we were given by the crisis team. It’s meant for us to fill in ourselves as a record, and Tim’s not felt able to do any of it, and I was doing it diligently, but I’m afraid I’ve stopped now, but having some kind of record… and we’ve got a care plan, as well… but we almost feel it’s a bit unnecessary. We don’t really need it, and Tim’s never looked at it, but it’s useful to know what’s been written about us.* (Carer – site 3)

Service users recommended have a structured way of keeping PSW appointments, for example a wall chart (Service user – site 2), and the advantages for memory of having a written manual:

*PA I think it’s a good idea, like, workbooks or computers or whatever your preference is really. I think it depends, but I find that if I’ve got something that I can read through and work with, then it’s a lot easier for me because, like, if people tell me stuff, five minutes later I’ll forget it because my memory’s absolutely terrible, so I would like to, like, do things, like, on paper and, like you said, write stuff down and I find it a lot easier when you write it down.*(Service user – site 3)

One staff member was uncertain about how the structured manual would be delivered.

*PA I suppose they've got things like their resource or their appropriate... they're quite not formulaic but they're quite clear things that you could work through, I think; if one is going to think of anything that peers could perhaps be doing with them, it might be things, that sounds reasonable. I'm not quite sure how you would do that for eight to ten weeks. (Staff – site 2)*

**4.1.6 Group benefits (4 sources, 5 references)**

Carers and service users spoke of potential benefits of groups. One carer recommended a group ‘you could dip in and out of as you need it’(Carer – site 3). A second carer provided examples of ways that groups could be helpful, and a third commented on the benefits of having a group at the end of the PSW programme:

*PA And acknowledging in every session, acknowledging a presence and acknowledging them as people in their own right, with their own skills, hidden skills that they haven't discovered that they will be sharing in the groups. Like one way in the groups getting to know each other, know how they cope with their carers, something like that, and lighting them to come out, they will be proud to talk about things like that, how did they cope and stuff like that.*

[*(Carer*](file:///C:\Users\rejukfu\AppData\Local\Microsoft\Windows\Temporary%20Internet%20Files\Content.Outlook\55ZWPWJ7\1f6fea73-fba7-449e-a8d0-237f5e7a2f2d) *– site 1)*

*PA I don’t know if there’s… sorry, I interrupted you. I was just wondering because going back to the original question about how long it should go on for, whether the peer support, if there was any willing volunteers to run a kind of group to see if there’s much call for it so that they can go and meet other peers and meet others going through the same kind of things because I know I found the carers’ one really helpful. Whether you could actually get them to go to it and whether there’s much demand, I don’t know.*

*IV For the clients?*

*PA Yes, for the clients. Say, once they come to the end of it, there’s someone… they can meet other people that have all got the same problem, because I found the best support from those that have been there themselves.*(Carer – site 3)

Service users gave examples of group benefits; and recommended that a group be held at the end of the 1 to 1 PSW programme:

*PA Another one thing for NHS they should do, that to bring a group of people once a month, like today, how it is now to talk, to encourage each other. Is very good thing, like today because we listen one another and we encourage one another. So you’ll be helpful; you’ll be helpful, the services or crisis things, psychiatric doctors and many other examples from us people. They could learn good things another, not only us.(Service user – site 1)*

*PA I suppose ideally a mixture. I’m not sure how the coordinating of a group might necessarily work for everybody compared with somebody... I don’t know. From my experience with the Crisis team of somebody visiting your house and that’s what you arrange, I’m not sure how a number of people together would coordinate that, but I think keeping in touch with other people who are doing the same thing can be really good and it’s perhaps not what you need immediately if you’re first using this. I think group only would be quite difficult but, yes, the combination might be really good, especially towards the end when it starts to become something you’re more comfortable with, something towards the end of the ten weeks, yes. […] I certainly think what might help with the feeling that it’s an ending, if that was to worry anyone, would be to experience a group situation where there were other people – not in terms of needing to make friends and connection. Just to feel supported in a group sense I think is very different from when it’s two people working together, so if session... week nine and ten happened with other people, I think that would ease the feeling of it coming to an end somehow. I think also from the beginning to know these are what the ten might look like. We might discuss roughly these things on these weeks, so there’s not an abrupt, oh by the way this is the last meeting, and you perhaps didn’t know it was going to be. I think when you know it’s coming and it’s been discussed all along, I think that can really help. Well, it certainly helps me. (Service user – site 1)*

**4.2 Length of delivery**

**4.2.1 Flexible number of sessions (5 sources, 9 references)**

Carers, staff and a service user made reference to flexibility in the number of sessions in the PSW programme. Carers suggested that the number of sessions needed might differ between participants:

*PA1 I think each person will be individual because one person might only need, say, about five sessions and one might need 10 sessions, and someone might need sessions for the rest of their life.*

*PA2 Yes, and I think if you say, we’re here as long as you need us, then actually they’ll probably need you for less time than if they know they’ve got to get it all in.*(Carer – site 3)

Another carer suggested that more sessions might be needed for participants and PSWs who ‘are finding […] less language which they can communicate on’ (Carer – site 1)

A service user highlighted the possibility that a participant could ‘feel better and then you say, well that’s enough after four [sessions]’ (Service user – site 3)

Staff members recommended trying to ‘find a balance’ in the number of sessions given (Staff – site 5); and recommended flexibility in the number of sessions depending on the needs of the participant:

*PA1 It comes back to the same issue of eight to ten weeks. Is that enough for that sort of work?*

*PA2 I think it depends on the service user you’re working with.*

*PA3 And also the strength of the peer. Because if you’ve got someone that’s a peer that’s quite reserved, they might just be, you know, take two or three sessions before anything gets going. So, that’s another factor isn’t it, you know? Because, I mean, the fact that you’ve got a set cut-off time, so, would it be ten sessions for one person with certain skills, or 13 for another person with another set of skills? I don’t know. I’m just throwing it open.*

*PA1 Or, for a client as well, different clients have different needs.(Staff – site 4)*

**4.2.2 Short term intervention (5 sources, 7 references)**

Service users and staff both discussed the short-term aspect of the PSW programme. Staff pointed out a positive aspect of a short-term programme in that it avoids the danger of people ‘could just be there forever… and never leave. They’re not really moving forward with their own life either’ (Staff – site 1)

Service users gave positive views of a ten week programme but suggested a longer programme could be better; and recommended tapered ending to the sessions (less frequent sessions towards the end of the programme):

*PA1 Ten weeks is fine, if you know, because of funding and everything as you said, it should be okay, at least you’re getting, look at that, you’re building yourself up, you’re being confident, you’re getting your confidence back, so that would help me really, you know? Just to get my confidence, so ten meets, not bad at all.*

*IV You think that would go quite a long way?*

*PA2 Yes, if they get the funding, then I can go for a bit more, but ten weeks is okay.*

*PA1 I think ten sessions is quite acceptable, yes.*

(Service user – site 2)

*PA I know on the whole that most people experience the crisis team as an almost daily contact, so I think I can imagine the first, say, five or six weeks being weekly. Those meetings being a standard reliable week apart might be really good and then, as you said, after that to get used to having longer gaps between meeting those supports, yes, instead of them being maybe once every two weeks and have the last one maybe for the month after that as how have you got on since a month ago. I think that, at least the beginning, I’ve known for me it’s better to have those closer together to keep up that regular... because even a lot can happen in a week mood-wise... so to have those closer together at the beginning is definitely good. (Service user – site 5)*

**4.2.3 Long term intervention (5 sources, 17 references)**

Service users, staff and carers referenced the long-term aspect of the PSW programme. Carers stated a preference for a longer-term or indefinite programme:

*IV About the length of time, what views have you got? How long should it be?*

*PA1 Forever.*

*PA2 Condensed it in sessions, yes. I think to put a limit on it… financially, we have to face the facts that you can’t be in the system forever, but if they are real, they should be in the system forever, and depression and things does sometimes last a lifetime, and people… it’s like cancer. You don’t push people out after one session and [unclear], so I think they should open up the boundaries and say, tailor to the individual, and they should be there forever for them, if needed.*

*PA2 You should be raging against this sort of cut off. You mentioned the word, probably unconsciously, cut off. That’s not a word that a service user needs to hear, is it?*

(Carer – site 3)

*PA And when you said it's eight to 10 weeks, the same thing, my son doesn't trust any professional, and if he knew it was going to eight to 10 weeks, he'll think well why bother, because at the end somebody else is going to be taken away.*

*It's like it could take eight to 10 weeks to build up trust. I'm thinking, can it not be open-ended in some way and maybe more a socialising thing, for social skills. But I think by putting that structure on it straight away eight to 10 weeks, I know him, he wouldn’t bother. I don't know, I like the idea but when you said it was eight to 10 weeks, I thought, God, here I go again, it's short changed. And that's because my experience, I'm talking from experience.* (Carer – site 1)

Service users similarly gave reasons for preferring a longer intervention:

*IV How useful do you think that would be to have up to ten meetings over two or three months with this worker after your crisis team has stepped out?*

*PA Well, if that begins and then after the ten times it finishes, then you’re going to be lost again. I think that feeling [unclear] you.*(Service user – site 1)

*PA1 Let’s discuss into exactly what we’re talking about of lack of continuity, and this is what we are saying. You’re dropped from one service, you’re dropped from something else, you’re dropped from something else, and, you know, you give your service users that hope that at least they’ve got someone who understands them and wants to work with them, and then the same person who’s been through the system is there to support them, after ten weeks they drop them, and then…*

*[…]*

*PA2 But they should keep them on for a lot longer, I mean it should be a permanent job because…*

*PA1 Six months, at least.*

*PA3 Six weeks is too short, yes.*

*[…]*

*PA1 Just looking at an example like that, it’s taken almost a year, and what difference can you make for a person in the ten weeks, because some people have issues that are longer, and even looking at situations that may make a person break down, it could be an anniversary of a death of a parent or a loved one or a pet and it could take not the ten weeks only. […] That short period of time could actually cause a lot of pressure and stress on the person because if you’re, you know, going and seeing someone and saying I’ve got just ten sessions with you and after that, you know, I’ve got to leave you, it will put so much pressure because, you know, you just think the situation is so big and ten sessions, it’s like you may even think, you know, well, let me try and cope with it on my own and see how it goes rather than be with someone who’s just going to be working with me just maybe two or three steps and then is going to drop me.*

(Service user – site 4)

**4.2.4 Discharge flexibility (4 sources, 8 references)**

Carers and staff mentioned discharge flexibility. Carers suggested that the programme should have ‘no formal end’ (Carer – site 3) and participants should be able to call the PSW beyond the programme:

*IV Do you think you ought to be able to call upon a peer over a period of time? So if there's months, or six months down the line, go and perhaps…*

*PA Definitely, and possibly with the same person even.*

(Carer – site 1)

Staff similarly suggested that discharge at 8 or 10 weeks might not be suitable for all participants.

**4.3 Regularity of sessions**

**4.3.1 Sessions per week (4 sources, 6 references)**

Staff and service users stated preferences regarding the number of PSW sessions to be held per week. Service users recommended flexibility in the number of sessions per week to suit the participant:

*PA And people are individuals. If we say they’re getting ten sessions, I think those ten sessions need to be tailor made around the individual. Some people may want ten sessions on a weekly basis, some people may want ten intensive sessions like, say, twice a week on a maybe Monday and a Thursday, or whatever day they decide, and that short sharp intervention is what some people need. Other people may decide I want to meet up with you initially for three close sessions and then have time to sort of work on things, on confidence, and work on things and may draw it out…*

(Service user – site 4)

*IV And, with the ten meetings that you’d had, would you want them to have those quite close together, or a steady, one each week, or how would you like the meetings?*

*PA1 I would prefer once a week.*

*PA2 I would prefer a couple of times a week.*

*IV Once a week, twice a week?*

*PA2 Yes, twice a week, yes, I’d prefer that, yes.*

(Service user – site 4)

*PA I just thought that’s a… just seems like a nice average, sort of regular period of, you know. I think once a week is not enough, perhaps a… It depends how bad you are at the time. I mean, if you’ve managed to get off your feet and you’re keeping yourself busy, you’ve got a routine and you’ve got commitments and things, I think once a week, but it depends really how… what your schedule is. […] If you’re someone like poor Donna, who’s really bad, who can’t be in the house by herself, sometimes people need, you know, a visit nearly every day because they can’t handle being on their own. It just depends really how you are.* (Service user –site 3)

Staff advised that one to three sessions per week could be useful for different participants; although one session per week could be too much for others (Staff – site 5)

4**.3.2 Flexibility recommended (3 sources, 5 references)**

**4.4 Location of sessions**

**4.4.1 Location need based (8 sources, 11 references)**

Carers, staff and service users gave pros and cons of a home location. Carers gave mixed examples:

*PA1 I’m assuming it would be in the person’s home. I hadn’t thought of that one, if they got to come out.*

*IV Well, that’s certainly one we want your views on is where do you think it should actually take place.*

*PA1 Because there’s a lot of stigmas with mental health problems, and I know that Tim’s found it really beneficial having all these home visits, but then I find also, you have all these strangers coming in your house, and you’re giving your most personal information away, and that’s kind of strange.*

*PA2 I think it’s down to the individual, where they would want to have the talk to take place, what they would be comfortable in.*

*PA3 Annette would be home, wouldn’t she, because she’s got the dogs with her and it’s a comfort zone. That’s a lot of people’s comfort zone is their home, isn’t it? On the other hand, that’s where the place of most upset is for some people.* (Carer – site 3)

Staff similarly argued for different people having different preferences for location:

*PA ‘Everyone’s so different […] [Overtalking] having people round to theirs, some people absolutely can’t stand having people round at their place* (Staff –site 1)

Some service users expressed preferences for a home location:

*PA I think it's important for the person that is actually a guest at the client’s home, is that the client is able to be in control.*

[*<CI_SU_02.03.2012 (2)>*](file:///C:\Users\rejukfu\AppData\Local\Microsoft\Windows\Temporary%20Internet%20Files\Content.Outlook\55ZWPWJ7\7ce3771e-1eca-4cf1-b8d0-242c4d434eac)

*PA1 So for me to have someone… the option of having someone come to my home would be good.*

*IV So that would be a possibility.*

*PA1 Yes. So I suppose it’s depending on the individual person, isn’t it?*

[*<NELFT SU FG Parts 1 and 2>*](file:///C:\Users\rejukfu\AppData\Local\Microsoft\Windows\Temporary%20Internet%20Files\Content.Outlook\55ZWPWJ7\d9c9bcbb-2cc6-4a0b-a5d0-237f5f92f43b)

*PA2 Well, I’d be happy round my house, because I’ve got a nice house, a nice little house, and my friend who I’m thinking about has been there before and if you know someone you’re at ease with all the time that’s no problem whatsoever.* (Service user – site 3)

However, other service users highlighted the importance of other options of a ‘neutral place’ such as ‘a café or a park’ (Service user – site 4) or alternating between the ‘privacy’ of home and somewhere outside that’s ‘not too crowded’ (Service user – site 2).

**4.4.2 Location safety based (3 sources, 5 references)**

Staff and service users highlighted some of the safety requirements of the location of meetings between participants and PSWs. Staff recommended that PSWs have access to risk assessments to determine ‘whether it’s safe to visit’ the participant (Staff – site 1) ; and advised that the meetings should take place in a ‘hospital’ or ‘neutral place’ on account of risk (Staff – site 2).

A service user pointed out a need to establish a relationship and safety before the PSW visits the participant’s home:

*PA So I think maybe that first meeting could be in an environment that’s away from the house so that you don’t have any detractions, and when you do the phoning… and then people can get a feel of whether… the peer support worker can have a feel of whether they are comfortable to visit that person’s house, because I think you also need to take in consideration the person who’s doing the support, and while someone may say, oh, come to my house, you also need to feel comfortable in going to the place, so there should also be that choice for the worker because you’re going to be working alone.*

*IV Well, I understand your point, yes, for safety reasons.*

*PA Yes, safety. (Service user – site 4)*

**4.4.3 Encourage community locations (6 sources, 14 references)**

Carers, service users and staff made recommendations for meeting in community locations.

Carers suggested that going out can help participants to overcome agoraphobia. Suggestions were to go out on a bus or to a library (Carer – site 3). Carers recommended meeting in an ‘anonymous place’ rather than ‘mental health environment’ (Carer – site 1)

Staff suggested parks, coffee shops, and ‘shopping’ as additional option to meeting at home; and recommended that meetings are not held in ‘depressing’ hospitals (Staff – site 5).

Service users made recommendation to ‘go out somewhere’; ‘getting you out of the house’ and for improving ‘independence’ (Service user – site 4). Other suggestions were for a day to trip to London or coach trip outside of London (Service user – site 2),

coffee, shopping, or game of bowling to ‘have a laugh’ and improve ‘socialising’ (Service user – site 3).

**4.5 Content of intervention**

**4.5.1 Signposting (7 sources, 18 references)**

Carers suggested that a ‘knowledgeable’ PSW could help with finding advice about topics such as employment (Carer – site 3).

Service users recommended that the PSW could signpost to ‘information on the borough’ and ‘local resources’ (Service user – site 4). Others commented that it’s ‘useful to know other places that you can use the support’(Service user -site 2). In particular, one service user suggested that the PSW signpost to means of support for when the PSW programme has ended:

*PA I feel that what’s in place at the moment, people who I’ve come into contact with, it’s been really good about alerting to other services in the area or under the same Trust, but certainly to leave people with an idea of where to go next or what could happen now is really supportive because people don’t always... they’re not always told or somebody else thinks somebody else is going to tell somebody. So it can be really helpful to know where those next ports of call might be.*

*IV Like the voluntary sector or so on for support.*

*PA Yes. (Service user –site 5)*

Staff suggested that PSWs could help people to ‘integrate back into society’ and avoid ‘social exclusion’ (Staff – site 4)

**4.5.2 Social engagement (10 sources, 27 references)**

Carers highlighted the advantage of PSWs helping participants with a hobby (Carer – site 3), and that social engagement could be enhanced with a longer-term intervention:

*PA It's like it could take eight to 10 weeks to build up trust. I'm thinking, can it not be open-ended in some way and maybe more a socialising thing, for social skills.* (Carer – site 1)

Suggestions made by service users for PSWs to enhance the participant’s social engagement were helping the participant to do voluntary work or ‘just get out in the community’ (Service user – site 2), ‘have a chat’ with someone who isn’t a health care professional (Service user – site 3) and helping with ‘day to day things’ such as cooking and personal hygiene (Service user – site 5).

Staff highlighted the benefits of working towards social inclusion:

*PA I think on one of the things that [another participant] has picked up on, I agree with the thrust towards social inclusion, but actually it’s one thing to have removed all the mental health focus services, without actually providing people with the support to be able to access the things that are out there, or, to make people who are out there in those groups open to having people with mental health problems joining them. And I guess that’s one of the things that is really missing in mental health services, you know, we no longer have that, sort of, time to be able to offer to people in support time. Recovery workers do a little bit more, but not a huge amount. (Staff – site 4)*

Staff commented that there could be benefits in a programme incorporating social engagement and a more structured intervention (Staff- site 5).

**4.5.3 Future plans and goals (4 sources, 9 references)**

A carer and service users made reference to how the PSW programme could help participants with future plans and goals. The carer highlighted that the PSW could ‘give them challenges to face their dreams’ (Carer – site 3). Service users suggested PSWs could help with ‘making plans’ for how the participant can follow their interests, and that this can be more beneficial than ‘counselling’ which the service user had already experienced (Service user –site 2)

**4.5.4 Confidence building (6 sources, 10 references)**

Carers and service users suggested that PSWs could help to build the participants’ confidence. Carers gave examples of increasing confidence in going out, and in using the workbook to see positive changes over time (Carer – site 3).

Service users also highlighted the importance on confidence-building within the programme:

*PA And confidence building is a very important [unclear], especially in my case, so that should be a key thing. And emotional support, and stuff like that, just to try and build up so you’re confident and emotionally feel strong enough to do the things that you need to be doing, or want to be doing.*

(Service user – site 4)

*PA But yes, I mean, somebody who would come in after the home treatment team have seen you, just to help you focus on the future, and look forward. Yes, don’t focus on the negative, rather the positive. Yes, and just...let them, you know, let you know what’s out there for you, as a person, just things maybe you can do or not do whatever, you know? Setting goals really and very small steps.*

(Service user – site 2)

*PA Yes, I suppose there’s something that’s quite, to some people, a little bit patronising or condescending about focusing on fairly little things, but I think if you can see those little bits of progress it can be really encouraging.(Service user – site 5)*

**4.5.5 Crisis planning and relapse prevention (7 sources, 12 references)**

Service users, staff members and a carer recommended that crisis planning and relapse prevention constitute a part of the PSW programme. The carer spoke of the importance of stopping a situation from ‘getting to a crisis’ (Carer – site 3).

Staff suggested that PSWs work with the participants on the mini-WRAP crisis prevention programme (Staff – site 5)

A service user expressed approval for the use of a structured crisis prevention plan (Service user – site 3) One other service user gave an example of how the PSW could help the participant to identify warning signs in order to avoid a future crisis:

*PA Certainly, yes. I think rather than them being a generic list of warning signs that perhaps have been presented to the service user, if a peer support worker was to identify together with whoever was using the service what their warning signs were and to make sure they’re aware of their own because there’s a tendency to be, like, these are people’s general warning signs and in fact they can be really different for different people and to personalise it in that way because that’s, I think, what really makes a difference – when you feel like it’s relevant to you. Sometimes it can be so prescribed and then it feels irrelevant and then it’s very difficult to connect with the service and somebody who’s telling you what the warning signs should be and, in fact, that they’re completely different for you. (Service user – site 5)*

**4.5.6 Practical support (4 sources, 8 references)**

A staff member and service users included practical support in their suggestions for what should be included in the PSW programme. Staff suggested that PSWs give ‘practical advice on how to do things’ (Staff – site 1).

Service users made suggestions that PSWs help participants to fill in forms and help to coordinate care from different services (Service user – site 4) helping with problems (Service user – site 2); and helping with ‘basic things’ such as cooking and basic hygiene (Service user – site 5).

**4.5.7 Provide company – befriending (7 sources, 14 references)**

Carers, staff and service users highlighted similarities between peer support work and befriending, or suggested that befriending could be an element of the PSW programme. A carer commented that PSWs are ‘coming really as a friend, in a way’ (Carer – site 3).

Staff highlighted benefits of a previous MIND befriending scheme (staff – site 4) and pointed out the importance of ‘contact with other human beings’ (staff – site 3).

Service users gave example of how befriending could be beneficial:

*PA If somebody’s there, listening to what you've got to say, it helps. Like afterwards you feel a lot better after you got it off your chest. And if somebody’s there, you can just... you can rely on them, otherwise you’re sitting there by yourself and you’re... you have to think for yourself, and sometimes that can be just too hard.*

(Service user – site 1)

*PA Yes, and I think a big part of it is about the company and it’s about the support that they’re offering. (Service user – site 3)*

**4.6 Key components of delivery style**

**4.6.1 Tailor to individual (9 sources, 21 references)**

Carers suggested the programme should be tailored according to participant ‘intellect’ and technology use:

*PA I know when our daughter took part in the self-help program, she was in a group, and with mental illness there’s such a wide diversity of intellects from tower intellects to people who can’t read and write, you can’t apply the same tool to everybody. It has to be tailored to their intellect. I think we picked up on that. My daughter would be put off by something that is too simple. She needs the challenge of the book, if you like, and I think that’s what we kind of picked up here. It needs to be tailored, I think, to the individual to be meaningful or, shall we say, different versions of the same book to match… it’s a horrible thing to say, but to match the intellect of the patient or the service user. […] Well, again I think it would have to be tailored to the individual. Some people in our society… your service users or not… don’t use PCs through choice. Others don’t because they haven’t got access to one at home. They can’t afford it; they simply can’t afford it maybe. Others are technophobe… not technophobes, but they love technology and they would grasp the opportunity to do it that way.* (Carer – site 3)

Staff pointed out that ‘everyone absorbs things in different ways’ (Staff – site 1)

and a need for the programme to be ‘based on that client’ (staff – site 4) as well as highlighting a more general need for individualised working:

*PA I think it would be useful to have a range of interventions [general agreement] - for the bipolar patients, for the schizophrenic patients - and they should be well connected expert patients, so maybe they’ve experimented with coming off their medication in the past and then restarted it.*

(Staff – site 3)

*PA1 It depends on the person actually, because some people do like having a lot of input and some people don't want as much input, so I think it's just knowing the patient and knowing what they like.*

*PA2 Mental health is never that straightforward. Whatever the patients feel so if they're led by them, really, what they find is useful, led by the service user.*(Staff – site 5)

Service users recommended that the PSW programme is tailored to individuals according to how frequently they would like the sessions (service user – site 4) by conducting an ‘assessment of needs’ or matching PSWs to individuals (Service user – site 3) and for PSWs to be aware of individuals’ ‘warning signs’ (Service user – site 5).

**4.6.2 Range of options (3 sources, 3 references)**

A range of options were recommended for delivery and content by service users and staff.

*I think it would be useful to have a range of interventions [general agreement] (Staff – site 3)*

**4.6.3 PSW motivation and encouragement (5 sources, 11 references)**

Service users, staff and carers felt the PSW would be able to build confidence and provide encouragement, motivation and support to an individual.

*And confidence building is a very important [unclear], especially in my case, so that should be a key thing. And emotional support, and stuff like that, just to try and build up so you’re confident and emotionally feel strong enough to do the things that you need to be doing, or want to be doing.* (Service user – site 4)

*So having something that you’ve been encouraged to think about or to practice during the week I think is really helpful.* [*(Service*](file:///F:\Development%20paper\Appendices%20for%20BMC%20research%20notes\8ed4246d-869f-4969-bbd0-237f622ab90d) *user – site 5)*

**4.6.4 Develop trust (4 sources, 8 references)**

Developing trust was seen as crucial part of the peer work by service users, carers and CRT staff.

*I just want to understand that really one of the big issues wherever there's going to be peer support, as other supporters, is to gain trust of the person who they are going to work with.*

(Carer – site 1)*-*

**4.7 Who else involved**

**4.7.1 Carer-family-friends involvement (12 sources, 35 references)**

Some carers, staff and service users felt that they would like to occasionally be involved in the PSW sessions

*I think it would be nice for the carer now and again to sit in on a session, like we sometimes sit in on a session with our daughter with the home nurses…*(Carer – site 3)

*The second issue with this is, often the carers who are often forgotten in the mix of this because they become the gatekeepers to a patient being readmitted. If things are going wrong, and it's often the carer that has to... the crisis team go through the carer, the carer can’t cope, the carer needs a break, the patient goes back into hospital setting or the crisis house and it can become like a conveyer belt. So I think the carer has got to be included in the mix; he or she has got to be included in that support because if the carer’s neglected, then the patient is neglected also. That's very, very important.* (Service user – site 1)

However, this needed to be approached in a thoughtful, service-user centric way as some people may not wish to have involvement from others.

*Because sometimes, you know, we see it every day when you’ve got someone who says, I don’t want my mum to know, don’t talk to my mum, the chances of them relapsing are very high, because even no one is encouraging them to take meds, or, whatever it is. So, if there was a way of actually trying to get the network around that person involved in their care, I think it can only be positive for them if they are willing to do it.* (Staff – site 4)

**4.7.2 NHS staff involvement (7 sources, 10 references)**

Involving CRT staff in some capacity was also viewed as potentially helpful in most cases.

*I found that the more that mental health professionals and the services share their information the better because having to... including GPs...* [*(Service*](file:///F:\Development%20paper\Appendices%20for%20BMC%20research%20notes\8ed4246d-869f-4969-bbd0-237f622ab90d) *user – site 5)*

*But I also agree there needs to be a professional involved somewhere along the line I think.* (Carer – site 1)

Having CRT staff involved at the beginning to set the scene and at the end for reflection was suggested by one service user.

*Maybe you could do a kind of like, you see them, mental health workers and psychiatrists at the beginning, when you first see these peer workers, and then on your last session with the peer worker, you have another meeting, so your beginning and your end, and you see the in between, what’s helped or not helped, and how you’ve moved forward and not moved forward, or something.* (Service user – site 2)

One staff member raised that it was important that roles and responsibilities should be clear when staff and PSWs are working together.

*…because then the boundaries are getting blurred, don't they, as who's responsible and who's…* (Staff – site 5)

**5 PSW qualities and skills**

**5.1 PSW personal qualities**

**5.1.1 Positive PSW personal qualities (7 sources, 26 references)**

Many service users focus groups and one carer highlighted the importance of positive PSW qualities such as empathy, compassion, sensitivity, non-judgemental, emotionally intelligent, friendly, have good listening skills, were able to manage stress and were respectful. The PSW should be able to put the person they were supporting for at the forefront.

*It’s not about you, it’s about… it might help you as a peer worker, but it’s about the person you’re going to support, and ability to be able to put them first, because they’re the one that needs help, so all those things need to come into training, even though they might be common sense, but we tend to forget sometimes that emotions are in more… keep your emotions out, and that kind of thing.* [*(Service*](file:///F:\Development%20paper\Appendices%20for%20BMC%20research%20notes\d9c9bcbb-2cc6-4a0b-a5d0-237f5f92f43b) *user – site 4)*

The personnel recruited to the PSW role was viewed as vital for the success of the program.

*but I think great care will need to be taken about the people who are chosen, and the training, I think that’ll be really key to the success of the project.* [*(Service*](file:///F:\Development%20paper\Appendices%20for%20BMC%20research%20notes\d9c9bcbb-2cc6-4a0b-a5d0-237f5f92f43b) *user – site 4)*

**5.1.2 PSW lived experience (11 sources, 39 references)**

Service users, carers and staff focus groups all emphasised the importance of the PSW having a lived experience of poor mental health or using crisis services.

*I think to be a peer support worker, you've 1eally got to have some experience of what the other person has gone through.*  [(Carer – site 3)](file:///F:\Development%20paper\Appendices%20for%20BMC%20research%20notes\1f6fea73-fba7-449e-a8d0-237f5e7a2f2d)

*UM I think it is helpful that someone that helps you has actually gone through some similar things than yourself and they can understand more. And a lot of people at the crisis centre haven’t had mental health problems; they’ve just come out of school and they think they know, but they don't understand what you’re feeling, really. They know through text books what you’re maybe feeling but not the actual [overtalking].* [*(Service*](file:///F:\Development%20paper\Appendices%20for%20BMC%20research%20notes\d9c9bcbb-2cc6-4a0b-a5d0-237f5f92f43b) *user – site 1)*

**5.1.3 PSW wellness (6 sources, 14 references)**

When working, both staff and service user focus groups highlighted that the PSW had to look after their own mental health.

*One of the advantages is they’ve often gone through some of the things themselves and have some sort of awareness of the difficulties that perhaps we might be less aware of. But then I think the risk is that I suppose it depends how well the person is who’s offering the peer support and whether there's some sort of monitoring of that and what happens if they become unwell themselves and the stress is put on them by the process of it.* [*(Staff*](file:///F:\Development%20paper\Appendices%20for%20BMC%20research%20notes\a95dfe6c-948a-4f37-8cd0-237f610d6143) *– site 2)*

*And, you know, your own confidence, not to bring your own issues into the job, and being able to take on other people’s issues but not let it affect your mental health.* [*(Service*](file:///F:\Development%20paper\Appendices%20for%20BMC%20research%20notes\d9c9bcbb-2cc6-4a0b-a5d0-237f5f92f43b) *user – site 4)*

**5.1.4 PSW role balance (4 sources, 7 references)**

Balancing the role of being a PSW by not acting as a care-coordinator nor a friend was seen as difficult, but vital.

*Obviously the boundaries have to be set about personal relationships and things like that. It’s easy to get… when you feel… I don’t think I’d ever make a caretaker, because I just want to help everybody. Blimey, how can I help this person? So there has to be real strict boundaries, doesn’t there? You’re a professional, but you’re also an amateur in that college. That’s going to be a challenge, that one, I think, to be a peer worker, a real challenge.* (Carer – site 3) *-*

This required focused support and supervision.

*think there has to be a lot around how the peer themselves, the peer support team they are to be protected and supported. And there has to be really a lot around really how we would support that.* (Staff – site 4)

**5.1.5 Impartiality (3 sources, 8 references)**

The impartiality of the PSW was seen as important so they did not rely on simply giving advice and rather provided support to help the person make their own decisions.

*It's like getting other people with mental health issues in the past supporting those that have a mental issue, they're not qualified, they know how they feel, because we can all go through and experience and relate to someone else how they feel, but we're not qualified to give advice. And that advice may not be the right advice, and that would be a danger in my opinion, because if someone's advising someone else to do what they did and it's not the right thing, then that person's going to probably be worse off.* (Carer – site 1)

**5.2 PSW recruitment**

**5.2.1 Local demographic considerations (2 sources, 4 references)**

Service users and staff highlighted that taking into account the local demographics was important. This might include culture or specific local community needs.

*So I think in what you are doing with this project of yours, you may have to rejig or revisit what you are doing so that you are actually reflecting the needs of the people in the community that you want to engage with. And you also need to look at the demographics of the communities…* [*(Service*](file:///F:\Development%20paper\Appendices%20for%20BMC%20research%20notes\d9c9bcbb-2cc6-4a0b-a5d0-237f5f92f43b) *user – site 4)*

*that’s why I think it’s important when they’re recruiting the peer support workers that you’re taking people from every single ethnic culture.* [*(Service*](file:///F:\Development%20paper\Appendices%20for%20BMC%20research%20notes\d9c9bcbb-2cc6-4a0b-a5d0-237f5f92f43b) *user – site 4)*

**5.2.2 Role suitability (7 sources, 15 references)**

The PSWs to be recruited were seen as needing to suit the role it terms of above-mentioned PSW qualities. The selection process was seen as vital to ensuring that the PSW was a good fit for providing support to others in the programme.

*but I think great care will need to be taken about the people who are chosen, and the training, I think that’ll be really key to the success of the project (Service user – site 4)*

*The only thing I was thinking just having, somebody having had a mental health problem in the past isn't enough to make them, just because that's what's happened that isn't going to make them good at this role. There're other characteristics they'd probably need to have. They need to be at least a little bit caring, hopefully, and have some ability to empathise with people …* [*(Staff*](file:///E:\peer%20support%20development%20paper\Revised%20JULY%202017\972fdd7c-49b0-4cff-bad0-237f6158260c) *– site 5)*

**5.3 PSW training**

**5.3.1 Quality training (8 sources, 20 references)**

The training was seen as needing the be of high quality. The topics for training included safety and risk management (8 sources, 17 references), equality awareness (1 source, 5 references), training about the local community and its resources (2 sources, 2 references), training in relation to setting and maintaining boundaries of the role (4 sources, 6 references) and training concerning awareness of and handling relationship dynamics (3 sources, 4 references).

*you certainly need some formal training as well about boundaries and things like that because people should not obviously form personal relationships and things like that. So you do need this professional training, but the service user experience is invaluable, in my opinion* (Carer – site 3)

*Firstly and foremost, they need things like equality and diversity, they need training in safeguarding, and they’ll need training in things like customer care or, you know, how you treat people.* [*(Service*](file:///E:\peer%20support%20development%20paper\Revised%20JULY%202017\d9c9bcbb-2cc6-4a0b-a5d0-237f5f92f43b) *user – site 4)*

*There's a potential risk associated, such as what the training would be [inaudible] and how does the client choose, who's responsible for choosing them, and who’s responsible for, they've chosen them and then there's an incident either with a client or their support worker and how does the person manage the relationships, if there's an actual violent incident or something of that sort, how do you manage the relationship that might form between them if they're not experienced in doing that.* [*(Staff*](file:///F:\Development%20paper\Appendices%20for%20BMC%20research%20notes\a95dfe6c-948a-4f37-8cd0-237f610d6143) *– site 2)*

**6 PSW supervision and support**

The PSW supervision and support was viewed as crucial by service users, carers and staff. There was a need for this to be of high quality (6 sources, 13 references) and conducted on a regular basis. This would cover areas such as maintaining clear boundaries in the PSW role (3 sources, 4 references), debriefing and providing support channels for the PSWs (6 sources, 14 references).

*what kind of support are you going to have in place for the peers? Are they going to be getting regular supervision if they're having to support somebody who's also going through difficulties? Because they can relapse; they can get relapse taking on other peoples' stress and…* (Staff – site 5)

*And also training on lone working, because I think people will be working alone, and how they can safeguard themselves. I think it’s also important to have supervision, or access to some kind of talking therapy in case your workload has… people who have such high needs, or some things are said and you need to have somewhere to offload. There needs to be that mechanism, even though it’s ten weeks, but you don’t want to wait until the end of that when you doing the report back, or something, there needs to be access as and when.* (Service user – site 4)

This regular supervision was seen as needing input from a health professional (3 sources, 7 references).

*The other thing is the peer worker should have a direct line to, shall we say… I don’t want to say professional because the peer worker will be professional, of course, but to, shall we say… what do we call people in workers… I don’t know what… crisis team workers… you should have a direct line where you can, on your mobile, dial that, get an answer, and maybe get some professional help that you’re not quite sure of. Because there are going to be instances from peer workers who maybe need the professional advice of a full-time social worker and/or nurse, and it would be helpful to you to impart that to your… the person you’re looking after… so a direct line, a help line almost, to peer workers.* (Carer – site 3)

**7 Ending the intervention/ finishing the peer support sessions**

Finishing the peer support sessions was seen as a time to be mindful of as some people might feel abandoned. Risks relating to time limited supports were referenced by staff, service users and carers alike (5 sources, 12 references).

*That short period of time could actually cause a lot of pressure and stress on the person because if you’re, you know, going and seeing someone and saying I’ve got just ten sessions with you and after that, you know, I’ve got to leave you, it will put so much pressure because, you know, you just think the situation is so big and ten sessions, it’s like you may even think, you know, well, let me try and cope with it on my own and see how it goes rather than be with someone who’s just going to be working with me just maybe two or three steps and then is going to drop me.* (Service user – site 4)

*It does in a way, though, because if you put an endpoint on it and they don’t feel ready for the end, then that can set them back. So if you say most people need eight sessions, and you’re still going through at 12, that would kind of make you feel like you failed.*  [(Carer – site 3)](file:///F:\Development%20paper\Appendices%20for%20BMC%20research%20notes\087a611f-b0a3-4df6-83d0-237f5dd9f716)

Service users previous experiencing of using crisis services highlighted the need to end in a planned and supported way.

*They’re already told you about, they’re going to be ending it with you, but...they should have done it a bit more subtly, if you understand what I mean. When they ended it, I was kind of, how can I say this, disorientated, if that’s the word that you use.* [*<Internals\\SLAM SU group 30-10-12>*](file:///F:\Development%20paper\Appendices%20for%20BMC%20research%20notes\bb3f4820-7809-47fb-abd0-237f60c5a9c8) *-*

To manage ending effectively, it was suggested that the individual being support should be informed/ have an awareness of the program length from the outset, tapering the sessions, linking the individual with additional services, proper planning and ending on a positive note.

*And if it is for longer, just gradually make the gaps… extend the weeks in between. Start where they said once a fortnight, and then gradually once every three weeks, and then just gradually taper off, but make sure that somebody is still going there as needed.* (Carer – site 3)

*SI think you could make it into a positive thing, you know, do something special together, and look back at what you’ve learned together, in the last ten weeks or something, focus on the positives, you know, and say, okay, if you feel you still need extra support, well, there’s this organisation, or that organisation, and they can maybe push you in that direction, or put some plans in place, that you can, you know, that you have got people to go to, rather than just think, that person’s gone, what do I do now?* (Serviec user – site 2)

*Well, I think the peer might be able to really help the person identify where structurally they’ll be able to get other ongoing support.* (Service user – site 4)

**Sites**

Sites 1, 2 and 5: inner London NHS Trusts

Site 4: Outer London NHS Trust

Site 3: Rural NHS Trust, Southern England
