# Supplementary material for: Development of a peer-supported, self-management intervention for people following mental health crisis
Source: BMC Res Notes. 2017 Nov 9;10:588. doi: 10.1186/s13104-017-2900-6 (PMC5680762; doi:10.1186/s13104-017-2900-6)
Supplement: Supplementary file 4 — Additional file 4: DS6. Feasibility testing (stage 4)—main themes from participant interviews. [file 13104_2017_2900_MOESM4_ESM.docx]

**DS6: Stage 4 Feasibility Testing: main themes from participant interviews**

Results are presented in eight primary themes of; 1) Engagement barriers; 2) Positive qualities of peers values; 3) Programme content; 4) Workbook; 5) Programme impact; 5) Programme Endings 6) Programme set-up; 7) What was helpful about the programme; 8) What was unhelpful about the programme; 9) Ways to improve programme. Figure 1 is a representation of these primary themes and sub theme


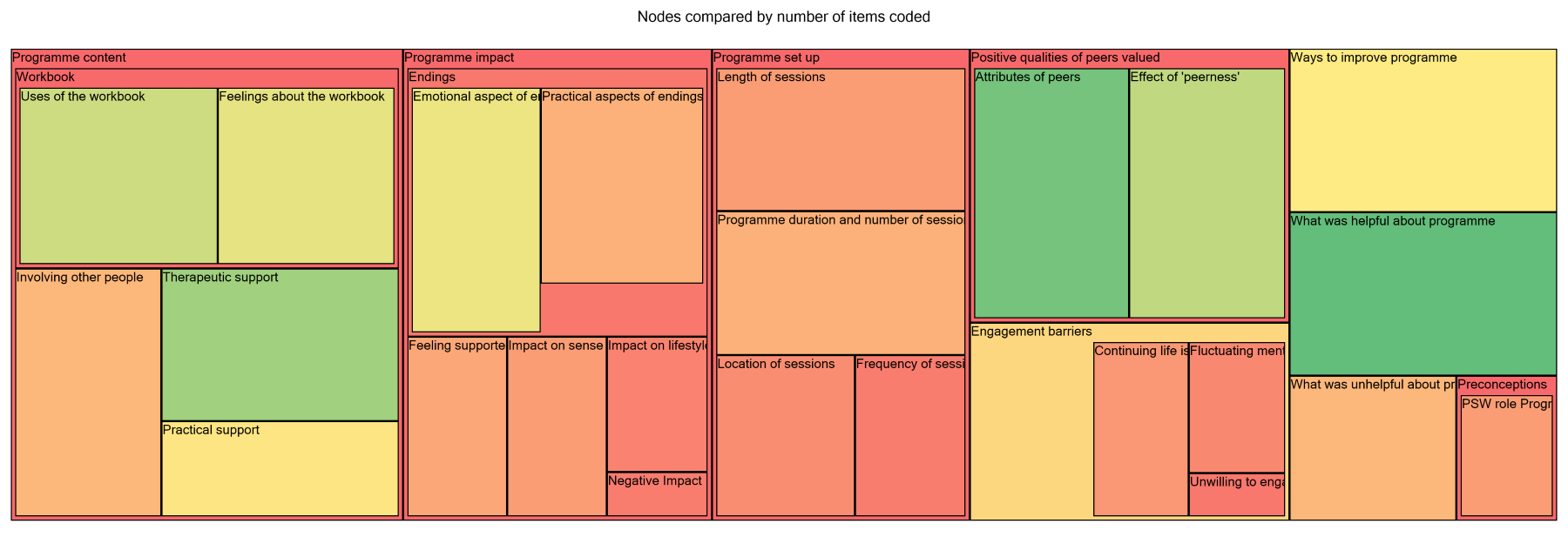


Figure 1. Primary themes, themes and subthemes by number of items discussed

**1. Engagement Barriers**

Five service users (55%) explained why they had found it difficult to engage fully with the programme. The themes which came through engagement barriers were: fluctuating mental state, continuing life issues and not gaining from the programme.

**1.2 Fluctuating mental state**

Four participants (44%) referenced their fluctuating mental health as a barrier to them engaging fully with the programme. Often these participants missed appointments with their peer due to feeling unwell.

*PA I said to her I wasn’t well, and I didn’t feel like going out, or I’d stay in bed, yes, and that, and I was sorry that I didn’t meet her, and that. She come out of her way to meet me, yes, she literally come out of her way to meet me.* (PP11)

One participant spoke specifically about finding it difficult to fully engage with more practical aspects of the programme, such as activities in the workbook, due to their fluctuating mental state.

*PA At the beginning, I sort of started off. I found relevant quotes on each. But then, because of my stress and panic attacks, we sort of had to stop. So, at the end, [PSW name], she sent me… she just did all the notes for me and she sent them to me.* *(PP09)*

**1.3. Continuing life issues**

Four participants (44%) spoke about their on-going life issues (e.g poor physical ill-health, issues amongst family/friends, drug/alcohol abuse) interrupting their engagement with the programme.

*IV So, was it more that you had other things going on in your life that you couldn’t meet with [PSW name], or that you didn’t want to?*

*PA No, it was because I was… nine times out of ten I was ill because I got [unclear] as well, and my heart was playing up for two weeks before I got into hospital and I had an operation on my heart. And then when… I was still in hospital when we were going to meet and then I had to take things easy.* *(PP11)*

*IV Do you think that doing this programme has helped... has, kind of, helped you...?*

*IE A lot. It was just my mistake. I ended up drinking again. That's all. But apart from that she was, like, curing me really.* *(PP05)*

**1.4. Not gaining from the meetings**

Only one service user (11%) expressed the reason that he did not engage with the programme after the first two meetings with his peers was that he did not find the initial sessions useful. This participant said that he felt that having a peer worker did not meet his needs, so he withdrew from meeting with his peer for any further sessions. This participant felt that having been allocated a peer worker was taking away from other, more immediately useful, aspects of his care such as receiving his medication.

*IE It just weren’t… it weren’t unhelpful, it just weren’t helpful, if you know what I mean. It weren’t like it was bad; there was just no point to it (PP07)*

*IV You said that you felt that she wasn’t really qualified.*

*IE No, it was just basically like… I don’t know, because I was on medication still and I said, I won’t be able to get any, and it was like no point talking to her because she couldn’t help me with that, and because I was talking to her the Crisis Team… well, actually, you can ask her, I phoned up and said they’re not helping me no more, so it was like talking to her actually put me… didn’t help me at all because I was using something else and the Crisis Team wouldn’t help me no more because I was using someone else, sort of thing, and they actually said they weren’t helping me.* *(PP07)*

**2. Positive qualities of peers valued**

**2.1 Attributes of peers**

Eight participants (89%) spoke very positively about their peers, attributing numerous positive personal qualities to them which impacted greatly on their enjoyment of the programme. The majority of participants spoke of feeling pleasantly surprised by how highly they thought of their peer.

*PA Yes. Well, from the time I had the breakdown and I met people even from the Crisis Team, but I felt like, [PSW name], she really committed herself and she was really, really lovely. Like a friend. I didn't feel… when I was with her, I didn't feel that I am actually participating in the programme. I felt like I am seeing a friend or someone from family.* *She was very loving, listening, caring, dedicated, reliable, and she was wonderful in listening. Much better than the people from Crisis. And also, she was very accepting, so she would accept you without judging, and she would look for things that would… that I would like, not force me to think that I wouldn't like them. But especially she was very listening and she, in a way, inspiring as well. (PP01)*

*PA I mean, she’s brilliant. Very professional, but very human and the fact that she had the same diagnosis as me was very, very helpful. And I was very sceptical about that to begin with, before I was paired with someone. Because I thought, oh God, it will be just about like the blind leading the blind and actually it wasn’t at all, it was the complete opposite. (PP03)*

*PA Some kind of warm... some kind of true warm... just like someone to be standing by you, you know? Very professional though. Well, I just felt like someone taking really care about, yes, about me, like something I would never even expect (PP05)*

*PA She was a very loving and caring person, and that come through the phone calls, yes, that she phoned me up and that, and everything. She was a kind and loving person and she was genuine. She was really genuine, she was, you know? She wanted to help, yes?* *(PP11)*

**2.2 Effect of ‘peerness’**

When discussing the positive qualities they valued of their peers, 8 participants (88%) mentioned how valuable they thought it was that their peer had previously been a service user and how much this benefited their experience of the support.

*PA Because she sometimes said something, from her own experience, and it was always… it wasn't like she was telling me things but it was always just little things but it was relevant to how I felt. She would make a suggestion where I can go or what can I read and watch out, and that would be always sort of spot on.(PP01)*

*PA I think so. Yes, because it was more being with a human, not with someone who learned things from the book. Because I am an intelligent person; I read lots of books and all that, but sometimes it's actually better to learn from a person who learnt from life, who went through things in life and experienced them organically, not just memorised them or something.* *(PP01)*

*PA And it’s nice to see someone who, you know, who has... who is recovering very well from symptoms that we both share, you know, and, you know, because one of my things is about worrying about getting back to work or having a relationship or something and we were able to talk about that.* *(PP03)*

*PA she was very, very empowering, you know, and I think the fact that sometimes we’d meet for coffee and tea and, you know, and one week I just said I’d like to hear your story, actually, and she was, like, completely open about it: Yes, okay, and so the next week she told me and I thought oh, my God, I think I’ve got stuff to... you know, and the fact that she’s risen above all of that is wonderful, you know, so it was... it felt almost, but not quite, kind of co-counselling but it wasn’t, because I didn’t feel that I was counselling her at any point. I just felt that I was meeting a really good friend. So, but I do think this is a very good idea.* *(PP03)*

**3. Programme Content**

**3.1 Therapeutic Support**

When describing different aspects of the programme, 8 (88%) participants consistently reiterated how much it meant to them to have emotional support from their peers. Receiving this kind of therapeutic support, really feeling like they had someone, was of central importance for all the participants.

*PA It’s really good. It does help me a lot, and I just, like, I just wish, like, she didn’t stop. I just wish she was still, wish she was still, like, meet me the same way, until I get sorted out, because through everything, she’s, when there’s time I want to really take my life, and just she really saved me a lot, because it’s mostly every night, she would give me a phone call, and all that. So, you know, she does help, like, she do so much. She maybe don’t do physical, but in words and phone call and all that, and the visits and all that, it does help me a lot, and I really appreciate everything she does…….like, she would stay on the phone for maybe half, like an hour and all that, and speak to me, and let me feel good in myself, and it does help me, because that’s like saving my life, so and that’s the greatest thing, you know?* *(PP04)*

*PA I appreciated the support she gave me, somebody at the end of the phone. I mean, she phoned me up, like I said, in the hospital and she said, get well soon, yes, to see you soon, and that. And that from a stranger was so nice to hear, that somebody cared about me without even knowing me, do you see where I’m coming from?* *(PP11)*

*PA Well, I think things like, you know, to do with human relationships and, you know, there’s somebody I like that I can’t talk to and now I can. Which has been much better, and yes, just looking at what works, you know, like I’ve been going to meditation on a Wednesday evening; I didn’t go this week. And I, you know, I tend to punish myself when I don’t do things I’ve planned, but, you know, I think she helped me flesh out and thresh out exactly what would be helpful to... you know, about keeping well and things to do on a daily basis. And, yes, I mean, this is a work in progress. It really is. It’s kind of... but, I feel like I’ve done... I could not have done this on my own, I know I couldn’t have done, I would have made it very, very focused on the negative.* *(PP03)*

**3.2 Practical Support**

4 (44%) participants also spoke about the practical support which their peers provided being of particular value e.g. attending meetings, helping complete forms, accessing services etc.

*PA She suggested that stress project. She went with me to GP. I don't think they referred me but she suggested this. She was looking for the best sort of therapies in the area for me. We couldn't find anything, but I know that she really tried. And also, she suggested that centre in Covent Garden where I can go and attend meditations and stuff like that for free. She went with me once to meditation, and it was this kind of meditation that I was interested in. It was transcendental meditation. And she just found out that there will be such an initial meeting and took me there. Also, she arranged all the help with my work. I don't think anything could happen if she didn't help me with this. And she has written excellent letter to my manager and that wasn't even part of her job. She didn't have to but she did, and this letter really helped me. And even the lawyer said that it was a brilliant letter. And while everyone sort of was telling me that they would help, but no one really did anything, while she actually did things. And I really, really appreciate this.(PP01)*

*PA Yes, I’m just saying if it wasn’t for her, then I wouldn’t really get that help that much, because the people that just come and see me today, there was a guy named Tom, of the same people, and when he leaved, he told me that someone should contact me in like, a week after him leave, and there was no one contact me. There was no one phone me, there was nothing, and she phoned them up and all that, and since she phoned them, they’re more interested and they more want to come and see me, and they more want to do things, so even like, just that alone, it’s really great. Really, really great, so yes. It’s okay, it’s really okay.* *(PP04)*

*PA Well, I think the fact that you’ve obviously trained your people very carefully in order to do this work. And I think the workbook is a very good idea,….So, and, you know, and then writing and, I mean, and the fact that [my PSW] was really helpful in helping me write it, because I’m really bad at filling in things because of myself, you know, for myself. But I found that easier as time went on.* *(PP03)*

**3.3 Involving other people**

When the participants were asked if they had been encouraged to involve other people in the programme, one participants spoke of sharing some of the work they had done in the work book with friends and a family member.

*PA And I showed my dad this and he was delighted. He was really sweet. And he said goals and dreams, that’s what you’ve got to focus on. That’s very much my dad. (PP03)*

*PA I showed a couple of my friends who... one of my friends worked in [place described] had a look at it, she went, wow, this is really good, we could use this for, you know, some of the people who are going to go out into the, you know, leave prison and everything. She thought it was excellent. So, yes (PP03)*

However, six participants (67%) expressed that they chose not to involve specific family members or friends with the programme due to personal tensions or circumstances within their social networks.

*PA [PSW name] suggested that if I had a meeting with her then we could do it with [PSW name] there and I just thought I wouldn’t do that to [PSW name]. I wouldn’t inflict my sister on her like that, because it’s... I’m not... I think [PSW name] very, very capable about doing that and I just chickened out. I just thought I can’t suggest that to my sister, because she would just fly off the handle (PP03)*

*IV So you didn’t tell your brother about the programme at all?*

*PA No, I didn’t mention it to him. I hardly talk to him really, only on the phone; I hardly see much of him.* *(PP08)*

*PA No, I didn't. I wouldn't have liked that. I would prefer totally on my own, totally. It's my recovery. They've got no business with me in that type of recovery. It's my business and my business alone and those to whom I want to reveal it to, anyway you know. So I wouldn't want anyone to interfere. Not even my wife.* *(PP10)*

*PA Because it’s taking a step, when people are in my life, it’s best to have somebody outside your life, yes, that you can talk to, yes, that won’t – how can I say it – will not take it in the wrong context, yes, and what I’m saying, like family would. That’s what I’m saying, yes, and she.. (PP11)*

Two participants (22%) spoke about how they had shown their workbook with MHS staff.

*IE I did mention it to my GP about this, I mentioned it to my therapist, I took this to show her (PP03)*

**3.4 Workbook**

**3.4.1. Feelings about the workbook**

*Positive Views*

When participants commented on their feelings towards the work book they all expressed that they found the peer support worker far more useful than the workbook. However, four participants (44%) were very positive about the workbook, its design and content.

*PA I liked it immediately, I liked all of it. I liked the layout, I like that bit, the picture on there, I like the colours. I think it’s very well balanced. I don’t think there’s too much. And there’s just enough, I think. There’s not too much and there’s not too little, but I think if people wanted to add pages they could.* *(PP03)*

One participant expressed that she would like the workbook to be available online so it would be easier for her to carry certain pages with her.

*PA If it would be available online then you could make smaller formats so that it's more handy. You can put it in your bag and actually have it with you. (PP01)*

*Negative Views of workbook*

Four participants (44%) expressed negative views of the work book, two participants (22%) spoke of preferring to discuss issues rather than to write them down as there is more opportunity for an open and individual dialogue, rather than sticking to the structure of the workbook.

*IE Maybe. You see, with me, I don't. You see, if I'm constricted by rules I don't function well anyway you know. If I know I've not any obligation to stick to certain specific ideas of people, anyway you know, I'm at my best, anyway, you know. I was free to talk to [PSW name] in a way that would, that I wasn't impeded or felt restricted*

*IV So you liked that the sessions were more informal rather than sticking to the book in going through it one at a time.*

*IE Absolutely, yes.* *(PP10)*

Two participants (22%) found the task of writing challenging which deterred them from using the workbook.

*IV No. I can write, it’s just basically I’ve got to write in block capitals and I get wound up, so I don’t write.* *(PP07)*

Two participants (22%) commented on changes they would like to see to the design of the workbook with one participant suggesting that there were more blank pages and space for notes or sketches. One participant (11%) did not like the quote on the front of the workbook as she felt it was not in keeping with her spiritual beliefs.

**3.4.2 Uses of the workbook**

Tool to use as platform for discussion with peer

When asked specifically about the ways in which they used the work book, all four participants who found the workbook useful expressed that working through and discussing the activities in the book with their peer was an integral part of them finding the workbook useful.

*PA Just like, she would draw the futures, you know, things that I want, and things that I want, and things that I plan for, like for instance, like I used to work. I used to do chef, I used to do engineer and all that, and she would put all those little things down, and my family, like, my kids, and I want to see my kids and things like that, so it does something, like, when I look back on it, it does make me think once and twice, what should I do with my life? . (PP04)*

*PA So that’s really good, you know, I mean it’s good for me to kind of read that out loud to someone. It’s really, yes... and then to bounce it off with someone. So I don’t know, if this is a template, someone like [my PSW] or someone like me would think it’s perfect. So, it’s worked very, very well.* *(PP03)*

*Tool to consult if stressed*

One participant found it really useful to consult her book if she was feeling stressed as it contained triggers and details of routines which can improve her well-being.

*PA even though I don’t look at it every single day I know, like, ….because I’ve been a bit wobbly the last few days because of this meeting at the borderline clinic, is I’ve... I’ve just thought, right, okay, I’m going to bed too late, I’m getting up too late and now I realise if that snowballs, normally it would snowball and then I’d be in crisis and then I’d be drawn [unclear] now, because we’ve written it in here, I know it’s stuck in my head so I wrote down last night: go to bed at a reasonable hour …. and get up at a reasonable hour. (PP03)*

*Tool to refer to in the future*

Three participant (33%) thought that the workbook was a useful tool as it allowed them to express their emotions clearly and felt that the permanence of written words meant that they could continue to use the booklet as a tool in the future, something they can look back at and refer to when needed.

*PA I wrote it down in words. I’m better off writing down, better than saying it in words, you know. I mean, if you write it down you got... you can study it more, you know, you can read it and digest it. But if you say it, it tends to go out the memory, doesn’t it, quick.* *… I think it’s great, because I went through that and it really helped me just to settle my mind and to read it and, you know, I haven’t done any of this at the moment but I will, because we did this.*

*It was a very, very, positive, you know, makes me think, oh, I want to go back and read this all over again, you know, because it’s really... it’s been very, very helpful.* *(PP08)*

**4. Programme Impact**

As presented in *Table 1*, the programme had a positive impact on the majority of participants’ lives, only one participant held a negative view of programme impact. The other 8 participants (89%) expressed a positive view of the programme and its impact on their lives and spoke about the impact of the support coming to an end.

***Table 1*. Participants’ views on Programme Impact**

| **Views on Programme Impact** | **Sources** | **References** |
| --- | --- | --- |
| **Positive**  Impact on sense of self | 4 | 10 |
| Feeling supported | 4 | 12 |
| Impact on lifestyle | 3 | 5 |
| **Negative** Impact  **Endings**  Emotional aspects  Practical aspects | 1  8  8 | 4  28  14 |

**4.1. Positive Impact**

Four participants (44%) expressed the view that the programme had impacted positively **on their sense of self**, increasing their confidence and helping them feel more able to cope:

*PA And I think these are very, very helpful, because it makes you realise what’s achievable. For me, it makes you realise, you know, that to me recovery means being in the driver’s seat. I think it’s fantastic.* (PP03)

*IE Yes, I think I have learned a lot about myself thanks to [PSW name] so I am very happy. (PP01)*

**4.2.** Three participants (33%) expressed the view that the programme impacted positively on their lifestyle.

*PA ... I wrote it down and then she would write other things…..it’s really helped me realise the very essential things that are going to keep me well. Like eating properly, cooking properly, you know, having a bath, and how important those little things are. Like I did the washing up before you came, rather than think oh, I’m just, you know, because it’s a little bit of a hair-raising day, but it was great, it was good, I survived it, so that was good, you know. (PP03)*

**4.3.** Feeling of being supported.

Four participants (44%) expressed views that the feeling of being supported has had a positive impact on their lives**.**

*PA There’s a few times I really give up, and just, because a few days ago, I was planning to get a piece of rope, and to come, to take my life, and because she told me, whenever I’m stressed, I need to, I must give her a call. I give her a call, and she was telling me everything is going to be all right….. and she’s there for me, so that kind of, just lifted me up a bit, let my mind change for a few, you know, for a few seconds, and let me think, it’s the greatest thing. I’ve got her number, and I can call her, whatever time I feel* (PP04)

*PA I appreciated the support she gave me, somebody at the end of the phone. I mean, she phoned me up, like I said, in the hospital and she said, get well soon, yes, to see you soon, and that. And that from a stranger was so nice to hear, that somebody cared about me without even knowing me, do you see where I’m coming from?* *(PP11)*

*PA Some kind of warm... some kind of true warm... just like someone to be standing by you, you know? Very professional though. Well, I just felt like someone taking really care about, yes, about me, like something I would never even expect.* (PP05)

*PA Help you along the way, yes. Yes, I can see that, you know. I think it has helped me. I tell you what though, I mean, I can see they helped me, because without people like [Worker name] and my doctor and [PSW name}, I’d be at a loss, because I wouldn’t be anywhere. I mean, they arrange things for me and I could never do it by myself, you see.* (PP08)

**5. Ending the Programme**

When discussing Programme Impact 8 participants spoke of the **emotional impact** the programme ending will have on them and how much they will miss the support provided by the peer worker and would prefer for it to continue.

*PA And it was nice to have someone to talk to even once a week, especially so warm and loving. Yes. And it quite upsets things since it's finished.* *(PP01)*

*PA It’s good, man. It’s really good. It does help me a lot, and I just, like, I just wish, like, she didn’t stop. I just wish she was still, wish she was still, like, meet me the same way, until I get sorted out, because through everything, she’s, when there’s time I want to really take my life, and just she really saved me a lot, because it’s mostly every night, she would give me a phone call, and all that. So, you know, she does help, like, she do so much. (PP04)*

*PA She was the best counsellor I could have... I could ever had... have. She was a very, very good one. I'll be missing her. Yes, I will.* *(PP05)*

Two participants (22%) expressed that **the short term nature** of the programme was difficult for them, losing that support once the programme ended left them feeling like they were back to the same point they were at before the programme started.

*PA Well, she also sort of helped me with confidence. When I was seeing her, at some point I actually felt very confident and happy, in a way. And it's all gone since I don't see her. It was like making a step forward and suddenly it's ten steps backwards….I mean, an hour a week is fine but if it would be for a longer period that would be better.* *(PP01)*

*PA Well, all I can say is I made a friend of him; I made a friend of him. We got to go out to London a few times on the underground and on the bus. But then again, after it’s all finished, you find yourself stuck in back to square one, you know. You say, right, well, I’ve had meetings now, I’ve talked to him, I’ve been to Buckingham Palace, Trafalgar Square, Wood Green, been to a football match, but still with me though, it’s still as if I feel the same, you know. (PP08)*

Two Participants (22%) spoke about how useful they found the **practical advice and support** given to them e.g. signposting to other services, as this reduced the negative impact of ending the project.

*PA Yes, so I don’t feel that she’s left me at all, you know, it’s kind of just given me some, yes, signposting, that’s a good... way forward.*

*PA She gave me some good... she gave me some good numbers, as well, before she left. Can’t remember what it was. As usual, I write it in some scribbley way. Oh, yes, the Women’s Resource Centre, that’s it. The Women’s Therapy Centre. They give, like, massages and stuff, and Lifelong Learning Centre. She was talking to me about dissociative order - disorder, which I’m not sure if I’ve got or I don’t. But that was useful, the fact that she’s very knowledgeable. (PP03)*

**6. Programme set-up**

**6.1 Frequency of sessions**

The four participants who completed all their sessions with their peer support worker spoke positively about the frequency of sessions (once a week for an hour). All four participants (44%) said they were happy with this. One participant was only able to speak to their peer on the phone but managed to do this at least twice a week which she felt was enough.

**6.2 Programme duration and number of sessions**

Of the six participants who completed all sessions over the ten weeks, five participants (56%) felt that the duration of the programme was too short and would have liked more sessions with their peer as they felt they would have benefited in a more long term way from a longer intervention.

*PA You know, she’ll sit down and she’ll draw a few pictures, and show me the way, like show me things that are going to be up and good for me in the future, and things like that, so...yes, it’s really great. If I be around someone like that, like, for the next year or something like that, I know I’ll really, really, I’ll be really strong, and I just want, like, even if I don’t see her again in the future, I would love to see her, and to give her a really good thanks.* (PP04)

IV Do you know how many meetings you've had altogether?

PA No, but I didn't feel it was enough. I was quite disappointed with that.(PP01)

One participant (11%) thought that the number and duration of the programme was acceptable and that the programme came to a very natural end within that time.

PA *Well actually, we ended so naturally that I said that it was a godsend that you had made it ten weeks really. But I was amazed at how it ended. It ended on a crescendo, as it were. You know, it didn't end like a whimper. It ended with a crescendo. The best truths were the last. The very best of my… of the thing that inspired me and is inspiring me, anyway you know, was for the last, anyway you know. It couldn’t have been any better*. (PP10)

One participant thought that having the short term support made her appreciate it more and try and get the most from it.

*PA I think the fact that it’s the length of time that it was is very, very good, because it make you think, okay, I’m not... I need to really cherish this because I’m not going to have this for long. But I think it’s an extremely good idea to have this after a crisis if somebody can make use of it, because it reinforces everything the crisis team have done for me over the years, which has been fantastic.* (PP03)

**6.3. Length of sessions**

Seven participants (78%) explicitly said that the sessions generally lasted about one hour and thought this was an adequate length of time. Two participants (22%) spoke of having double sessions (two hours) in one week and finding this particularly valuable as it allowed them to discuss issues more deeply.

*PA*  *I’ll tell you what was helpful was actually seeing her for two hours sometimes. So we could really get to grips with it, so that counted as two meetings, so I think the length is very good. You know, I think it works. And, you know, again, [my PSW], I mean, she seemed to have a very good handle on what would help and what she was there for, instead of to just... you know, when she said, well, I do it for two hours, I was like oh, okay, all right, so we really, you know, got down to the nitty gritty of this*. *(PP10)*

**6.4. Location of sessions**

The locations of sessions varied amongst participants and their peers, decisions made about which locations to choose were reported by participants to be joint decisions. When asked about the location of sessions, four participants (44%) explicitly spoke of feeling able to meet in the location which they felt most comfortable with/ suited their needs.

IV *And did you kind of make suggestions about where to meet or did [PSW name] make suggestions about where to meet?*

*PA I think both. She usually would ask me where I want to meet. Yes, I think we both. Because sometimes she would suggest, like with the Angel thing too, she suggested, sometimes she suggested places and I messed up and I didn't come….And sometimes I suggested places, so it sort of… we decided together*.(PP01)

*IV And were all your sessions with [PSW name] here, or did you go out and about at all?*

*PA Here. Right here. I preferred that. I told him the reason why I didn't want to go to a café, or a library or something, a public place, I really don't want anybody to hear what I want to say about my private life, really. (PP10)*

**7. What was helpful about the programme?**

**7.1. Having someone**

When participants were asked what they found most helpful about the programme, 4 participants suggested that simply ‘having’ someone was the most powerful aspect of the recovery programme. Someone they could talk to at any time if they needed it seemed to be crucial to them feeling well supported.

*IE So, it’s a greatest thing to have her, it’s really great to have her, because I just think, I don’t really look for her to do much more, because what she do is enough, like, you know? Just knowing that I got someone that I can phone, whatever time I’m down, whatever time I feel like I want to take my life, and things like that, and that’s the greatest thing for me….. an understanding voice at the end of the phone (PP04)*

*IE I appreciated the support she gave me, somebody at the end of the phone. I mean, she phoned me up, like I said, in the hospital and she said, get well soon, yes, to see you soon, and that. And that from a stranger was so nice to hear, that somebody cared about me without even knowing me, do you see where I’m coming from? (PP11)*

*PA And it was a continual dialogue between us from the very start to the finish, animated, interesting, deep, comprehensive and in every way helpful, every way helpful. (PP10)*

Two participants noted the difference in the support provided by their peer compared with other MH services, they commented that the warm/supportive approach was quite different from the way in which they see support from these other services.

*PA She was very loving, listening, caring, dedicated, reliable, and she was wonderful in listening. Much better than the people from Crisis.<(PP01)*

*IV Was it helpful, what you covered in the session?*

*PA Very. I mean, the whole idea in [service name] is to talk but the way they, it’s the way they treat you, it’s, I can’t really explain but someone like [PSW name] has come from a different angle and not, I’m here to treat you so, kind of thing.*

*IV So you’re saying that [PSW name] treated you like a person and not like...*

*IE Yes. Basically, yes.*

*IV ...I don’t know, more of an object?*

*IE That’s it. .(PP09)*

**7.2. PSW understanding/ role modelling**

When describing the benefits of having this support, the majority of the participants focused on the ‘peer’ element of the support: Three participants (33%) felt that being able to discuss and share similar experiences benefited them greatly and often described hearing their support workers own personal stories as inspiring and empowering.

*PA ..the way she disclosed it was very, very, you know, very caring but very professional and very thoughtful and it was, I don’t know, it was great, you know. And it’s nice to see someone who, you know, who has... who is recovering very well from symptoms that we both share, you know, and…because one of my things is about worrying about getting back to work or having a relationship or something and we were able to talk about that. (PP01)*

*IV And did you find it helpful that he told you that he might have been through?*

*PA Yes, it did really; because it means you... he might understand me. Because if he’d been a bit ill himself he’d most probably understand, you know. People that haven’t been ill don’t really understand mental illness, you know. It’s like if you break a leg, you might say, oh, I broke a leg last week or... If you say, well, I’ve... I’m on pills and... I’ve had a, you know, I’m on medication and I’m not very well mentally, it’s like... it’s a different subject.* *(PP08)*

**7.3. No judgment**

Four participants (44%) said that they never felt judged by their peer worker and this allowed them to open up more.

*PA And with [PSW name] coming round, I knew she was coming round, I knew she was going to ask me questions, but it wasn’t forced, it wasn’t forced and it wasn’t other people judging you or giving their little two pence worth when they don’t really understand (PP09)*

*PA She was very accepting, so she would accept you without judging, and she would look for things that would… that I would like, not force me to think that I wouldn't like them. But especially she was very listening and she, in a way, inspiring as well. (PP01)*

7.4. Informal nature of support

Two participants (22%) valued the informal nature of the support as they found it easier to relax and share their thoughts.

*IE But this is a brilliant idea, actually, to do it this way. Even the part… It's not in any hospital or anything like that, it's more very casual. Yes. It's a really, really good programme…Because it helps you to open up. Well, this also depends on the person, but [PSW name] was fantastic and she did help me a lot (PP01)*

**8. What was unhelpful about the programme?**

When asked specifically what was unhelpful about the programme 7 participants (78%) said nothing at all was unhelpful, one participant commented that the short term nature of the support can be unhelpful in his recovery, after this type of support ends, it can leave him feeling ‘back at square one’ which puts him off taking part in these types of programmes.

*PA I don’t want to just go around in circles, you know, having talks with different groups of people and then feeling the same weeks after, you know. I mean, I don’t see the point really in going on with these sort of things really, because, I mean, just... Because I can’t explain to you, because I’m not a doctor, I’m only a patient, but having a breakdown is... well, you’re never the same really afterward. (PP08)*

One participant thought that the entire programme was unhelpful for him. He felt that the purpose of the programme was unclear and he did not see what the PSW could offer him that he couldn’t receive from a friend.

*PA Well, to be honest, it was just… as I said, it was like… no, there was nothing she could help with because there was nothing to… I could go to my mate and get the same from my mate, or whatever, so it was just like… you know, it was pointless for me. (PP07)*

**Support wanted was not provided**

The support/help that he felt he required was not support that he felt the PSW could give him. He expressed that because he had a PSW that was why the crisis team would no longer help him.

*PA I don’t know, because I was on medication still and I said, I won’t be able to get any, and it was like no point talking to her because she couldn’t help me with that, and because I was talking to her the Crisis Team… well, actually, you can ask her, I phoned up and said they’re not helping me no more, so it was like talking to her actually put me… didn’t help me at all because I was using something else and the Crisis Team wouldn’t help me no more because I was using someone else, sort of thing, and they actually said they weren’t helping me. (PP07)*

**9. Ways to improve programme**

91. Increase flexibility of sessions and workbook use depending on individual need.

When participants were asked if they could think of anything that would improve the programme, two participants focused on the value of increased flexibility within the intervention both with regards to length, timing of the intervention and content of the workbook.

*IV That's good. I'm really glad to hear that. Is there anything we can change about it to make it better, the programme?*

*PA I think probably it depends on the individual, because I can say that I would need more sessions, whereas someone else may not, so probably it would be more on an individual basis. Possibly I would suggest to make it like flexible, in a way, so that you assess a person's situation on an individual basis, and, depending on the state of mind and need. (PP01)*

9.2 Mindful matching of peer support worker to participant based on common interests.

The participant who did not find the programme useful felt that an improvement which could be made is the matching of peer workers to participants based on common interests and mutual understanding.

*IE I suppose if you can… develop people more, get people more suited up to people, like workers with people who are more suited to them, that person, sort of thing….It was just basically… I think you just need to map people up more for like basically what they can… like, stuff in common, or something like that, and that could be something you could look into, or something.. (PP07)*

9.3 Increased assertive engagement.

One participant felt her experience of the programme could be improved if her peer support worker had made contact with her to let her know she had arrived at her house as she slept through a number of appointments.

*IE Yes, I just, she, I don’t think, she just didn’t ring me when she was outside or, I don’t know if she had her phone or not, but if I had, well I was being, I was, obviously I was sleeping. But the first time she came she had her phone and she rang me when she was outside, which is what most people have to do to.. (PP09)*

9.4 Acceptability of follow up support groups and/ or internet based self- management programs

Generally, participants expressed a reluctance to engage with follow up support groups saying they didn’t think it was for them and preferred one to one support. Similarly, the majority of participants did not think internet based self-management programs would be something they would make use of.

*PA I tend to be... back away from stuff like that, really, because I can’t process it. I look at it, and I just say, oh, my God, because you know the Web, it just gives you about a kagillion things to look at, you know, and I just [unclear] I can’t look at that, at all. (PP03)*

One service user did express interest in the workbook/other resources being available online and said she preferred typing to writing but would not want to lose the actual workbook.

*PA Yes, that's a very good idea. I actually would like it online, the book.*

*IV Would you have preferred it online rather than paperback?*

*PA Yes, probably now, yes, because I am… Well, I used to write a lot by hand when I was little and all that, but now I think everyone types rather than… Although, on the other hand, it's nice to have a version like this as well. Maybe smaller. If it would be available online then you could make smaller formats so that it's more handy. You can put it in your bag and actually have it with you. (PP01)*
