# Supplementary material for: Development of a peer-supported, self-management intervention for people following mental health crisis
Source: BMC Res Notes. 2017 Nov 9;10:588. doi: 10.1186/s13104-017-2900-6 (PMC5680762; doi:10.1186/s13104-017-2900-6)
Supplement: Supplementary file 5 — Additional file 5: DS7. Feasibility testing (stage 4)—main themes from PSW focus group. [file 13104_2017_2900_MOESM5_ESM.doc]

**DS7: Stage 4 Feasibility testing: main findings from Peer Support Workers’ focus group**

Results are presented in four primary themes of; 1) Programme benefits; 2) Programme Challenges; 3) Sessions with peer participants; 4) Information sharing. Figure 1 presents these primary themes and their corresponding sub themes.


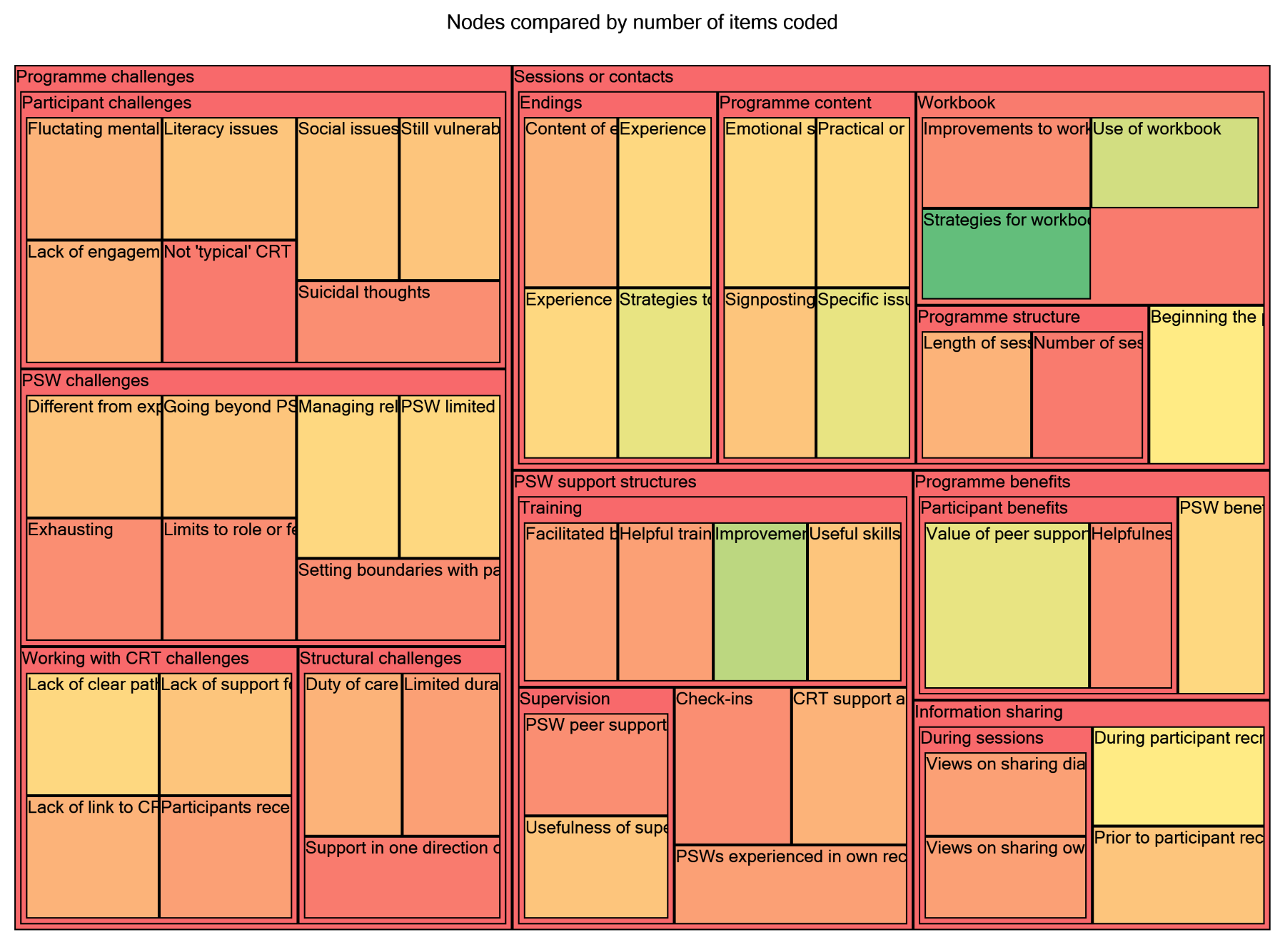


Figure 1. Primary themes, themes and subthemes by number of items discussed

**1. Programme Benefits**

The PSWs highlighted that undertaking the programme resulted in benefits to both themselves and the participants. The programme benefits are presented in Table 1.

Table 1. Programme benefits

| ***Item*** | ***References*** |
| --- | --- |
| *PSW benefits* | *6* |
| *Participant benefits* | *10* |
| 1. *Helpfulness of sessions* | *2* |
| 1. *Value of peer relationship* | *8* |

***1.1. Participant Benefits***

The PSW indicated that direct benefits to participants related to i) the value that the peer relationship provided to participants; and ii) the specific sessions with the individual being helpful.

1. Value that the peer relationship

The value of the relationship related to humanness that developed peer to peer:

*You know, it is that contact, that knowledge that there are two human beings making contact and not two machines making contact. FR2*

Furthermore, the peer support process and the specialness of the peer to peer relationship, allowed the individual a comfortable space where they could share sometimes very deep and personal information. The PSW reflected that this was an advantage of peer support in the mental health system:

*I found my way around that but it was the depth at which they were sharing and I think maybe that was one of the advantages of peer support where people actually feel the confidence to be able to share very quickly very deeply. MR1*

For the individual, an additional benefit to the relationship was that they now had available someone that cared and would provided them with much needed support:

*FR2 I had the comment that I kept him alive. Now I don’t think I kept him alive. I think what I did was show that I cared. And I do care. I know what it’s like.*

Be able to understand and share due to the PSWs lived experience:

*It helped me in the area of, kind of, self-disclosure because if somebody, kind of, mentioned - I’m trying to think if somebody’s mentioned they were bi-polar – but if they did then I can, kind of, go back and say to them, well, actually I understand exactly where you’re coming from because, you know, that’s the particular diagnosis I’ve been given myself and I can understand about depression because that was, kind of, what they first thought it was. And then, kind of, if it’s schizophrenia, well, actually, although I don’t have that I’ve had elements which I can recognise and I can immediately give back to them. MR1*

Which was seen as a core part of the peer support workers’ role:

*FR3 Can I just add one thing? I think it is so essential that we give of ourselves.*

*FR2 Oh, yes.*

*FR3 I think it can’t work unless we give of ourselves and give a lot of our experiences and our understanding.*

Which could be both important and meaningful to the individual and be different to what health professionals offered:

*FR2 But I think that the only time that I could say that it resonated: one of the people has repeatedly gone down so low and has talked about things and ways of killing himself and so on and I just said… And his concern was that nobody, you know, none of the professionals, whether it was the crisis team or anybody, really took that on board, really heard what he was saying or believed him. And that was the only time, I think, with my own reflections on myself that… I didn’t go into any graphic detail just that I actually knew where he was coming from; different but I knew. And that, I think, resonated for him.*

It also had the potential to break down stigma:

*You know, if I can’t talk about it how can I, kind of, get… If anybody, kind of, has stigma issues the only way, to me personally, to break down that barrier is simply to be as open as you possibly can and that’s the way you crack stigma.* *MR1*

Knowing what it is like:

*I’ve been in the position of people not listening and ignoring me and lying, blatant lying, until I could prove that they were absolutely, absolutely wrong. And it just… It [the Peer Support relationship] was open. It was open.*

1. The helpfulness of sessions was highlighted on a number of occasions. The sessions were considered useful as they:

Assist the individual to communicate with others in their support network:

*I’d pass them on to a support worker and that person will have written down the things* *that they had expressed as important just because they might not remember them. FR2*

Hold the individual at the centre of the session support to direct working together on the individuals life issues:

*And it’s how do I give of myself as early as I can in the process and how do I, kind of, hold onto it’s the individual that’s important. Well, it’s about where are those things getting in the way, where are those issues actually causing issues in life which maybe, working together, we can help. MR1*

Build confidence towards self management:

*I was there only to help them have something at the end of the ten weeks to build up their self-confidence so they had a support structure away from me once the ten weeks were up.* FR3

Boost self-esteem:

*So I started looking at his strengths with him and wanted to think what there was that, you know, that he thought he could do and what he thought he was valuable as. FR2*

Assistance with practical issues

*Because I did work with them on benefit issues, sorting out, doing a budget. FR3*

Connecting with community resources

*The housing people phoned me and said he was having difficulty doing… sorting out council tax or something and I thought, I’m sure he is, you know, he can’t read the pieces of paper let alone anything else. And they, sort of, said, what did I think? And I said I thought that, you know, yes he needed somebody and I was leaving. I wasn’t going to be around. So he did get somebody who was lovely and who actually came to court with him FR2*

Access support when the individual was at risk

*He was suicidal or he was expressing… he was talking about ropes, he was talking about… you know, and I can hold somebody but only while I’m holding them, if you know what I mean. I can’t be there. If I can’t go in there and whatever, I can’t do anything. So I phoned them up and I got… There was a duty person and I thought, wow, there is somebody here, that’s a good sign. And she did phone him and arranged for somebody to… and then they phoned him again on the Monday, FR2*

Looking at goals

*But actually we did actually start at the positive side which is goals and dreams which I always think is a good way to start, really. MR1*

Engaging with the community

*So, you know, there’s a positive thing but the other thing on goals and dreams he had a couple of… we had a goal, go to the centre of London, so we did it…. I was quite humbled in a way that, you know, I hadn’t realised he hadn’t been on the tube for so long. So he saw Buckingham Palace, we had a little walk in Green Park and then, kind of, went back on the tube. And I think, you know, that’s probably one of the things, hopefully, he’ll remember because it also helped him realise, I think, that he could actually use the tube, that possibly it wasn’t quite as much of a challenge as he thought. MR1*

Motivation and support

*FR1 Okay. Motivate. Motivate. Motivate.*

***1.2. PSW Benefits***

Being honoured to share in people’s lives:

*So the emotional side, I think, was more than I had anticipated, the depth for which people were saying things to me was deeper than what I expected it to be and I felt really honoured for that, I thought, for the amount that people were sharing.*

*FR3 And very moving.*

*FR2 Very, very moving. And you learn things about people and their lives and what’s made them the person*

Felt like it was an opportunity:

*FR2 I think it was a wonderful experience.*

*FR1 Yes.*

*FR2 I went in there saying that to have the opportunity to have ten sessions and, okay, we might argue about that, but that’s what it was, ten sessions to actually go and spend an hour with somebody and just work with them and understand is the most amazing experience.*

An enjoyable process

*MR1 Yes, I mean, it’s just that we, kind of, started out with it. You know, it’s been an enjoyable process.*

Feeling like they (PSWs) have helped the individual:

*MR1 You see, I think you’ve done a fabulous job, I think, as we all have.*

Bonded and worked together as a team:

*I found that almost one of the best parts because it bonded the group together and I, kind of, felt confident with people. FR2*

Supporting each other as PSWs

*we actually had each other who supported us as well. FR2*

**2. Programme Challenges**

The PSWs highlighted that there were challenges to programme delivery. Main themes that emerged from the discussion included: i) challenges to the peer themselves ii) challenges relating to the participant iii) structural challenges iv) challenges working with the CRT.

The programme challenges are presented in Table 2.

Table 2. Programme challenges

| ***Item*** | ***References*** |
| --- | --- |
| *PSW challenges* |  |
| 1. *Exhausting* | *2* |
| 1. *Different from expectations* | *5* |
| 1. *Managing relationship intensity* | *6* |
| 1. *PSWs having incomplete skill or knowledge* | *6* |
| 1. *Setting Boundaries with participants* | *2* |
| 1. *Limits to role or feeling it is not enough* | *2* |
| 1. *Going beyond PSW role* | *5* |
| *Structural challenges* |  |
| 1. *Limited duration of programme* | *3* |
| 1. *Support not bidirectional* | *1* |
| 1. *Duty of care and risk issues* | *4* |
| *Challenges working with the CRT* |  |
| 1. *Lack of support for participant* | *5* |
| 1. *Participants receive support* | *3* |
| 1. *Pathways back to CRT* | *6* |
| 1. *Lack of link to CRT team* | *4* |
| *Participant challenges* |  |
| 1. *Still vulnerable or unwell* | *5* |
| 1. *Fluctuation mental state* | *4* |
| 1. *Not typical CRT service users* | *1* |
| 1. *Suicidal thoughts* | *2* |
| 1. *Literacy issues* | *5* |
| 1. *Difficulty with or lack of engagement* | *4* |
| 1. *Social issues* | *5* |

***2.1 Challenges for the Peer Support Workers***

Two PSWs referenced that the support was quite exhausting compared to previous work that they had done and it was different to what they expected.

*It’s been a big learning curve. It’s just… For some reason I expected it to be very similar to mentoring and it’s not and it’s just so much more exhausting and draining than informal peer support FR1*

Many found the intensity of the relationship difficult to manage:

*They were far too needy. FR2*

*So I enjoyed it but, yes, emotionally more challenging than what I had anticipated. MR1*

This often resulted in the PSW finding it difficult to set boundaries around how much support they could provide, and often went beyond the roles job description and requirements:

*Because it’s very much about you being there, being there for that person and giving of yourself and, for me, I think I needed some sense of boundaries about how much of yourself do you give. And just in terms of… Like, you’re not just there for the person when you see them but you’re there for the person of an evening or a weekend if they need you. And I had one person that needed me a lot so I spent, like, a very, very long time on the telephone giving support. FR1*

Some PSWs suggested that the limits to role left them feeling that there was not enough support for the individual:

*But it highlighted a need. People needed that one to one with someone and that’s why I think the relationships just got so deep and intense. FR1*

*the other person seemed to have nobody, although had a partner, but seemed, in a sense, to have nobody. FR2*

Some of the PSWs also recognised that they had incomplete skill or knowledge and felt that this needed further training and development.

*some of us, I think, went into situations that we were not really equipped to… But, overall, it was a reasonable… And it was a challenging experience but it was very, very exhausting as well. FR3*

*So it wasn’t real enough about working with people and there were too many bits that were repetitive and you just think, well, okay, but it just leaves you wanting a lot more; not just a little more, I think, a lot, a lot of gaps. FR1*

Furthermore, support was also less bidirectional than informal peer support, which some of the peers felt was challenging.

*I learnt a lot about myself doing it; but there was none of this, sort of, you know, the jargon, the mutuality of the giving and taking. There was none of that. But maybe there’s a little bit of that. FR1*

***2.2 Structural challenges***

Limited duration of programme was seen as a frustration as there was a large proportion of participants with unmet needs.

*I think we’ve all got that and I think the huge frustration is that in some ways seeing somebody for one hour a week, seeing the huge amount of need which they actually have and then feeling that you can’t actually go out and do a little bit more for that particular individual is… I personally find that really, kind of, frustrating. MR1*

Structural challenges were also raised working with the CRT. This highlighted some of the teething issues of the new programme set up. The PSW felt that there was a lack of support for participant from the CRT. For example, the pathways back to the CRT were a challenge for PSWs. Duty of care and risk issues were raised, particularly around linking back to the CRT of continuity of care for participants, and a lack and lack of additional community based support for individuals in crisis.

*You had somebody who, seriously, should have either been admitted or within the realms of the crisis team and there was no way of getting them back on to the crisis team. FR3*

*FR1 I don’t think they sat down to think, right, we’re going dump these people, we’re going to dump these people on to the peer support workers and we’re not going to, like, be there for people in crisis. I don’t believe they did.*

*FR2 No, no, no.*

*FR1 I think they probably thought their system would work and it failed.*

*FR2 And they thought probably that the GP would pick up the flack.*

*There’s no continuity. FR1*

***2.3*** ***Participant Challenges***

Participant challenges were also raised. The PSWs felt that and participants were still vulnerable or unwell at the time of referral. Participants also had a fluctuation mental state and/or suicidal thoughts which could be an ongoing challenge for PSWs when providing support.

*I think what we’re saying is that the level of unwellness was great. FR2*

*He was suicidal or he was expressing… he was talking about ropes, he was talking about… you know, and I can hold somebody but only while I’m holding them, if you know what I mean. FR2*

In addition to mental health issues, the PSW raised that the social issues that the participants were facing especially around housing, family, benefits or alcohol issues, were impeding participant recovery.

*She was also suffering from alcohol problems and we looked at other agencies as well as we were talking about the book. She went to AA. FR3*

*The housing people phoned me and said he was having difficulty doing… sorting out council tax or something FR2*

*And so, for example, she talked about falling out with her family over a psychotic episode. FR1*

Some PSWs raised that there was some difficulty with engagement for participants, they suggested that the social and mental health issues may be a factor that impacted upon this.

*Because I think although my first person was really intellectual, she engaged with the book at the beginning, because her life it just took over, she just thought, pffft, it’s got nothing to do with my life doing this book. FR1*

*the second person I had he wasn’t able to maintain it because he went away for whatever reason. MR1*

*one who I never met who was very happy to talk on the phone but never actually, I never actually, met up with her, who seemed to have an enormous number of problems, all sorts of things going on in her life FR2*

The PSWs suggested that the participants may not be typical CRT service users, this was because a large proportion were not under case management rather were just accessing their GP for mental health support.

*An interesting thing came up in a meeting yesterday. It was that, apparently, the Camden/Islington, their statistics are that 80% of people actually have a care coordinator who leads the crisis team. And then we were talking around the whole issue, well, okay, then why does it mean that everybody who we were seeing didn’t? MR1*

Literacy issues were also raised as a challenge when using the workbook.

*But my second person, I think, the issue is that… I think he’s got problems writing... His writing’s very, sort of, like, primary school. But I don’t know if that’s because he comes from another country or… I don’t know. So it’s just slow getting through the book. FR1*

*So there’s lots of difficulties in terms of difficulty in reading. FR2*

**3. Programme and session structure and delivery**

The PSWs discussed methods in which the programme and sessions were delivered. Main themes that emerged from the discussion included: i) the individual sessions or contacts and how this was run, ii) the use of the self management workbook, iii) the programme structure, and iv) finishing the support with the participant. The programme and session structure and delivery are presented in Table 3.

Table 3. Programme and session structure and delivery

| ***Item*** | ***References*** |
| --- | --- |
| *Sessions or contacts* |  |
| 1. *Beginning the programme* | *7* |
| 1. *Programme content* |  |
| - *Emotional support for participant* | *6* |
| - *Specific issues covered* | *8* |
| - *Practical or social help* | *6* |
| - *Signposting* | *5* |
| *Self management Workbook* |  |
| 1. *Use of workbook* | *9* |
| 1. *Strategies for workbook use* | *14* |
| 1. *Improvements to workbook* | *2* |
| *Programme structure* |  |
| 1. *Number of sessions* | *1* |
| 1. *Length of sessions* | *4* |
| *Endings* |  |
| *Experience for participants* | *6* |
| *Experience of PSWs* | *6* |
| *Strategies to manage endings* | *3* |
| *Content of endings* | *4* |

***3.1 Sessions or contacts***

The PSWs advised on their experience and strategies for beginning the programme with participants. They suggested that these can be difficult for the participant as they may be very anxious.

*FR1 Yes, exactly. Well, I, kind of, described myself over the phone but I didn’t, sort of, anticipate people being as afraid to meet me, which is silly because [overtalking].*

Location of the meeting must be considered as privacy, and the participants needs should be considered

*FR1 We met on the street. I’ll never do that again. But she didn’t know what to expect and so she’s…*

The PSWs took it as a chance to get to know the participant:

*I suppose I took the first session as being just to chat MR1*

And felt that flexibility in timing was important as it is such a vital session for forging relationships.

*MR1 I think the first session there can be some merits of being flexible over the length of time on that first one.*

*FR3 Definitely.*

*MR1 Because that whole process can take a little bit longer because… and for some indeed it can take shorter. So I think there’s a degree of flexibility needed over that first one to really respond to the individual, you know, but they, kind of, want to hang on for a bit longer because there’s actually quite a lot potentially happening in that first session. And you, kind of, want to take it on as far as, kind of, possible to be able to set the overall picture because if you can crack it on that first session then actually all the rest there’s a fair chance that it will go okay. If you miss it on that first session you’re a bit stuck, really, because it’s more tricky then to, kind of, pick it up in the future. So flexibility is very important, I think, time-wise on that first one.*

Overall, the PSWs felt that “*the first session’s good for that kind of grounding if you get a chance*” *FR1*. Things that PSWs felt were useful to be discussed in this opportunity for grounding included:

- *double-check that actually they understood what they’d signed up for (MR1)*
- *the amount of sessions (FR1, FR3)*
- *The approximate length of time of the sessions (FR1, FR3)*
- *Introduce the booklet (FR1, FR3)*
- *Set the ground rules/boundaries (FR1, FR3)*
- *What they’re going to be left with at the end of the programme (FR3)*
- *At the end of the session to say that you might want to look at the booklet over the next week and we’ll talk some more next week (FR3)*
- *But if you have any questions or queries, call us (FR3)*

PSWs should be prepared for the person potentially revealing a great deal of information about themselves.

*Which I wasn’t expecting on the first meeting and I wasn’t expecting her to make big life changing disclosures to me. I just wasn’t expecting that in the first meeting. I was quite shaken actually by the volume of information I got on that very first meeting. FR1*

The PSWs should also have thought through what they are willing to share about themselves:

*I think you’ve got to be prepared for that and share a little bit about yourself FR3*

***3.2 Programme content***

The PSWs outlined the general session content over the 10 weeks of support. This included emotional support for participant, the specific issues covered, practical or social help and signposting. These items are covered in “helpfulness of sessions” code.

***3.2.1 Self management Workbook***

The use of the self management workbook varied. Some PSWs found it easier to utilise than others which was attributed to the PSWs style of working.

*So I suppose I’ve used, in practice, I’ve used the principles but I haven’t used the booklet but I’ve tried to get still to get people to write something down after. MR1*

*[other PSW] you’ve done the booklet with everybody, haven’t you? FR2*

This was attributed to both a need for flexibility in working as people’s lives are not linear and that certain areas to a person in their life may be needing additional focus.

*I mean, to me, the most exciting thing is actually… well, if the boundaries are extremely loose and everything because actually I think then you can work best with the individual. But the reality is, you know, we’ve got to be real about this. We can’t, kind of, operate in too flexible a way but we need to operate sufficiently flexible so that the individual MR1*

*I suppose I went beyond what was in the booklet. FR3*

Some PSWs did not use the booklet and attributed this to working with the individuals needs and wants.

*And the final guy, yes, I just couldn’t get him to use it at all and pretty much gave up really very quickly because he clearly didn’t want to touch it with a barge pole. MR1*

*my first person was really intellectual, she engaged with the book at the beginning, because her life it just took over, she just thought, pffft, it’s got nothing to do with my life doing this book. It isn’t going to sort my life or my problems out. FR1*

Strategies for workbook use were recommended by the PSWs. These included:

Making it part of the weekly routine:

*We always did something in the book every week. FR3*

Constantly referring to it as relevant topics come up

*When we were sharing things with each other then if there was something that came up that was in the booklet we would then go to the booklet and do the exercises in the booklet. FR3*

Breaking the booklet up amongst other activities

*when she was writing some of the time she was hearing really bad voices so when that occurred we would take a break, she’d make a cup of tea or whatever, and then we’d come back to it and I would offer to write or whatever seemed appropriate at the time. FR3*

Using the sections that were personally relevant to the individual

*And so, for example, she talked about falling out with her family over a psychotic episode. Well, in this booklet, there’s a place there that says about getting in contact again with people or saying sorry or… FR3*

Breaking the goals down into small achievable steps so as to see improvements and changes of the short period of support.

*Because I was thinking, we were doing the book; every week we were doing aspects of the books, particularly his goals and we were going places for him to meet his goals. But I think one of the things that I find frustrating about the goal setting is that you’re never going to see it through to conclusion FR!*.

Scribing of drawing for the individual if they had literacy issues

*I left my pages that they could then add to their book, that’s one way of completing the book FR1*

*But I did use the book, but not use the book, because it was absolutely impossible to use something that was as it is, with lots of words which really had no meaning. So, did you see all my wonderful drawings, trying to put things down. FR2*

Improvements to workbook was also suggested by the PSWs. These included:

The wording was challenging for some of the PSWs, for example, in one section, apologising to others for what one id when they were unwell was seen as something that could be improvised in terms of its language. Instead PSWs felt it could be “getting in contact with people again”

*Yes, saying sorry. I hate that. FR1*

*I didn’t like the sorry bit but getting in contact with people again. FR3*

*Yes. So it’s that language, that, saying sorry; and everybody said, oh, I don’t like that but maybe it could be re-phrased. MO*

Having back up worksheets was also seen as something that would be useful. This may require a place for storage of additional sheets.

*if I’m doing the book again, what I’ll do is photocopy all the pages. and it might be easier to do that. FR1*

Having blank sections where you could add in your own work

*I mean, one, it was more using it in the sense of… to do a bit of creative writing MR1*

Reducing some of the pages to draw ideas together

*in the extra training session which I then, kind of, played around a bit and adapted it and thought, well, maybe it might be a way of drawing together some of the things. MR1*

***3.3 Programme structure***

1. Number of sessions

The number of sessions was only discussed by one PSWs. It was suggested that offering for discontinuation of sessions so the participant could choose whether to continue was seen as useful.

*So, yes, and the final guy, well, that was, kind of, fairly easy because that was just about on session five, I think, I, kind of, re-capped that actually we’d had five sessions. Actually I offered him the opportunity, did he wish to finish it at that point, simply because we weren’t using the workbook at all. It wasn’t clear exactly what he was getting out although I was getting some feedback that he was getting something out of it. He said, no, I’d like to continue. MR1*

1. Length of sessions

PSWs suggested that there was some variation in the length of time that individual sessions should run. For example, it was suggested that the first and the last session generally took longer.

*“MR1 So flexibility is very important, I think, time-wise on that first one.*

*FR1 I think the first and the last session.*

*MR1 And the last one, yes.*

*FR2 Yes, they both need to be longer”*

And a degree of flexibility is needed in order to meet the individuals needs.

*“So I think there’s a degree of flexibility needed over that first one to really respond to the individual, you know, but they, kind of, want to hang on for a bit longer because there’s actually quite a lot potentially happening in that first session.” MR1*

Additionally, the flexibility in using double sessions was useful in order to assist people to meet their identified goals.

*So, you know, there’s a positive thing but the other thing on goals and dreams he had a couple of… we had a goal, go to the centre of London, so we did it. So rather than write it down, we did it. You know, which to me, because that was a fairly easy thing to do with a defined… you know, that could be covered within a session. Actually that ended up being a double session. MR1*

- - 1. ***Endings***

Endings with participants were an important part of the overall experience of the support.

1. ***Experience for participants***

Some participants found the endings quite challenging.

*FR1 You know, my gentleman got drunk on our last meeting…. And I think my people did feel abandoned and I need help with that to make it better.*

1. *Experience of PSWs*

PSW also recognised that endings were an emotionally challenging time for them.

*FR3 But I probably in the end found it harder than they did to end it.*

*It’s quite painful I think to go into someone’s life and, like, fulfil that role; to be the first person to have that, kind of, caring, supportive relationship with someone and not being able to replicate that anywhere and then leave that person. That’s quite painful. FR1*

1. *Strategies to manage endings*

To help manage the emotional nature of endings, it was suggested that constant gentle reminders of the short term nature of the support was seen as vital.

*FR3 Oh, I don’t know if I’ll be able to cope. So I used to reinforce the fact that you’re going to have somebody, you’re going to have these people and the booklet, but also people around you that are going to help you.*

Making links with the community resources and other support networks also helped ensure that endings were handled smoothly.

*One guy was pretty, yes, tricky to, kind of, leave and that was more because there was unfinished business and I could see so much more which needed to be done and actually it appeared that it wasn’t happening. But, I suppose, the way I resolved that one is that I had a meeting with a support worker and I was dead lucky that, yes, she had a support worker. MR1*

If possible having a celebration to end made it a celebratory rather than a sad experience.

*So that was… And I think the idea of having some sort of very different thing on that last day, a celebration, is a good one, really. MR1*

Supervision was vital to help the PSWs process and plan for the challenging time.

*And, yes, I found that difficult but I think, yes, the way we talked about it, that helped and I think then I didn’t blame myself for that, so that was good.* *MR1*

Check if the participant wants to continue:

*Actually I offered him the opportunity, did he wish to finish it at that point, simply because we weren’t using the workbook at all. It wasn’t clear exactly what he was getting out although I was getting some feedback that he was getting something out of it. He said, no, I’d like to continue. We had a further four sessions because then it was coming to the end of November so he was happy with that. I was happy with that. MR1*

1. *Content of endings*

Additional useful ideas in terms of ending were to revisit the workbook.

*FR3 You see, I probably dealt with it in an entirely different way in that we talked about, at the very beginning, the booklet and that we only had ten sessions; and that we would be ending but that they would have this booklet at the end. So in their bad days, if they were to have any when I’d left, they could refer back to the booklet for things that they could do to keep themselves well; the triggers, they could pick them up after. So I wasn’t… If you like, I was there only to help them have something at the end of the ten weeks to build up their self-confidence so they had a support structure away from me once the ten weeks were up.*

Revisiting future goals and dreams

*And we looked back over the goals and the dreams and we found an extra one which was really important FR2*

Revisiting things that had been achieved together

*I said, well, why don’t you just write something about, kind of, you know, how you felt about, you know, what we’ve done together, what you think you’ve achieved, what we’ve achieved together. MR1*

**4. Information Sharing**

The PSWs discussed how information was shared in various aspects of programme delivery. Main themes that emerged from the discussion included information sharing: i) prior to participant recruitment; ii) during participant recruitment; and iii) during sessions. The views on sharing information are presented in Table 4.

Table 4. PSW views on information sharing

| ***Item*** | ***References*** |
| --- | --- |
| Prior to participant recruitment | *5* |
| During participant recruitment | *7* |
| During sessions |  |
| 1. *Views on sharing own experience* | *3* |
| 1. *ii) Views on sharing diagnosis* | *3* |

1. Prior to participant recruitment

Prior to participant recruitment PSWs suggested that they would like involvement from the outset of recruitment.

*FR1 I didn’t like the fact that they’ve had that first meeting on their own and I wasn’t there. And I wasn’t there to hear exactly what she was telling… saying to that person and how she was managing their expectations. And that is something I still feel quite uncomfortable about.*

*FR3 Yes, I totally agree with [PSW name].*

*FR2 Yes, because it’s behind our backs.*

PSWs would have liked more time with the crisis team to understand their role and make communication links as this was something they felt was missing.

*I think we should have had half a day with people from the crisis team to come in and tell us how they see it, how they see it working, how they wanted us to work, where can we meet in the middle, what would happen if we did have difficult cases who needed to be referred back.*

*FR3 I think that I would have liked to have seen more communication between either the research team and the CR teams and between us and the CR teams. I think that would have been just so helpful and that didn’t happen, unfortunately*.

PSW felt that a handover from the crisis team or more information from the research team may also have been useful

*I think there has to be a handover and whether that handover is a handover from… Because when you’re within the Trust there is supposed to be a meeting where you have the person who is handing over giving information and that didn’t happen and perhaps that might have made it safer.*

*FR2 It was different and everybody will have their own perceptions. I think, for me, I was absolutely fine going it was when I got to the front door and the door had been repaired with a… And I thought, what! What am I going into. So that… But the person was absolutely fine and that wasn’t a problem. For the second person I think there was a huge difficulty. I mean, I went to that person’s home and they could have been out but they weren’t. With the second person I didn’t know that there had been difficulty for Beth, it was Beth who went to that one, actually meeting with her for the first time. Now I don’t need information, I definitely don’t need a diagnosis because I… The story of my diagnosis and being given it and having no, you know… There’s no point, it’s just a load of words which mean nothing to me. I know for a lot of people it’s very important But actually we never met. I didn’t know that there’d been a difficulty on the first occasion and I think that if I’d have known that then I would have been prepared for that fact. I mean, I think I tried five times to see her, three of them I actually went. That was the only thing that I think that might have been helpful for me. But I do think that that person obviously was very concerned and very concerned even to meet Beth, but Beth did get there eventually*

During participant recruitment

Some PSWs identified that they would have preferred more information on the individual before seeing them.

*I think in hindsight we possibly could have known more about the people that we were peer supporting*

*I would have preferred some more information because my first participant was schizophrenic, a paranoid schizophrenic, hearing voices, having hallucinations. I would have preferred to have known that before I went through the door. And also my second person had problems with writing and he’d had a brain injury and I didn’t know that either and that would have been helpful.*

However, felt that overall not know some information had it’s benefits.

*I think I was one of the people who originally said that I’d like to have a little more information. I actually found it quite an interesting challenge and actually quite an interesting process of meeting someone I actually didn’t know anything about whatsoever. I found myself, of course, asking questions, just listening and almost trying to work out what a person’s diagnosis was from what they were saying, which is an interesting, kind of, process. But that’s within finding out about them so it’s not, you know, that wasn’t what I was maybe after but actually I found that… I suppose, in a lot of ways, I agree with [other PSW] that actually understanding, kind of, something about a person does actually help you. But I suppose it forced me, because I had none of that, to actually pay a lot more attention within my first session in order to… Okay, I didn’t get it all in the first session because you never do but actually by the end of most of the first sessions. So I suppose out of three first sessions I found I pretty well had a very clear impression of, kind of, you know, the extent to which they wished to, kind of, share with me about those aspects. But I looked forward to knowing a little bit more but I wonder if… The only downside of that is maybe it will make me slightly less, kind of, digging - well, not digging, that’s the wrong expression – but enquiring and, kind of, carefully listening and all of that sort of thing within that first session because a lot of those initial things are there on the plate in front of me, as it were. So I’ve got mixed feelings but I think, on balance, I prefer, I would prefer to, kind of, know more.*

*FR1 I really don’t want to know people’s diagnoses. I’ve not said to anyone, what’s your diagnosis? Because it just didn’t appear… It didn’t seem to be important. You see, what was important to me is the person and their symptoms in terms of how much it’s negatively impacted on what they want to do and getting in the way of their life. That’s important. But I just think in the time I saw people I knew more about them than the crisis team would ever know.*

During sessions

1. Views on sharing own experience and diagnosis

Some PSWs found talking and sharing their experience vital

*FR3 Can I just add one thing? I think it is so essential that we give of ourselves.*

*FR2 Oh, yes.*

*FR3 I think it can’t work unless we give of ourselves and give a lot of our experiences and our understanding.*

And that PSWs needed to be prepared to talk with people about their experiences.

*FR3 I think you’ve got to be prepared for that and share a little bit about yourself;*

It was suggested that when using the self management booklet, PSWs sharing information could help facilitate discussion.

*FR3 When we were sharing things with each other then if there was something that came up that was in the booklet we would then go to the booklet and do the exercises in the booklet.*

Some PSWs found talking and sharing personal information about diagnosis useful, however, the process needed balance.

*MR1 I mean, I don’t think I ever named the diagnosis to the individual but actually when I recognise symptoms which I recognise within myself I acknowledge that I have actually had those symptoms. So I suppose actually I’m probably still quite close to the approach you’re, kind of, taking but I… I don’t know. I mean, it’s, kind of, a real dilemma, I suppose. It helped me in the area of, kind of, self-disclosure because if somebody, kind of, mentioned - I’m trying to think if somebody’s mentioned they were bi-polar – but if they did then I can, kind of, go back and say to them, well, actually I understand exactly where you’re coming from because, you know, that’s the particular diagnosis I’ve been given myself and I can understand about depression because that was, kind of, what they first thought it was. And then, kind of, if it’s schizophrenia, well, actually, although I don’t have that I’ve had elements which I can recognise and I can immediately give back to them.*

*Because, with me, kind of, that’s very much my approach. I very much give a heck of… you know, I virtually lay it all there on the plate really in front of people because that’s just the way I come at it really. And hearing things about the diagnosis, or symptoms, because nobody actually turned around to say, well, I’ve been diagnosed as… they’re more talking about, well, you know, I felt depressed or, you know, I’m, kind of… I actually think probably one person did mention issues of bi-polar. But I think it enables me to, kind of, share but, yes, it would be a bit odd having it straight away because, I suppose, in a lot of ways I go into the first meeting wanting to lay it all out on them and say, oh, yes, I recognise it. So I can understand exactly, kind of, what you’re saying. That’s why I’m very split, a split personality on that, whoops, on that particular issue. I really… I like the challenge and I like… And it’s how do I give of myself as early as I can in the process and how do I, kind of, hold onto it’s the individual that’s important. Well, it’s about where are those things getting in the way, where are those issues actually causing issues in life which maybe, working together, we can help. MR1*

**4.1 PSW support structures**

The PSWs discussed the support structure that were in place during the programme. Main themes that were raised in the discussion included: i) training; ii) PSW experience in own recovery; iii) check ins; iv) supervision; CRT support and link important. The views on the PSW support structures are presented in Table 5.

Table 5. PSW support structures

| ***Item*** | ***References*** |
| --- | --- |
| *Training* | *21* |
| *PSW experience in their own recovery* | *3* |
| *Check ins* | *2* |
| *Supervision* | *7* |
| *CRT support and links important* | *4* |

1. Training

The training gave PSWs a good sense of bonding with each other as a team.

*FR2 But that was what was so good. You know, you said about bonding.*

The training gave participants tools to work with

*FR2 There were bits of it that I quite liked and I liked different tools that people used so, for me, that’s always an interesting thing to pick and to learn from*

Improvements for training included the need for a greater focus :

- on actual real world challenges that have been faced as PSWs/ learning from past experiences (*FR3: I think we could have heard more of their own experiences; when they were doing peer support what sort of challenges did they come up against and that was never given.)*
- learning skills to deal with challenges that may arise whilst providing peer support (*It’s one of the greatest things is your own life experiences and all the other skills you’ve picked up through life and actually if you’ve got all those actually peer support’s… the difficult elements is more how to deal with the practical, difficult problems and that’s where, I think, the training should be*)
- boundaries of the PSW role (*And I think one of the frustrations is that when you see something that you know that you could help with that, because you have the skill to do it, it’s knowing when to say, no, I’m not actually going to do that.*)
- Utilising the available resources (*FR2 I find that this particular one is probably the highest, if you like, because it’s outside of the trust structure; it’s on your own, working with somebody on your own, having to find information when information isn’t out there, when you phone up and you keep getting this answer phone and nobody phones you back.*)
- Having a greater understanding of the various types on mental health conditions and their management strategies (*MR1 I think the other missing bit, to me, was something around the whole issue of, I suppose, kind of, knowledge about different conditions and medication which… and I got a certain amount of knowledge on that.)*

1. Check ins

Check in processes were seen as useful

*FR3 The back-up from, like, when we went to the appointment and phoning in, etc, I thought that was fine.*

Especially as it provided on the spot supervision and debriefing after the session

*MR1 So that’s that dynamic. I think it’s worked well because we’ve known each other. Well, I think the fact that there’s an opportunity after each to almost have a quick bit of supervision as well, because although I never took the opportunity over the phone when I was reporting in there would have been the opportunity and clearly, you know, if I’ve raised an issue then it would… an opportunity would have been made for me to talk about it if necessary.*

1. Supervision

Supervision was seen as a vital part of the support for PSWs

*FR3 I think they were just immensely important. I don’t think we could have done the work we’ve done without them, to be honest, without getting together like that and sharing our experiences and knowing that somebody else who’s doing, who’s a peer supporter, has experienced the same problems and the same emotional things that you’ve experienced. I think that was essential.*

Supervision was seen as a place for bonding the PSWs and fusing the team dynamic

*I found that almost one of the best parts because it bonded the group together and I, kind of, felt confident with people.*

For working through challenges together and not feeling alone in the work

*But, I mean, the supervision, the important bit for me was actually about us sharing and talking and, as with anything, that you know that actually it isn’t just you.*

For working through and managing the tension of balancing the research and the support

*I talked about probably the best part of 20 minutes, half an hour even. And so I think some of those thought processes which were going on in my brain at that time and I think it was also partly I was also very busy outside of… And so my brain was just too full up and so it was very helpful to have that additional resource to call upon to give her that, kind of, perspective and the dynamic about research versus ethical issues about making sure the research isn’t totally messed up but then making sure that the individual is helped as much as possible and the tensions which can be within that. .MR1*

1. CRT support and links important

Fostering good support and links with CRT was seen as vital but was lacking at the time of the pre-pilot rollout. This is described in 3.2. programme challenges (challenges working with the CRT)

**SUMMARY OF FINDINGS**

There were noted challenges that were faced by participants engaging in the programme who were often seen as still vulnerable, had fluctuating mental states, continuing life issues and social issues (e.g. housing, substance misuse). Despite this, PSW reported that the support seemed helpful to individuals who participated, many met some of their recovery goals and overall they placed a high value on the peer support offered.

The PSW reported that being a part of the programme was highly rewarding however, there were challenges identified particularly as it was different from their initial expectations, for example, there were some difficulties managing the relationship intensity with the peer and their need for support. This raised challenges with setting boundaries as often wanted to go beyond the PSW role.PSW felt that they has some limitations to their initial skills and knowledge before commencing especially around fully utilising the structured self management workbook. This was particularly difficult as many participants had literacy issues using the workbook. PSWs felt that supervision was a vital support to help them tease out these challenges that were faced and improvements to training were suggested. They did report that training was particularly helpful for bonding the team and overall PSWs reported excellent team dynamic and support between each other.

Structural challenges that arose included the limited duration of programme as participants wanted more time with the peers. Flexibility of the intervention delivery, without compromising the research integrity was called for. Strategies and content for sessions was highlighted and will be useful for the next phase (Pilot). Concerns around duty of care and risk reporting were raised as the CRT was no as heavily involved as initially thought. Overall, there was a need clearer pathway back into CRT for participant if needed. It was suggested that strengthening the PSW link with CRT teams was required.
