# Supplementary material for: Development of a peer-supported, self-management intervention for people following mental health crisis
Source: BMC Res Notes. 2017 Nov 9;10:588. doi: 10.1186/s13104-017-2900-6 (PMC5680762; doi:10.1186/s13104-017-2900-6)
Supplement: Supplementary file 6 — Additional file 6: DS8. Pilot trial (stage 5)—main themes from participant interviews. [file 13104_2017_2900_MOESM6_ESM.docx]

**DS8: Pilot trial (stage 5) – main themes from participant interviews**

Follow-up interviews were completed with 18 out of the 21 (86%) of participants who were randomised to receive PSW support. The other 3 participants declined a follow-up interview.

**Attendance and barriers**

Not all participants offered PSW support met with a PSW and those who did meet did not attend all the sessions, due to various engagement barriers. Seven participants (39% of those interviewed) reported attending all ten sessions. Two participants were still seeing their PSW at the time of the interview and intended to continue all the sessions. One participant reported attending most of the sessions but couldn’t recall the exact number they had attended.

Two participants only attended one session. One of these participants did not like the idea of weekly meetings:

MP5 “*I didn’t mind the one meeting, that was okay, but I just didn’t fancy getting myself, kind of, stuck with having to do it every week*.”

The other participant had one meeting with her PSW who became unwell and was unable to meet up with her again. This participant reported that she was offered another PSW, but not until much later and she declined this offer, as she was worried that she had said something to the first PSW which caused her to become unwell:

MP25 “*Well, when I met her she seemed promising, and she promised that she’d do quite a lot for me and that, but I think she ended up getting ill, so she kind of disappeared, and she said that she was too ill to meet up with me again. But I’m not sure if what I was talking about affected her in some way.”*

Two participants moved house and lost touch with their PSW, with one reporting:

MP17 *“I think he [PSW] was kind of worrying about me on the street so when I got somewhere to live, you know, he just, he just disappeared like that, yes.”*

This participant was homeless when he started meeting his PSW, which greatly affected his ability to engage with the programme:

MP17 *“There is too much going on, you know. Like it’s more like my brain was really on the mad side, you know, at the time. I was never really living anywhere at that time, sleeping in a garage, you know; I wasn’t having a shower, wasn’t having anything to eat, you know”*

One participant attended around half of the planned sessions before experiencing a relapse of their illness and being admitted to hospital. The interviewer asked whether the PSW had offered to visit them in hospital and they said they hadn’t:

MP39 “*He [PSW] could have come and seen me in hospital, you know, I would have liked that*.”

Another participant became unwell and informed her PSW that she was unable to continue with the sessions:

MP26 *“and I was having like really bad days and depression and things, so I sort of basically, you know, done a private text and just said like thank you for everything. I’m really sorry I’ve done poorly, but I couldn’t do anymore.”*

Two participants attended no sessions. One was caring for an unwell family member, but said that he would have liked to have participated:

MP24 “*Yes, I just had too much going on, yes, which is unfortunate because I was actually looking forward to doing the course*.”

The other participant was not offered a PSW due to a researcher error. This participant was offered PSW support once the error was discovered; however he declined the offer:

Interviewer: “*Do you remember why you decided not to take it up when we offered it later?*

MP35 *Because at the time it was offered later I was, sort of, in a combination of being in a good place and busy.”*

**Duration of sessions**

Participants reported that sessions lasted an hour, with one (MP9) reporting that sessions sometimes lasted longer. Most participants stated than an hour felt like the correct duration:

MP22 *“they were a good length, same thing, not too short, not too long.”*

**Location of sessions**

Participants met with their PSW in a variety of locations: at home, in a crisis house, at cafes, McDonalds, and in the park. No participants expressed any concerns about the location of meetings, and those people who met in public did not feel that their privacy was compromised. The choice of location seemed in some cases to be an aspect of the more informal nature of peer support:

MP15 “*You know, we always met in coffee bars so once again that’s, like, highly informal*.”

**Helpfulness of support**

12 out of 18 (67%) of participants interviewed reported that they found the programme helpful. This included all 10 of the participants who attended most or all of the sessions. The other 2 participants who found it helpful attended some of the sessions before moving house (MP31) or admission to hospital (MP39).

Participants who found the programme helpful commented on the following areas:

PSWs providing knowledge about mental illness and treatment:

MP1 “*I found out more about depression, a lot of things I didn’t actually know about.”*

MP15 “*as a patient, sometimes you feel, like with anything, that, you know, the doctor is the expert and you are the person receiving it. And you don’t necessarily have a voice. And I suppose [seeing a PSW] did let me think, well, hang on, I can voice my worries …”*

MP22 “*I wouldn't say, like, it's changed my life. Like, it has opened my eyes to a few things, you know, just from doing the book. I write down a lot more stuff now, just from doing this, you know. It's little things but personally I think it's the little things that matter in life.”*

Providing additional support and someone to see regularly:

MP3 “*if I ever felt like I was going into a crisis, I could call my peer and get a bit of advice from her about what to do*”

MP20 “*for me it’s additional support knowing that there’s another person I can talk to, yes, and that’s what I find sometimes I need*”

Encouraging additional activity:

MP9 "*I never used to go out all the time, but now there’s not a day that I'm not..."*

Help with practical activities was valued, like looking for local services (MP3), coping strategies for tinnitus (MP39), financial help (MP9) and help with housing (MP14 and MP29).

8 participants found it helpful to meet someone with similar symptoms or experiences:

MP14 “*she’s spoken about herself and she’s given me hope, big hope.*”

MP26 “*when she was explaining to me, what’s basically happened to her, it felt like a weight had been lifted.”*

A strong personal relationship with PSWs was also valued:

MP22 “*Yes, because you could tell he loved it, all this sort of stuff, you know, like he had passion for it. It wasn't because he had to be here, it was... Yes, you know, you, sort of, feel more committed really because you feel like you're letting somebody down and you wouldn't be but*....”

MP29 “*We just clicked. We sort of liked each other’s company*.”

MP30 “*She was incredibly helpful … She had very deep understanding, and deep peace within her. I'd love her as a friend*… *She got me out of my depression*.”

2 participants did not meet with their PSW: MP24 who was caring for family member and MP 35 who was not allocated a PSW due to researcher error.

**Unhelpful programme**

4 participants reported finding the whole programme unhelpful. MP5 did not like the idea of weekly meetings (see quote above). MP7 did not get on with her PSW and stopped seeing her after a few sessions:

MP7 “*I’ve just found that she’s been... probably just her personality, but trying to be, like, this mother figure to me and it’s really, really winding me up*.”

This participant also did not find it helpful to work to a timetable:

MP17 *“I don’t like people telling me what… I’ll do it this week, I’ll do it that week. And you know, I’ve always been very independent and stuff. And I just find it a bit patronizing. So I’d rather, you know, do it my own, kind of, time, and frame…”*

MP17 found the programme unhelpful because his PSW couldn’t help him with his housing situation:

MP17 “*Because I think when I met him I was like sleeping on the streets and things like that … he told me he couldn’t help me*.”

MP25 found the programme unhelpful because she only had one session with her PSW who then became unwell and was unable to continue to meet with her:

Interviewer: “*how did you find that [one] session?*

MP25 “*I found it okay. It was a bit emotional. But she did say that she would access some groups, and take me to places where they’d been through similar situations, and get more support for me. And I just felt let down by the whole thing*.”

Two further participants found the programme to be helpful overall, but did not like particular aspects of the programme. One participant found recovery-focussed work to be unhelpful when they hadn’t yet met with a psychiatrist (MP1). The other participant found the advice of the PSW to be unhelpful:

MP14 *“She told me lots of things. But I know myself, and I know what I need.”*

**Relationship with PSW**

11 out of 16 (69%) participants who met with their PSW described their relationship as good, with many participants expressing that their relationship felt akin to a friendship:

MP30 *“We became friends and we spoke the same language.”*

MP22 *“It was nice to meet somebody who’s just not a professional … somebody who actually suffers from it on a day-to-day basis.”*

The remaining 5 participants had varying experiences. MP5 only had one session with their PSW as he did not like weekly meetings. He commented:

MP5 “*I think I’d prefer to do it [the workbook] on my own. I don’t think I’m really into having a peer worker*.”

MP17 got on okay with his PSW, although found him unhelpful (see quote above). MP15 spoke about feeling uncertain about the nature of the relationship:

MP15 *“I didn’t have that professional distance where I was telling an expert or a very close friend that I felt I could, you know, trust with that kind of information.”*

MP25 only had one meeting before her PSW became unwell and said that seeing a peer was not always encouraging, due to the continuing nature of the condition:

MP25 *“Because she said, she was still dealing with it. So, that made me feel a bit hopeless, that she had been through it, and she was still trying to cope with it.*

MP7 did not get on with her PSW and stopped seeing her after a few sessions (see quote above).

**Comments on the workbook**

Positive comments were that it’s useful to have ideas about recovery on paper (MP3) or to look back at goals (MP29) and that it was useful for recovery and thinking about triggers or keeping well (MP20, MP22);

One participant liked the blank pages for their ‘paintings’ (MP30), but another didn’t like having blank pages (MP35), commenting that this “*might be a disincentive to fill it in*.”

Four participants didn’t find all of the sections in the workbook ‘relevant,’ didn’t find it useful or were not ready to think about recovery-based content (MP1, MP14, MP17, MP24). Others found the questions ‘repetitive’ (MP1, MP9, MP22) or didn’t want the written ‘reminder’ of how things had been (MP31).

Suggested improvements were for an online version because ‘*things change*’ and previous comments get ‘*scribbled out*’ (MP7); the book should be smaller (MP9, MP15) whereas another commented that the size was ok (MP29).

**Improvements to programme**

Eight participants suggested improvements to the programme. Four participants (MPs 1, 3, 15 and 30) said that they would have wanted more sessions. Other suggested improvements were to start a participant meet-up group (MP1) or website (MP3); having a set time for PSWs to contact participants (MP7); clarification about whether the PSW can continue to meet with the participant after the programme (MP15); A4 size workbook (MP15); PSW to support with housing problems (MP17) and recruiting PSWs who are likely to stay well during the programme (MP25). Finally, MP35, who was not allocated a PSW in error, requested that administrative procedures are improved so that this does not happen again and that support would be offered when needed: “*in a timely manner; strike while the iron’s hot sort of thing*.”
