# Supplementary material for: Development of a peer-supported, self-management intervention for people following mental health crisis
Source: BMC Res Notes. 2017 Nov 9;10:588. doi: 10.1186/s13104-017-2900-6 (PMC5680762; doi:10.1186/s13104-017-2900-6)
Supplement: Supplementary file 7 — Additional file 7: DS9. Pilot trial (stage 5)—main themes from PSW focus group. [file 13104_2017_2900_MOESM7_ESM.docx]

**DS9: Pilot trial (stage 5) – Main themes from Peer Support Workers’ focus group**

A follow up focus group was completed with all five PSWs who provided peer support in the randomised controlled trial pilot.

Overarching themes

Better contact with CRT this time around, but still not fully integrated into NHS structures

*“No one [at the CRT] says hello. I don't know any of their names, like, and I think just some things like that could have just been done a bit better”*

Supervision issues

Supervision from the CRT felt very different from the supervision provided by the research group in that it felt less recovery focused and supportive to the peer experience. However, it did mean greater ease of contact when there was a crisis situation.

Dilemma of being supervised by staff who may have clinical responsibility for the participant again in the future – can the PSW ethically share information with these staff?

Supervision not always that helpful, clinical focus of CRT staff leads to meaningful issues not really being discussed and a lack of genuine dialogue about how to work with participants.

Role of PSW

NHS staff could learn from work done by PSWs

PSWs do personalized work which CRT should carry out (but which it can fail to do)

*“But I think it's that willingness also then to actually have the confidence to be able to kind of actually spot when there are practical issues and help move those forward. I think that's what the crisis teams just don't seem to be doing on face value. Because, you know, I think I've... what, six or seven people I've seen, and probably about half of them have had very unresolved really practical issues, which I'm really surprised that, you know, those hadn't been picked up by a crisis... well, probably not surprised, really, that it hadn't been picked up by a crisis team. Because they were issues which should have been moved on or should have been, you know, should have been spotted, but they weren't spotted. And I think what we can bring is, I suppose, the more completeness, you know, and a willingness to kind of engage in whatever area...”*

Commitment PSWs show to their role and personal understanding of difficulties in achieving recovery allow them to provide a different service to that provided by CRT

*“You turn up each time you're supposed to be there and you don't sort of throw them out with the dishwater. You know, the fact that they're not there on one occasion doesn't mean that you don't try the next week. And that message is there. So, you can't make it better. The big thing that we all want to do is to make it better for someone, but actually you can't. You can only work with them where they're at and what they're able to do, and what you're able to do. They're all bits and pieces. But you're working together and you're trying to make... you're trying to achieve something together, and I think that just feels important. And helping people to think and perhaps, I don't know, your experience, my experience, and my experience, say, will be different to somebody else's, but just bringing that long and trying to think things through together and yes, just understanding... I mean, the whole thing about peer support, isn't it, it's just different”*

PSWs must know about practical issues as part of their role, but difficult to find information sometimes

*“I think to me, one of the kind of the things is it has reinforced with me how much additional knowledge it's useful to have around the edges of the booklet, let's put it that way. So I think some knowledge of benefits is useful. You're not there to be a benefits expert, but I think sometimes there's information which is useful to know, at least to point somebody, look, you might need to talk to somebody about this.”*

*“And I just think that's crucial. I think it's absolutely crucial to, like, yes, just to be having that knowledge is... it's kind of been talked about before in supervision saying, oh, we'll get a list of things but it's never actually materialised. But I think those things, like, obviously, like, the training was really useful and had loads of things, but it did miss this side of just, like, practical things, basic knowledge of how, like, benefits and housing works, and, you know, just having a list of borough services, you know. I just, I've kind of wanted that the whole time, really.”*

Flexibility essential

*“We've got to have a lot of flexibility, yes. That... I think that's what makes it a very challenging task. It's almost like saying, well, you could be good at helping somebody work through this booklet, but actually if you haven't got those other bits around the edge, which you can then... perhaps you don't know in huge depth but you know sufficiently and you spend the time to help move somebody on, or help them move on, I should say, in that area...”*

Difficulty of ending relationship with participant

*“But I think it's that deep caring, which I think, you know, can be a bit challenging at times where you get to care about somebody so much that actually, you know, when you get to the end... actually, I haven't got to the end with any of my people yet, so interesting to see, but where you get to the end and you think, actually, I don't want to leave this person. I mean, there's one person from the previous thing where I can still be in the vicinity of where they are and I think of them. Didn't happen with the other two, but I think that's because this particular person, I suppose, I had to give, you know, that extra bit, even that extra, extra bit of myself to try and... and actually I suppose that's how it affected me in the long run, you know, is I still think about them. I still think, oh, I'd love to go and ring that bell and say, you know, but no, I'm not going to.”*

Signposting a key part of role

Comments on workbook

Repetitive at times

*“But the degree of repetition in the booklet has kind of really still struck me, but I kind of understand why that is, because you can kind of enter it at different points. But that does raise a challenge for when you kind of... if you want to work through it... [unclear], it was interesting, the one person I was looking at things very kind of specifically and we were kind of looking at particular pages, and lo and behold, what he wrote down there, he had already written down earlier. And I just... I don't know, it just seems quite a lot of repetition in it still. But then, you know, maybe that's something you can't get away from and it's always been designed as something you can dip into the bits which are most applicable for you at that particular moment of time.”*

Acts as a framework for discussion

*“It's a framework, which some people I think do find it useful, you know, undoubtedly to fill something in, you know. But, you know, it's almost like, it's kind of like a framework to hang discussion on, to always kind of have in the back of your mind that it's kind of about those kind of issues.”*

May not work for everyone, but useful to have

*“Also everyone works around these people very differently and the book is just one way of doing it. So ultimately some people just will not, you know, not be into it. So, you know, because everyone works differently. But I think it's good that it's there as, you know, it's an option. And like you say, talking about issues in the book is really important anyway, so even if it's not committed to paper, it's still, yes, good to be there.”*

Writing something down can be challenging, not due to literacy issues but because it implies a level of commitment which the person may not be ready to make:

*“I think there for some people that it's difficult to commit by writing down something. So when something goes down on a piece of paper, and it can't be taken away...”*
